# Adolescent Substance Use and the Brain: Behavioral, Cognitive and Neuroimaging Correlates

**DOI:** 10.3389/fnhum.2020.00298

**Published:** 2020-08-04

**Authors:** Shahnaza Hamidullah, Hayley H. A. Thorpe, Jude A. Frie, Richard D. Mccurdy, Jibran Y. Khokhar

**Affiliations:** Department of Biomedical Sciences, University of Guelph, Guelph, ON, Canada

**Keywords:** adolescence, youth, addiction, drug, abuse psychology, special population

## Abstract

Adolescence is an important ontogenetic period that is characterized by behaviors such as enhanced novelty-seeking, impulsivity, and reward preference, which can give rise to an increased risk for substance use. While substance use rates in adolescence are generally on a decline, the current rates combined with emerging trends, such as increases in e-cigarette use, remain a significant public health concern. In this review, we focus on the neurobiological divergences associated with adolescent substance use, derived from a cross-sectional, retrospective, and longitudinal studies, and highlight how the use of these substances during adolescence may relate to behavioral and neuroimaging-based outcomes. Identifying and understanding the associations between adolescent substance use and changes in cognition, mental health, and future substance use risk may assist our understanding of the consequences of drug exposure during this critical window.

## Introduction

Adolescence is characterized by a series of developmental changes occurring roughly between 10–19 years, with the timing of onset highly impacted by social, cultural, and nutritional influences (Spear, 2000). During this time, the body experiences increased production of gonadal steroids that contribute to growth and sexual development (Spear, 2000). Additionally, a vast array of neurodevelopmental changes occur during this time, including cortical thinning and gray matter volume (GMV) reductions, increases in white matter volume, synaptic pruning, and reorganization within cortical and limbic regions (Schneider, 2013; Spear, 2014; Jaworska and MacQueen, 2015; Dumontheil, 2016; Thorpe et al., 2020). These neurodevelopmental changes give rise to characteristic behaviors during adolescence, such as improvements in cognition and executive functions; increases in reward sensitivity, novelty-seeking, risk-taking behavior; as well as a tendency to spend more time with peers (Spear, 2000; Choudhury et al., 2006; Romer, 2010). Some of these behavioral characteristics, in turn, contribute to a greater likelihood of initiating substance use (Lisdahl et al., 2018). The temporal overlap between substance use initiation and the vulnerable neurodevelopmental windows makes this an important period to study (Spear, 2000; Thorpe et al., 2020).

Substance use (used broadly to include alcohol and other drugs) by adolescents remains a significant public health concern. According to the most recent National Epidemiologic Survey on Alcohol and Related Conditions, more than 50% of substance use initiation cases occur between the ages 15–19 (Blanco et al., 2018). Moreover, an earlier age of onset of use is significantly associated with the risk of developing a substance use disorder later in life (Taioli and Wynder, 1991; Viner and Taylor, 2007). While the prevalence of substance use has declined in recent years from historical highs, recent surveys show that there have been some specific increases in the past year and that some concerning patterns may be emerging. According to the University of Michigan’s Monitoring the Future Survey in 2019, the prevalence of cannabis use as well as any illicit drug use in students in grades 8–12 have remained consistently high across prior decades (Johnston et al., 2020). Furthermore, nicotine vaping continued to be a concern with over one in three grade 12 students reporting past-year use (with 25.5% of these students indicating past month use), and this prevalence remains substantially higher than other forms of tobacco, including cigarettes, which continues to decline (Johnston et al., 2020). Another emerging trend from the survey suggested that the declining trends in alcohol use and binge drinking may be leveling off (Johnston et al., 2020). Despite the declines from historical highs, by the end of high school, four out of every 10 students reported consuming alcohol in their lifetime. In addition to the increased risk for future substance use, adolescent drug use can also negatively impact ongoing neurodevelopment, which might contribute to the risk for cognitive impairments and psychopathology. A growing body of research predominantly consisting of findings from magnetic resonance imaging (MRI) studies is beginning to unravel the structural and functional changes associated with these clinical outcomes.

This review will outline the cognitive, psychopathological, and future drug use related associations with adolescent substance use, especially related to the emerging trends in this use that have not been addressed in previous reviews. We will also present brain-imaging based neurobiological correlates of these findings when applicable, providing a unique perspective on these associations and potential interactions between behavioral and neural domains. While the specific behaviors under each of the reviewed domains may differ between the drug classes (depending on the availability of research findings), this approach helps to contrast the similarities and differences between the different drugs. We focus on findings from studies of substances most commonly used during adolescence, namely tobacco and e-cigarettes, alcohol, and cannabis (Johnston et al., 2020); while other less prevalent drug classes (e.g., stimulants, ecstasy) are not addressed in this review (for a review see Squeglia et al., 2009a), we chose to include opioids and drug co-use as additional drug classes due to the lack of existing syntheses on these topic. Although brain development continues well into adulthood (Spear, 2014), we limit this review to studies using adolescent sample populations with a mean age of 19-years-old or lower to capture the potential effects of drug use during the most dynamic stages of post-childhood development. This review comes at a time of recreational cannabis legalization and decriminalization by government bodies across the globe despite our somewhat incomplete understanding of its causal impacts on the developing brain alone, or in combination with other drugs commonly used by youth. Importantly, we also summarize the currently available findings surrounding the potential consequences of vaping, which has quickly become one of the most common methods of nicotine and cannabis delivery in youth, one that is still under-represented in the literature to date.

## Tobacco and E-Cigarettes

**Figure 1:**
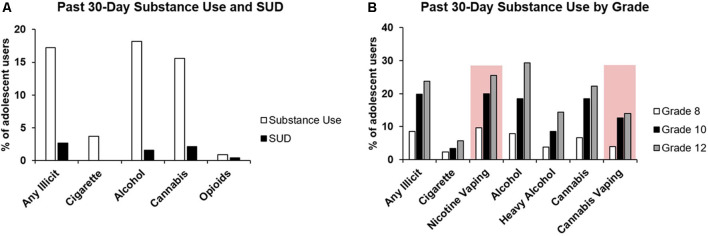
Prevalence of substance use and substance use disorder in adolescents. **(A)** Collated data from the 2017 National Survey on Drug Use and Health, 2018 National Survey on Drug Use and Health, and 2019 Monitoring the Future Survey showing the past 30-day substance use by U.S. adolescents, along with the reported percentage of adolescents with specific substance use disorders (Substance Abuse and Mental Health Services Administration, 2018; Substance Abuse and Mental Health Services Administration, 2018; Johnston et al., 2020). **(B)** Adolescent substance use by school grade (8, 10, 12) as per the 2019 Monitoring the Future report (Johnston et al., 2020). All categories represent self-reported substance use in the past 30 days except for heavy alcohol use (five or more drinks in a row) in the past two weeks. Emerging substance use behaviors (i.e., nicotine and cannabis vaping) are highlighted by a red box.

In 2017, it was estimated that 4.9% of adolescents in the United States aged 12–17 were current users of tobacco products, including cigarettes, cigars, smokeless tobacco (i.e., snuff, chew), and pipe tobacco (Substance Abuse and Mental Health Services Administration, 2018). Recent estimates suggest 3.7% of adolescents regularly use cigarettes (Figure 1A; Johnston et al., 2020). These estimates, along with results from the US National Survey on Drug Use and Health, indicate that the prevalence of tobacco use is at its lowest levels since 1991 (Substance Abuse and Mental Health Services Administration, 2019; Johnston et al., 2020). These declining trends in tobacco use, however, contrast with nicotine vaping rates among teens; more adolescents in grades 8, 10, and 12 are estimated to be vaping nicotine than smoking combustible cigarettes (Figure 1B; Johnston et al., 2020), and the rate of use has been steadily increasing since 2011 (US Department of Health and Human Services, 2016). In this age group, nicotine vaping is often perceived as less harmful than traditional smoking (Parker et al., 2018; Jun et al., 2019), likely contributing to the growing proportion of adolescents who experiment with, and regularly use e-cigarettes. Traditional smoking habits are initiated almost exclusively between early adolescence and young adulthood (Substance Abuse and Mental Health Services Administration, 2019), and initiating e-cigarette use in later adulthood is unlikely relative to those under the age of 25 (National Center for Chronic Disease Prevention and Health Promotion (US) Office on Smoking and Health, 2016).

Nicotine, the primary psychoactive component of cigarette smoke and e-cigarette liquid, is highly addictive and can impact brain development when its use is initiated during adolescence (Thorpe et al., 2020). Nicotine interacts with nicotinic acetylcholine receptors within the body; however, there is a paucity of studies investigating human nicotinic acetylcholine receptors activity and development in the context of adolescent smoking owing to methodological and ethical limitations (e.g., use of radioisotopes in positron emission tomography). As such, most neurobiological studies utilize structural MRI to investigate gross brain morphology; functional MRI (fMRI) to infer brain region activity based on dynamic cerebral blood flow measured through blood oxygenation level-dependent (BOLD) imaging; and diffusion tensor imagining (DTI) to investigate white matter microstructure *via* water diffusivity across axon bundles (Beres, 2017; Yousaf et al., 2018). Below, we synthesize findings from studies that suggest potential cognitive-, psychopathology-, and future drug use susceptibility-related outcomes associated with nicotine use during the adolescent period, be it through combustible cigarette consumption or e-cigarette use and relate these findings to neural correlates. Summaries of these studies can be found in Supplementary Table S1.

### Cognition

Adolescence is a period of attentional development and is characterized by impulsive and risk-taking behaviors (Romer, 2010). Several longitudinal (Treur et al., 2015; Akkermans et al., 2017) and cross-sectional (Tercyak et al., 2002; Jacobsen et al., 2005, 2007b,c) reports implicate a relationship between adolescent smoking and worsened attentional performance relative to non-smoking youth. Though not always significant (Jacobsen et al., 2007b,c), studies consistently report more symptoms of inattention in smokers compared to non-smokers that persist into adulthood (Tercyak et al., 2002; Treur et al., 2015; Akkermans et al., 2017). Performance during selective and divided attention tasks are similarly observed to be poorer in smoking adolescents compared to their non-smoking peers (Jacobsen et al., 2005, 2007b,c; Bi et al., 2017; Li et al., 2017), especially in males (Jacobsen et al., 2005), although divided, but not selective, attentional deficits may be related to nicotine withdrawal (Jacobsen et al., 2005, 2007c). Although performance deficits in some of these attentional tasks may stem from smoking-associated working memory impairments (Jacobsen et al., 2005, 2007c), findings from neural correlate studies conducted in smoking and non-smoking youth suggest that smoking behaviors impact the development and function of attentional brain circuits. Many studies have shown morphological and functional differences between smoking and non-smoking adolescents in the prefrontal cortex (PFC), inferior parietal cortex, and anterior insula that in part comprise the selective and divided attention neural circuits (Elsey et al., 2016). Gray matter loss in the cortex may be exacerbated by smoking; smokers reportedly have lower amounts of gray matter in the frontal cortex (Li et al., 2015; Akkermans et al., 2017; Chaarani et al., 2019), inferior parietal lobe (Li et al., 2015; Akkermans et al., 2017), and insula (Li et al., 2015) than non-smoking controls, and gray matter in the dorsolateral PFC (DLPFC) was negatively correlated with smoking dependency (Li et al., 2015). A recent fMRI study of adolescent smokers found that resting-state functional connectivity (RSFC) was lower between the anterior insula and the DLPFC, amygdala, and striatum of smokers compared to non-smokers (Bi et al., 2017). The activity of the DLPFC appears to be important for divided attention performance, such that greater activation of this brain region is associated with worse performance accuracy when multiple sensory modalities are required (Johnson and Zatorre, 2006), and RSFC between the anterior insula with the DLPFC and inferior parietal cortex may be reduced during acute smoking abstinence (Fedota et al., 2018). Although DLPFC activity has not been monitored during task performance in smoking adolescents, resting-state deficits in the DLPFC during minimal nicotine deprivation conditions found by Bi et al. (2017) suggest smoking-induced functional changes to networks important to divided attention, though the appearance of cognitive impairments such as those found by Jacobsen et al. (2005) may depend on smoking recency. Collectively, these results suggest that the neurotoxic effects of smoking may interfere with the normal developmental trajectory and function of attention-related brain regions and consequently manifest as attentional deficits.

Tobacco use is also suggested to have long-term impacts on inhibitory control, which could prevent future abstinence from smoking through a failure to suppress smoking urges. However, adolescents consistently report fewer withdrawal symptoms relative to adult smokers (McNeill et al., 1986; Rojas et al., 1998) and studies measuring inhibitory control and impulsivity behaviors on adolescent smoking patterns have been conflicting. Counterintuitively, some studies have found that impulsivity (Tercyak et al., 2002) and distractibility (DiFranza et al., 2007) are protective factors against current cigarette use and the loss of control over smoking relative to adolescents without symptoms of impulsivity, whereas others have identified a positive association between impulsivity and cigarette use (Leventhal et al., 2015). These inconsistent findings, as suggested by DiFranza et al. (2007), may be attributed to only some studies controlling for medication status in individuals with co-occurring psychiatric disorders affecting impulsivity such as attention-deficit/hyperactivity disorder (ADHD). Furthermore, there is conflicting evidence surrounding the association between adolescent smoking and inhibitory control performance, with one study finding that smokers commit more errors in the Go/No-Go task (Yin et al., 2016), whereas another study found that adolescent smokers do not show inhibitory control deficits during the Stop-Signal task (Galván et al., 2011) compared to non-smoking peers. However, negative correlations between successful Stop-Signal inhibition trial reaction times and BOLD activation in regions important to inhibitory control have been reported such that greater activation was associated with faster responding (Galván et al., 2011). These correlations indicate that inhibitory control regions (Goldstein and Volkow, 2002; Zhang et al., 2017) are possibly affected by adolescent smoking, supported by findings of smoking-associated abnormalities in the adolescent anterior cingulate (ACC; Lee et al., 2005; Rubinstein et al., 2011b; Bi et al., 2017; Li et al., 2017), insula (Lee et al., 2005; Jacobsen et al., 2007c; Rubinstein et al., 2011a; Li et al., 2015, 2017; Bi et al., 2017), and orbitofrontal cortex (OFC; Dinn et al., 2004; Li et al., 2015; Akkermans et al., 2017). The Go/No-Go and Stop-Signal tasks are thought to rely on unique neural correlates despite sharing a common core network, which may explain the discrepant cognitive results between studies (Zhang et al., 2017; Raud et al., 2020). Taken together, these findings suggest that baseline inhibitory control and impulsive behavior may determine the risk for adolescent smoking, and likewise adolescent smoking may be a detriment to inhibitory control processing.

Aside from potential smoking-induced deficits in attentional and inhibitory processes, there is some evidence that adolescent smoking alters intelligence. A longitudinal study of older adolescent male current smokers, former smokers, and non-smokers found that cognitive abilities related to intelligence quotient (IQ) were negatively correlated with the number of cigarettes smoked per day, that performance deficits were more pronounced in current smokers than non-smokers and former smokers, and that cognitive performance was lower in former smokers than non-smokers (Weiser et al., 2010). Among discordant smoking sibling pairs, smokers were also more likely to have a lower IQ than their non-smoking counterparts. Furthermore, future smoking was more likely in males with lower baseline cognitive scores compared to those who did not initiate smoking, suggesting lower IQ may be predictive of future smoking, which has been supported (Corley et al., 2012; Wraw et al., 2018) and contended (Batty et al., 2007) by other studies comparing childhood IQ with smoking in adulthood.

Adolescent smoking may also impact working memory in a sensory modality-dependent fashion. Auditory working memory accuracy was found to be worse in adolescent smokers compared to non-smokers (Jacobsen et al., 2005, 2007a). These auditory cognitive deficiencies were later recapitulated by the same group, which showed greater smoking-associated deficits in auditory relative to visual cognitive performance (Jacobsen et al., 2007c). These auditory working memory deficits are supported by fMRI findings suggesting that brain regions supporting auditory working memory, such as the inferior frontal gyrus and parietal lobes, show greater activation with worse task performance, suggesting network inefficiency in smokers (Jacobsen et al., 2007a). Likewise, DTI findings suggest that smoking youth have altered white matter integrity compared to non-smokers, as indicated by greater fractional anisotropy (FA), an indirect measure of axonal organization and coherence, in auditory corticothalamic tracts (Jacobsen et al., 2007b). This is in line with findings of a recent meta-analysis of smokers under 30-years-old suggesting brain-wide increases in FA compared to non-smokers, which could represent greater white matter integrity or myelination, or deleterious vasogenic swelling in these tracts (Gogliettino et al., 2016). In addition, smoking-associated differences in hippocampal (Jacobsen et al., 2007c; Rubinstein et al., 2011b) and parahippocampal (Rubinstein et al., 2011b; Li et al., 2015) function and morphology have also been reported in smoking youth, further supporting potential effects of smoking during adolescence on alterations in memory performance.

Age of initiation is an important factor in the trajectory of potential negative outcomes of smoking. Attentional (Treur et al., 2015) and working memory (Jacobsen et al., 2005) performance impairments are less pronounced in those who initiated smoking at a later age. Also, an earlier onset of cigarette use initiation and regular use are both consistently associated with heavier smoking patterns and craving in later adolescence (Stanton, 1995; Everett et al., 1999; Colder et al., 2001; Riggs et al., 2007; Dierker et al., 2012; Buchmann et al., 2013) and adulthood (Taioli and Wynder, 1991; Klein et al., 2013; Lanza and Vasilenko, 2015), as well as greater smoking cue reactivity in adulthood (Mashhoon et al., 2018). Greater cognitive deficits associated with an earlier age of smoking initiation may, therefore, reflect a unique early adolescent vulnerability to the effects of nicotine exposure and/or a cumulative impact of smoking duration on cognition. Although dependence and withdrawal symptoms are reportedly lower in adolescents compared to adults (McNeill et al., 1986; Rojas et al., 1998), dependence in smoking adolescents could lead to loss of smoking autonomy (McNeill et al., 1986; Rojas et al., 1998; DiFranza et al., 2000, 2002). One theory suggests that adolescents are at higher risk for the future negative consequences of smoking because they are less likely to experience negative feelings associated with tobacco use, and thus will continue their habits despite the known health risks, subsequently leading to more damage to the brain through the neurotoxic effects of nicotine (O’Dell et al., 2004). Considering this, early-onset adolescent smoking may cause a greater deviation in the developmental trajectory of attentional-, memory-, inhibitory control-associated brain regions than those who are late-onset users, and consequently worsen the management of withdrawal symptoms during cessation attempts. Together, these studies highlight the importance of early cessation interventions for adolescent smokers, especially for those who initiate their smoking habits at younger ages, to mitigate the potential cognitive impairments that arise from adolescent smoking as well as the known health risks associated with chronic smoking in adulthood.

### Psychopathology

#### Schizophrenia and Psychosis

Heavy nicotine dependence is prevalent in 16–46% of those in the prodromal phase of schizophrenia (Gogos et al., 2019), leading researchers to question if there is a causal relationship between schizophrenia and smoking (i.e., does smoking increase the risk for schizophrenia, or does having schizophrenia promote smoking habits to alleviate disease symptoms?), if the risk for smoking and schizophrenia share common mechanistic underpinnings, or both (Khokhar et al., 2018). The link between adolescence, schizophrenia, and nicotine use has been intensely investigated; almost all schizophrenia diagnoses occur during adolescence and young adulthood, and neurobiological systems that develop during adolescence include those that are implicated in both schizophrenia and smoking (Selemon and Zecevic, 2015). While few studies have found no (Dinn et al., 2004) or a negative association (Zammit et al., 2003) between adolescent smoking and psychosis outcomes, most studies indicate that smoking during adolescence and young adulthood is associated with increased risk for the development of schizophrenia (Weiser et al., 2004; Myles et al., 2012b; McGrath et al., 2016; Mustonen et al., 2018). This increased risk is especially prominent in individuals who engage in heavy smoking behaviors (Weiser et al., 2004; Mustonen et al., 2018) and initiate smoking during early adolescence as compared with older youth (McGrath et al., 2016; Mustonen et al., 2018). Importantly, unaccounted for confounders in these studies may contribute extensively to the observed relationship between smoking onset and future psychotic experiences (Jones et al., 2018). However, the age of smoking onset does not appear to alter the temporal course of psychosis development, as a meta-analysis found that while an earlier age of smoking onset predicted diagnosis, smoking status did not predict an earlier disease onset (Myles et al., 2012a).

Although the etiology of schizophrenia is complex and disrupted the development of many brain regions has been implicated in its emergence, neurobiological abnormalities and cognitive impairments associated with adolescent smoking overlap with those observed in schizophrenia. For instance, patients with schizophrenia present with deficits in cognitive processes such as inhibition, attention, and working memory, and show the aberrant activity of brain regions such as the DLPFC, ACC, and parietal lobes, all of which are implicated in adolescent smoking effects (Selemon and Zecevic, 2015). As previously mentioned, adolescence is a critical period of cortical development, and gray matter loss occurs into adulthood as synapses are pruned. Cortical gray matter reductions undergo steeper declines in those with schizophrenia than healthy individuals, suggesting a link between synapse refinement and development of the disease (Selemon and Zecevic, 2015). Since cortical thickness and GMV is lower in smoking adolescents (Li et al., 2015; Akkermans et al., 2017; Chaarani et al., 2019) and the rate of cortical thinning, though non-significant, is greater in novel smokers compared to non-smokers (Akkermans et al., 2017), it is possible that smoking may exacerbate gray matter declines in youth with a genetic predisposition to develop schizophrenia. Longitudinal MRI studies of adolescent smokers and non-smokers with schizophrenia risk (genetic or environmental) would help to elucidate the potential for additive effects of these factors on gray matter development.

#### Attention-Deficit/Hyperactivity Disorder

Like schizophrenia, problematic nicotine consumption disproportionately affects individuals with ADHD. Multiple theories, such as common mechanistic underpinnings, disease-associated predisposition, and the self-medication hypothesis, have been presented to address why this is the case (Van Amsterdam et al., 2018). While it is clear that ADHD is a risk factor in smoking initiation and dependence (see Glass and Flory, 2010), there is sparse and conflicting evidence surrounding the potential for smoking to impact ADHD trajectory and symptomology in adolescents. Some studies found that ADHD symptoms are more apparent in smokers compared to non-smokers (Dinn et al., 2004; Akkermans et al., 2017) but conflict on which symptoms were associated with regular smoking. Specifically, the longitudinal study by Akkermans et al. (2017) investigating smoking and non-smoking older adolescents with or without an ADHD diagnosis found that symptoms of inattention but not hyperactivity/impulsivity were greater in smokers at baseline, whereas the cross-sectional study by Dinn et al. (2004) of college-aged participants that did not specifically target those with an established ADHD diagnosis found that only hyperactivity/impulsivity symptoms were more likely in smokers compared to non-smokers. Additionally, Akkermans et al. (2017) did not find that the trajectory of inattention symptom count was correlated with smoking status between study time-points. Given the paucity of studies on the topic and conflicting evidence between existing studies, there is little to suggest that smoking during adolescence exacerbates or alleviates ADHD symptoms or alters symptom trajectory. Future longitudinal studies in smoking and non-smoking adolescents with an ADHD diagnosis should be conducted to delineate if this is truly the case considering the significant overlap between smoking and ADHD, and that the self-medication hypothesis is one of the predominant theories in the field to explain why those with ADHD smoke.

Recently, ADHD research has focused on disruptions to cortical thickness and white matter development within the brain that appear to be a characteristic of the disease (van Ewijk et al., 2012; Bouziane et al., 2018; Albajara Sáenz et al., 2019). As with the brains of patients with schizophrenia, imaging studies show that cortical thickness is reduced in the brains of patients with ADHD (Albajara Sáenz et al., 2019), and smoking during adolescence may have additive effects on cortical thickness. Current evidence does not support this theory, but it is important to highlight that only one small cohort longitudinal study to date has investigated cortical thickness in ADHD-affected and non-affected smokers and non-smokers, which may not have had the statistical power needed to observe a relationship (Akkermans et al., 2017). There are also notable overlaps in white matter abnormalities seen in smokers and those with ADHD. Although the direction of differences compared to control subjects conflicted between studies, meta-analysis of white matter integrity in ADHD patients indicated that ADHD individuals have abnormal FA within tracts of the basal ganglia (i.e., caudate nucleus, anterior corona radiata, internal capsule), as well as the cerebellum, corpus callosum, and right forceps minor (van Ewijk et al., 2012). Adolescent smokers show increased FA in the corpus callosum, internal capsule, and inferior longitudinal fasciculus (Jacobsen et al., 2007b; Yu et al., 2016), as well as the corona radiata (Yu et al., 2016) and forceps minor (Jacobsen et al., 2007b) relative to non-smokers. However, the exact relationship between smoking and FA in the corpus callosum remains unclear considering FA in this region has been negatively, rather than positively, correlated with the extent of youth smoking history (Chaarani et al., 2019). These overlapping regional differences in white matter integrity between young smokers and those with ADHD could suggest that the ADHD brain is differentially sensitive to tobacco’s potential effects on white matter compared to those without ADHD. Interestingly, Van Ewijk et al. (2015) found abnormal white matter characteristics in both smoking and ADHD individuals, albeit in opposing directions; that is, lower FA was associated with ADHD, while FA was increased in smokers. Although the significance of these findings is unclear, it has been suggested that pre-morbid differences in white matter integrity in the brains of those with ADHD may contribute to confounding study results and may also be a causal factor in smoking initiation and maintenance as opposed to a consequence of smoking (Groenman et al., 2013; Van Ewijk et al., 2015). Also, a recent study has suggested that the developmental trajectory of white matter during young adolescence (10- to 12-years-old) is only reduced in those taking medications to treat symptoms of ADHD, but not medication-naïve patients (Bouziane et al., 2018). Prior and current treatment of ADHD with medication should, therefore, be included as a variable in investigations of white matter integrity in the ADHD brain of adolescent smokers, as it is unclear whether there is a synergistic effect of smoking and medication history on white matter microstructure across the span of adolescent brain development.

#### Depression and Anxiety

Studies reliably point to an association between adolescent smoking with depression. Depression and depressive symptoms are consistently observed in smoking adolescents compared to their non-smoking peers (Wu and Anthony, 1999; Goodman and Capitman, 2000; Albers and Biener, 2002; Jacobsen et al., 2007c; Needham, 2007; Ilomäki et al., 2008; Audrain-Mcgovern et al., 2009; Morrell et al., 2010; Slomp et al., 2019). Most studies of this age group found smoking positively predicted the development of depression and depressive symptoms (Brown et al., 1996; Stein et al., 1996; Choi et al., 1997; Goodman and Capitman, 2000; Windle and Windle, 2001; Albers and Biener, 2002; Brook et al., 2002, 2004; Galambos et al., 2004; Duncan and Rees, 2005; Rodriguez et al., 2005; Boden et al., 2010; Moon et al., 2010; Jamal et al., 2011; Beal et al., 2014; Gage et al., 2015), but not all findings have supported this association (Wang et al., 1996; Repetto et al., 2005; Clark et al., 2007; Munafò et al., 2008; Hu et al., 2011; Strong et al., 2014). Importantly, the relationship between smoking and depression in adolescence has been suggested to be bidirectional, such that baseline depression contributes to the risk for future smoking habits just as baseline smoking predicts depression (Brown et al., 1996; Windle and Windle, 2001; Galambos et al., 2004; Needham, 2007; Audrain-Mcgovern et al., 2009; Moon et al., 2010). Some studies also indicate that baseline depression is a considerable factor in the trajectory of depression symptom development in that smoking may mitigate symptom number acceleration, supporting the notion that a subgroup of adolescents smokes to self-medicate (Rodriguez et al., 2005; Needham, 2007; Audrain-Mcgovern et al., 2009). In comparison to depression and depressive symptoms, there is little evidence to suggest adolescent smoking is a predictor of future anxiety (Brown et al., 1996; Gage et al., 2015), but one retrospective, cross-sectional study did observe that an earlier onset of smoking (<15-years-old) was associated with an earlier anxiety diagnosis compared to late-onset smokers (Jamal et al., 2011). However, the cross-sectional and retrospective nature of this study, and that it only included participants that smoked before diagnosis, is a considerable limitation of this finding in concluding the relationship between tobacco use and anxiety disorder. As is the case with the other psychopathologies associated with adolescent smoking, the relationship between smoking, depression, and anxiety is unclear, with evidence supporting that smoking is a causative factor in the development of depression and anxiety, that pre-existing poor mental health facilitates smoking behavior, or that there is an underlying predisposition for smoking, depression, and anxiety to arise independently of each other.

A prevalent theory in the field of smoking, depression, and anxiety is that adolescents smoke to self-medicate, although some argue against this idea (Boden et al., 2010; Beal et al., 2014). Monoamine systems implicated in depression may be indirectly modulated by nicotine’s effects on cholinergic neurotransmission differentially in baseline depressed and non-depressed youth (Dao et al., 2011; Rendu et al., 2011; Pitsillou et al., 2020). This could explain why smoking is generally associated with more depressive symptoms, but deceleration of symptom progression in those with depressive symptomology preceding smoking onset (Rodriguez et al., 2005; Needham, 2007; Audrain-Mcgovern et al., 2009). This is further complicated by the dynamic development of cortical and limbic receptor expression observed during the adolescent critical period in animal models (Thorpe et al., 2020). The introduction of exogenous receptor ligands, such as nicotine may have consequences on neurotransmission that can impact youth behavior and cognition immediately, including the production of positive feelings (e.g., relaxation). However, repetitive insults to these systems by the actions of nicotine may also modulate the expression profile of neurotransmitter receptors, synthesizing enzymes, and metabolizing enzymes, ultimately changing neural activity that could contribute to the risk for depression and anxiety (Thorpe et al., 2020). The possibility of reciprocal feedback between depression and smoking should also be considered in those with smoking onset preceding depression, such that smoking may lead to the emergence of depressive symptoms that are alleviated by maintaining smoking habits.

### Future Substance Use

#### Future Drug and Alcohol Use

Chronic nicotine exposure may lead to an increased risk for neurochemical and pathological changes in the brain, and adolescent smoking is also associated with future substance use. As previously mentioned, adolescent smoking is a strong predictor of future smoking, and this risk is greater with a younger age of use (Taioli and Wynder, 1991; Stanton, 1995; Everett et al., 1999; Colder et al., 2001; Riggs et al., 2007; Dierker et al., 2012; Buchmann et al., 2013; Klein et al., 2013; Lanza and Vasilenko, 2015). Similarly, adolescent and young adult smokers are reported to consume more alcohol (Galván et al., 2011) and cannabis (Caris et al., 2009), and adolescent smoking is a predictor of future substance use (Lewinsohn et al., 1999; Dinn et al., 2004; Ilomäki et al., 2008), especially when smoking is initiated in early adolescence (Brown et al., 1996; Lewinsohn et al., 1999; Ilomäki et al., 2008). The increased risk for future substance use may be a consequence of alterations to the brain’s reward circuit (Rubinstein et al., 2011a,b; Li et al., 2015). For instance, multiple DTI studies have reported higher FA within the basal ganglia of smokers (Jacobsen et al., 2007b; Van Ewijk et al., 2015; Yu et al., 2016), including in fibers of the external capsule that terminate in the ventral striatum (Van Ewijk et al., 2015). The ventral striatum plays an integral role in motivation and reward, and the activity of dopaminergic neurons in this region is thought to be modulated by acute and chronic drug use (Volkow and Morales, 2015). Therefore, disruption of white matter tracts in this region, possibly caused by regular smoking, may leave adolescents susceptible to the rewarding potential of tobacco and other substances. This vulnerability may also extend to a future attention bias toward smoking cues; fMRI studies by Rubinstein et al. (2011a,b) suggest that even adolescent light smokers have blunted neural responses to naturally reinforcing stimuli (i.e., food; Rubinstein et al., 2011a) while simultaneously showing greater neural activation to smoking cues (Rubinstein et al., 2011b) in comparison to non-smokers.

#### E-Cigarette Use and Transition to Combustible Tobacco Smoking

Although e-cigarettes potentially offer a less harmful alternative to traditional smoking, the use of e-cigarettes may increase the susceptibility for cigarette smoking in youth that would otherwise have not begun smoking (Barrington-Trimis et al., 2016, 2018; Azagba et al., 2017; Miech et al., 2017; Soneji et al., 2017; Spindle et al., 2017; Wills et al., 2017; Parker et al., 2018; Berry et al., 2019; Vogel et al., 2019, 2020), alongside the risk for vaping cannabis (Cassidy et al., 2018; Dai et al., 2018). Adolescents are attracted to e-cigarette flavors, especially those with fruit- and candy-like tastes, and a desire to use e-cigarettes for their taste is frequently cited as a reason for use (Vogel et al., 2019; Jackson et al., 2020). Also, recent findings by Chen et al. (2018) demonstrate that smoking and non-smoking youth report urges to smoke and have greater activation of reward-related brain regions following the presentation of e-cigarette advertisements in comparison to neutral cues. As such, e-cigarette advertisements and the availability of flavored e-cigarette liquids may make use of these devices enticing to youth, encourage initiation, and subsequently lead to the transition to combustibles and other drugs.

The rising popularity of next-generation e-cigarette devices are concerning due to their ability to deliver higher nicotine concentrations in the form of nicotine salts (Boykan et al., 2019). Adolescents who use high nicotine concentration liquids with e-cigarettes are more susceptible to higher frequency and intensity of combustible and e-cigarette use in the future relative to adolescents that do not engage in e-cigarette use or use liquids with lower nicotine concentrations (Goldenson et al., 2017; Boykan et al., 2019). One study suggests that urinary levels of cotinine, a metabolite of nicotine, are higher in adolescent e-cigarette users, relative to levels observed in another study of those who consume combustibles (Benowitz et al., 2018; Goniewicz et al., 2019). However, e-cigarettes can vary widely in the amount of nicotine they deliver per puff (Wagener et al., 2017) and this finding may depend on the model of e-cigarette used by the study population. While the use of newer model e-cigarettes results in almost double the mean urinary cotinine levels compared to traditional smoking, adolescents who use any model of e-cigarettes have lower urinary cotinine levels, than those who smoke combustibles (Boykan et al., 2019). Greater nicotine delivery efficiency apparent in newer generation e-cigarettes (Wagener et al., 2017; Boykan et al., 2019) coupled with the unique vulnerability of adolescents to rewarding substances may result in youth using e-cigarettes consuming higher levels of nicotine when compared to traditional smokers. Despite their potential for harm reduction relative to traditional smoking, the high risk for adolescents to transition from e-cigarettes to combustibles and other drugs, and the possible modulation of neural activity by e-cigarette use, must be considered in future research as well as in the context of marketing and health policies surrounding these devices.

## Alcohol

According to the Monitoring the Future Survey conducted in 2019, 18.2% of adolescents in grades 8, 10, and 12 were current alcohol users (Figure 1A; Johnston et al., 2020). Of these, 1.6% reportedly had an alcohol use disorder (AUD; Substance Abuse and Mental Health Services Administration, 2019). Given the overlap between the high degree of neural reorganization and alcohol use initiation during adolescence (Zahr and Pfefferbaum, 2017), it is imperative to consider what impact this interaction may have on neurodevelopment. Alcohol acts primarily *via* γ-aminobutyric acid type-A and N-methyl-D-aspartate receptors, which regulate inhibitory and excitatory signaling within the brain, respectively (Chandrasekar, 2013; Mallard et al., 2018). An extensive body of evidence suggests that these neurotransmitter systems are affected by alcohol exposure, which may have long-lasting implications on overall neurocircuitry within the brain (Banerjee, 2014). The potential cognitive, psychopathological, and future substance use vulnerability outcomes associated with adolescent alcohol use are summarized in Supplementary Table S2.

### Cognition

Several studies have evaluated and identified potential impairments related to adolescent alcohol use on many neurocognitive domains, including attention and inhibitory control. For instance, heavy-drinking adolescents exhibit impulsive choice and attentional bias for alcohol-related cues compared to light-drinkers (Field et al., 2007). Attentional bias for alcohol-related cues was also observed in adolescent social drinkers, suggesting that attentional bias may still emerge with limited alcohol use (Melaugh McAteer et al., 2015). The association between alcohol use and attention has also been demonstrated in a longitudinal study of adolescents who were first assessed before initiation of drinking and followed over three years. In this study, greater hangover symptoms in males predicted worsening of sustained attention (Squeglia et al., 2009b). Similarly, adolescent alcohol drinking altered the developmental trajectory of impulsivity, whereby improvement in impulsivity decelerated following the onset of binge drinking (Ruan et al., 2019). Interestingly, a family history of alcoholism was shown to be protective concerning impulsivity by Jones et al. (2017). Adolescents with a family history of alcoholism who remained alcohol-naïve exhibited a greater decrease in impulsive choice across an eight-year follow-up period compared to those who went on to binge drink. Furthermore, a greater escalation of drinking was associated with greater impulsive choice in this study. The protective effect of a family history of alcoholism is not supported by earlier studies that suggest that youth with a family history of alcoholism exhibit developmental delay in executive functioning, including heightened impulsivity (see review by Cservenka, 2016). Therefore, future studies should focus on the extent to which familial alcoholism interacts with adolescent alcohol use to alter cognition. This may help uncover unique characteristics that may potentially help address some of the discrepant findings related to adolescent alcohol use throughout this section.

Supporting the cognitive differences related to attention and inhibition in adolescent alcohol users, youth who consume alcohol also exhibit neural activity differences. For instance, heavy-drinking adolescents exhibited attenuated activation in the left supplementary motor area, bilateral parietal lobule, right hippocampus, bilateral middle frontal gyrus, left superior temporal gyrus, and the ACC compared to light drinkers during a response inhibition task (Ahmadi et al., 2013). Similarly, Aloi et al. (2018) reported an association between increasing AUD severity and reduced BOLD responses within the ACC and the dorsomedial PFC during the affective Stroop task assessing emotional interference on cognitive functioning. Effects on inhibitory control may be dose-dependent as a longitudinal study of adolescents with low alcohol use did not find any impairments in the development of inhibitory control across adolescence and activation in related networks, such as the dorsal ACC, DLPFC, pre-supplementary motor area, and the posterior parietal cortex (Jurk et al., 2018). However, in a longitudinal assessment of adolescents aged 12–14 with very limited substance use histories at baseline, reduced activation in regions that largely overlapped with the Ahmadi et al. (2013) study during the same inhibitory task predicted transition into heavy alcohol use after approximately four years (Norman et al., 2011). This suggests that activation differences may predate, and possibly contribute to, the initiation of alcohol use. Another study has revealed a bidirectional relationship with reduced activation in frontal, temporal, and parietal regions during inhibitory tasks predicting future heavy drinking, and heavy drinking, in turn, predicting increased activation in frontal, parietal, subcortical, and cerebellar regions over time (Wetherill et al., 2013). Together, these findings suggest that neural vulnerabilities in regions implicated in inhibitory control predict alcohol use, and heavy drinking subsequently may lead to additional alterations. Similarly, Squeglia et al. (2014) have reported a bidirectional relationship with smaller cingulate and rostral ACC volumes at baseline predicting later transition to heavy drinking, and heavy drinking, in turn, predicting greater volume reductions in the left inferior/middle temporal gyrus and left caudate. Another study has demonstrated the reverse relationship between alcohol use and morphological differences, whereby smaller left dorsal and rostral paralimbic ACC volumes predicted later alcohol-related problems (Cheetham et al., 2014). These findings suggest that the relationship between alcohol use and neural differences is complex and on-going prospective studies (like the Adolescent Brain Cognitive Development study of the National Institute on Drug Abuse) that follow adolescents before the initiation of alcohol use and across development may help further clarify directionality.

Adolescent alcohol drinkers appear to exhibit poorer working and verbal memory (Brown et al., 2000; Hanson et al., 2011; Parada et al., 2012), suggesting that alcohol use during this critical window may predispose youth to memory impairments. However, adverse memory-related outcomes may improve after prolonged drinking abstinence. In a longitudinal study, interruption of binge-drinking patterns led to a partial cognitive recovery, with ex-binge drinkers having greater memory consolidation deficits than non-binge drinkers but fewer deficits than continued binge drinkers (Carbia et al., 2017a). In a separate analysis by this group, binge drinkers showed improvements in working memory span but maintained consistent deficits in perseveration errors (Carbia et al., 2017b). However, it is difficult to predict whether these differences in adolescent drinkers compared to their relatively abstinent peers were present before the initiation of alcohol use. In a study of adolescents first assessed at 11-years-old, working memory impairment predicted both baselines and increased frequency of alcohol use over a four-year follow-up period, while there was no evidence supporting the reverse relationship (Khurana et al., 2013). However, in adolescents first assessed before initiation of substance use, extreme-binge drinkers exhibited poorer performance in measures of verbal learning and memory despite equivalent performances at baseline (Nguyen-Louie et al., 2016). The latter study suggests that the effects of alcohol on learning and memory may be mediated by dose. Dose-dependent neurotoxicity of alcohol use is also observed in other neurocognitive domains that were previously discussed, including attention and impulsive choice (Squeglia et al., 2009b; Jones et al., 2017). Therefore, more research is needed to develop strategies to reduce alcohol intake severity that may help temper the neurocognitive consequences related to adolescent alcohol use.

Adolescent alcohol users also differ in the degree of neural recruitment during a memory task performance from non-users. For instance, during verbal recall, non-drinking adolescents show activation of the left hippocampus whereas adolescents who engage in binge drinking do not and recall fewer words. Binge drinking adolescents also show greater activation in the right superior frontal and bilateral parietal cortices, areas implicated in working memory, compared to non-drinkers, suggesting heavier reliance on alternate memory networks (Schweinsburg et al., 2010). Similarly, adequate performance in the spatial working memory task required greater response in prefrontal and temporal regions compensating for diminished activity in the bilateral cerebral areas and the left precentral gyrus in adolescents with AUD (Tapert et al., 2004). The relationship between adolescent drinking and memory may be bidirectional, as the extent of memory-related brain region activation during working memory tasks has been shown to predict future heavy drinking, and heavy drinking, in turn, predicted increased activation over time (Squeglia et al., 2012a). Female adolescents with AUD may be especially vulnerable to abnormal activity patterns, with Caldwell et al. (2005) suggesting greater compensatory activation in the temporal areas for a reduced frontal and cingulate response to the spatial working memory task. In a subsequent study, attenuated frontal, temporal, and cerebellar responses to a spatial working memory task corresponded to deficits in sustained attention and working memory in female binge drinkers. Meanwhile, male binge drinkers’ spatial working memory performance was positively correlated with activation of related brain regions and these individuals showed better spatial working performance compared to controls, suggesting an engagement of compensatory mechanisms (Squeglia et al., 2011). Aberrant neural recruitment during cognitive processes, in turn, may suggest functional compensation for differences in structural connectivity. For instance, adolescent binge drinkers exhibited lower connectivity in major white matter tracts implicated in neurocognitive functioning, whereby FA was reduced in the corpus callosum, corona radiata, superior longitudinal fasciculus, and fronto-occipital fasciculus compared to controls (Jacobus et al., 2009; McQueeny et al., 2009). These results conflict with those by Cardenas et al. (2013), who reported higher FA in the posterior corpus callosum in adolescents with AUD and did not report any regions with lower FA according to drinking status. Since higher FA in the corpus callosum was not related to any measure of alcohol use, it might predict vulnerability to AUD, rather than being a direct consequence of alcohol use. Lower FA reported by the former studies may suggest alcohol’s toxic effects on white matter microstructure as a longitudinal assessment of adolescents aged 14–19 revealed drinking-associated blunted white matter microstructure development, evidenced by decreased FA in the left caudate and inferior frontal occipital fasciculus over than years (Luciana et al., 2013). The participants in this study had no experience with alcohol and did not have any significant premorbid differences at the baseline assessment.

Morphological differences in alcohol-using adolescents relative to abstinent adolescents have also been observed in brain regions implicated in neurocognitive functioning, such as smaller hippocampal, PFC, and cerebellar volumes, as well as thicker frontal cortices (De Bellis et al., 2000, 2005; Nagel et al., 2005; Medina et al., 2008; Squeglia et al., 2012b; Lisdahl et al., 2013), but the directionality of these findings is debated. In one longitudinal study of baseline “alcohol-naive youth” aged 12–21, and another of youth aged 18–23, heavy drinkers exhibited accelerated gray matter loss in the superior frontal gyrus, caudal middle frontal gyrus, and rostral middle frontal gyrus (Pfefferbaum et al., 2018), as well as in the parahippocampus (Meda et al., 2017) compared to no/low drinking controls over two years. A similar observation was made by Squeglia et al. (2015) in lateral frontal and temporal GMV in addition to attenuated white matter growth of the corpus callosum in heavy adolescent drinkers who were followed over eight years. Other studies have demonstrated the reverse relationship between adolescent alcohol use and morphological differences, whereby thinner DLPFC and inferior frontal cortex (Brumback et al., 2016), and higher GMV in the caudate nucleus and the left cerebellum (Kuhn et al., 2019) predicted later increases in alcohol use and alcohol-related problems.

The age of drinking onset may also have important implications for future cognitive and neurobiological abnormalities. An earlier age of first drinking onset predicted worse psychomotor speed and visual attention functioning, but only when the model accounted for drinking duration (Nguyen-Louie et al., 2017). Consistently, participants with an earlier age of weekly drinking onset performed poorer on measures of cognitive inhibition and working memory than those with a later onset age. In light of this evidence, it is suggested that early onset of drinking increases the risk for alcohol-related neurocognitive vulnerabilities and that initiation of alcohol use at younger ages appears to be a risk factor for poorer subsequent neuropsychological functioning. The impact of early adolescent alcohol use upon later working memory was also observed in a larger study of 3,300 participants, with the frequent/binge drinking group displaying impaired working memory at three-year follow-up (Mahedy et al., 2018). While each of the above studies attempted to control for confounding variables, including comorbid substance use, sociodemographic status, and baseline neuropsychological performance, the impact of these confounds was mixed across studies. Nevertheless, even after controlling for these variables, the association between earlier alcohol use and poorer neurocognitive performance remained across both studies. The variability in confounding influences and the different neuropsychological measures taken across studies highlight the need for high-quality, long-term prospective cohort studies with standardized measures to better understand the lasting consequences of adolescent drinking.

### Psychopathology

#### Depression and Anxiety

Several studies have investigated the association between psychiatric illness and alcohol use (see detailed reviews: Fiorentini et al., 2011; Addington et al., 2014). However, whether this association is causal or arises from shared pathophysiology has remained difficult to parse (Khokhar et al., 2018). This notion is further supported by a comprehensive, prospective longitudinal study of participants interviewed from ages 16 through 30 that showed a high prevalence of comorbidity of major depressive disorder (MDD) and AUD. Prospectively, adolescent AUD predicted early adult MDD and early adult MDD predicted adult AUD, suggesting that MDD and AUD are inter-related in a complex manner (Brière et al., 2011). This association has been shown at even sub-clinical levels of alcohol use, with adolescent alcohol use at the age of 13–15 predicting depression at age 17 (Edwards et al., 2014). Interestingly, another study has suggested that the relationship between alcohol use and depression may be mediated by a specific measure of alcohol involvement, whereby problematic use (defined by adverse consequences of alcohol use), but not alcohol intake (defined by the level of alcohol consumption) predicted young adult MDD (Mason et al., 2008). Self-reported alcohol use in adolescence has also been prospectively associated with hypomanic/manic symptoms at age 23 (Fasteau et al., 2017); however, these results solely relied on self-reported alcohol intake and problematic use and will need to be confirmed in future studies with more robust designs. Although the neural basis for the association between adolescent alcohol use and mood disorders has been largely unexplored, AUD symptom severity in adolescents was associated with increased amygdala responses to emotional compared to neutral stimuli (Aloi et al., 2018). However, the directional implications of these findings on the relationship between alcohol use and mood disorders are unclear, highlighting the need for more studies to identify neural markers to help characterize their comorbidity. For instance, activity within neural circuitry that underlie both alcohol use and mood disorders, such as the reward circuit (Russo and Nestler, 2013), in response to paradigms measuring emotional processing should be assessed through neuroimaging techniques.

Socially anxious adolescents have been shown to use alcohol to cope with their symptoms, supporting the self-medication hypothesis (Blumenthal et al., 2010). Furthermore, in a recent large cohort longitudinal study that tracked girls aged 13–17, higher baseline depression severity predicted an increased likelihood of future alcohol use. There was also evidence for an inconsistent, reciprocal relationship with the consumption of one full drink at ages 14 and 16 predicting decreased depression in the next year. However, the latter finding should be interpreted with caution as this association was inconsistent across time and low levels of alcohol drinking are not necessarily pathological and may constitute normative behavior among adolescents (Schleider et al., 2019). A bidirectional relationship has also been reported between alcohol use and internalizing symptoms (e.g., anxiety and depressive symptoms) among adolescents who were prospectively assessed from age 14–16, where alcohol use or internalizing symptoms at age 14 predicted the other at age 16. Another important finding emerged from this study when internalizing symptoms were examined in clusters related to either anxiety or depression. While the Anxious Arousal scale showed a consistent reciprocal relationship with alcohol use, the association between alcohol use and Anhedonic Depression disappeared after controlling for delinquency, highlighting that symptoms of anxiety and depression in the internalizing domain are not interchangeable, which should be considered in future studies. There was also variation within symptoms unique to anxiety as measures from the Anxiety scale was not associated with alcohol use, contrasting what was observed with the Anxious Arousal scale (Parrish et al., 2016). This is consistent with another study showing that while early generalized anxiety symptomology predicted an increased risk for initiation of alcohol use, separation anxiety symptomology predicted decreased risk (Kaplow et al., 2001). It is also important to consider co-occurring externalizing symptoms (e.g., aggression and impulsivity) when assessing the relationship between alcohol use and internalizing symptoms, as externalizing symptoms have been previously shown to mediate this relationship (Colder et al., 2017). Current evidence relating to the association between alcohol and mood disorders is mixed with some supporting the self-medication hypothesis, while others suggesting that adolescent alcohol use may be a risk factor for developing mood disorders.

### Future Substance Use

Adolescent alcohol drinking may also contribute to the risk for subsequent alcohol or drug use and dependence in adulthood; adolescent binge drinking predicts an increased risk of adult alcohol dependence, persistent cannabis, and other illicit drug use (Viner and Taylor, 2007; Pampati et al., 2018). The association between early alcohol use and subsequent alcohol-related problems has been further supported by data drawn from two large population studies conducted in two countries with distinct alcohol use policies and cultures. After controlling for a comprehensive number of potential confounders, both early-onset drinking and early onset of excessive drinking in adolescence (aged 14–16) were related to increased risk of alcohol-related problems when assessed at 18- to 25-years-old (Enstad et al., 2019). Impaired decision-making and underlying neural mechanisms in adolescent alcohol users may mediate the relationship between alcohol use and future substance use vulnerability. For instance, adolescent binge drinkers cross-sectionally exhibited poorer performance compared to controls in the Iowa gambling task used to assess effective decision-making, and higher activity in regions implicated in the emotional and incentive-related aspects of decision-making, such as the amygdala and insula. Similarly, connectivity between the OFC and amygdala predicted increases in alcohol use and increased connectivity between these regions has previously been shown to be protective against risk-taking (Peters et al., 2017). Activation differences in response to risky decision-making may both predict and be a consequence of adolescent alcohol drinking. While adolescent binge drinkers showed reduced activation in the dorsal caudate during risky decision-making, reduced frontoparietal activation in binge drinkers was present before they initiated alcohol use (Jones et al., 2016). In another study, an opposite pattern of increased activation in the nucleus accumbens, precuneus, and occipital cortex during risky decision-making predicted earlier initiation of binge drinking (Morales et al., 2018).

Adolescent binge drinking may also alter neural activity during reward processing, with Aloi et al. (2019) showing a cross-sectional association between AUD symptom severity and reduced activity in the posterior cingulate cortex and the striatum. Furthermore, among adolescents who were alcohol-naïve at baseline, those who transitioned into binge drinking after a two-year follow-up period exhibited reduced activity in the left cerebellum compared to controls during reward processing (Cservenka et al., 2015). This cerebellar activity was negatively associated with the average number of drinks consumed/drinking days, suggesting a dose-dependent effect. Differential activation patterns in reward-related regions may also predict increases in alcohol use from age 16–18 in a gender-specific manner; higher ventral striatum activity during reward anticipation was observed in boys, and higher dorsomedial PFC activity and decreased ventral striatum activity during reward anticipation was found in girls (Swartz et al., 2020). Greater activation to alcohol cues in adolescent alcohol users have also been reported, indicating a more intense desire and craving for alcohol, potentially putting them at risk for greater alcohol use in the future (Tapert et al., 2003; Dager et al., 2014; Brumback et al., 2015). Together, these findings suggest that neural markers may both predict alcohol use initiation, and also be a consequence of alcohol’s neurotoxic effects on reward circuitry; these differences may ultimately predispose adolescent alcohol users to excessive drinking in the future. However, research investigating adolescent alcohol use and vulnerability to alcohol and other drugs is scarce and requires considerable attention.

## Cannabis

In 2019, approximately 15.6% of U.S. adolescents were current users of cannabis, making it the second most commonly used substance by this age group (Figure 1A; Johnston et al., 2020), and one that requires further attention. Adolescence marks a period in which extensive cortical reorganization and synaptic pruning occur, and mounting evidence points to chronic cannabis use interfering with this process (Renard et al., 2014). Δ^9^-tetrahydrocannabinol, the primary psychoactive ingredient of cannabis, acts primarily as a partial agonist at the cannabinoid type 1 receptor. Given that cannabinoid type 1 receptors are widely expressed throughout the brain, structural and functional consequences of cannabis exposure are a subject of interest (Pertwee, 1997). Herein, we review the possible consequences of cannabis use during adolescence related to cognition, psychopathology, and future substance use risk, and studies investigating these associations are summarized in Supplementary Table S3.

### Cognition

Numerous studies have suggested that adolescent cannabis users are at a heightened risk for adverse cognitive outcomes (see review by Lubman et al., 2015). For instance, cross-sectional studies have reported that adolescent cannabis use is associated with impairments in inhibitory control and attention (Harvey et al., 2007; Lane et al., 2007; Medina et al., 2007a). A longitudinal assessment by Infante et al. (2020) supports this relationship as greater lifetime adolescent cannabis use over 14 years was associated with impairments in inhibitory control and visuospatial functioning. Inhibitory control impairments may be, in turn, related to increased connectivity between the parietal and cerebellar regions, which comprise part of the inhibitory circuit (Behan et al., 2014). Adolescent cannabis users also exhibited hyper-activations in DLPFC and parietal regions during a Go/No-Go task in the absence of group differences in performance, instead suggesting functional compensation (Tapert et al., 2007). The effects of cannabis use on attention in adolescence may be dose-dependent. In a recent large cross-sectional study of adolescents aged 14–21, frequent, but not occasional cannabis users exhibited poorer sustained attention compared to non-users. Interestingly, earlier age of onset of cannabis use appeared to be a risk factor for sustained attention deficits in occasional cannabis users (Scott et al., 2017). This dose-dependency is also apparent in fMRI studies with adolescent chronic cannabis users exhibiting impairments in executive attention and greater activation of the right PFC compared to non-using controls (Abdullaev et al., 2010). Also, cannabis users may be more vulnerable to the adverse effects of cannabis on attention compared to other executive functions as Hanson et al. (2010) showed that working memory and verbal learning deficits improved after three weeks of abstinence in cannabis users, while attention deficits persisted. This study highlights the importance of considering the periods of abstinence from cannabis in cross-sectional studies that differ from one study to another, making it difficult to disentangle acute and lasting effects of adolescent cannabis use on cognition. Therefore, harmonization of protocols relating to the period of abstinence is necessary, in addition to assessing the effect of abstinence from cannabis longitudinally.

Although not specific to inhibitory control or attention, adolescent cannabis users also displayed larger cerebellar vermis volumes compared to controls, which was associated with poorer executive functioning assessed by subsets of the Delis-Kaplan executive function test. This suggests that morphological differences in brain regions may underlie abnormalities related to deficits in higher-level cognitive skills (Medina et al., 2010). Female adolescent cannabis users may be at a greater risk for such differences as Medina et al. (2009) reported larger PFC volumes in female cannabis users, with smaller PFC volumes predicting better executive functioning among cannabis users. Reduced right medial PFC volume (Churchwell et al., 2010) and greater left hippocampal volumes (Ashtari et al., 2011) have also been observed in adolescent heavy cannabis users; however, functional correlates of these morphological differences have yet to be studied in adolescent heavy cannabis users. The hippocampal volume findings conflict with Weiland et al. (2015), who showed that adolescent daily cannabis users did not differ from non-users in hippocampal volumes. Similarly, Scott et al. (2019) reported non-significant differences between frequent and occasional cannabis users, as well as non-users in global or regional brain volumes, cortical thickness, and gray matter density. These findings suggest that adolescent heavy cannabis users may be at a heightened risk for impairments in neurocognitive functioning, and future studies should focus on investigating the functional correlates of the structural differences observed in heavy cannabis users.

Cannabis use during adolescence is also associated with deficits in intelligence. In adolescents first assessed at 13 years of age before the onset of cannabis use and again at 20-years-old, poor short-term and working memory predicted earlier age of onset of cannabis use. Conversely, an earlier age of onset and more frequent use during adolescence was associated with declines in performance in verbal IQ as well as trial and error learning and conditional association learning (Castellanos-Ryan et al., 2017). Similarly, adolescent cannabis use was associated with greater IQ decline and working memory impairments and cessation of cannabis use did not restore neurocognitive functioning (Meier et al., 2012). However, findings regarding adolescent cannabis-associated declines in IQ remain conflicting with one study showing no evidence for IQ decline from ages 12–18, while another indicated that cigarette smoking may be a confounder (Mokrysz et al., 2016; Meier et al., 2018). Familial factors may also contribute to the observed differences in IQ decline between cannabis users and non-users. In a large longitudinal twin-pair study of participants aged 9–11 at baseline, cannabis-using twins did not exhibit greater IQ decline relative to their non-user co-twin when assessed at 18–20 years of age (Jackson et al., 2016). However, a neuroimaging study by Camchong et al. (2017) converges on the former findings with adolescents with cannabis use disorder (CUD) showing decreased caudal ACC RSFC with the left DLPFC and OFC, as well as lower IQ and slower cognitive function across an 18-month follow-up period. Adolescent cannabis use is not consistently associated with deficits in IQ, which may be explained by familial factors and the use of other drugs; therefore, the extent to which these factors interact with the effect of cannabis on the adolescent brain should be considered for other behavioral and neurobiological domains.

Greater amounts of cannabis use have also been prospectively associated with declines in immediate, but not delayed, memory performance (Duperrouzel et al., 2019) and persistent verbal learning impairments (Becker et al., 2015). The latter study also investigated the association between cannabis use and white matter microstructure and found that adolescent cannabis users aged 18–20 at baseline exhibited attenuated FA growth in the superior longitudinal fasciculus, an association fiber that has been largely implicated in cognitive functions (Becker et al., 2015). Also, male adolescent heavy cannabis users exhibited decreased FA in the left temporal lobe, an area implicated in verbal memory. FA reductions were accompanied by complementary increases in radial diffusivity and trace values, all suggestive of decreased myelination (Ashtari et al., 2009). This study reported minimal baseline differences in FA between cannabis users and controls, suggesting that white matter microstructure differences did not predate cannabis use. Interestingly, attenuated loss of cortical thickness across adolescent development (Epstein and Kumra, 2015) and greater GMV (Orr et al., 2019) have been observed in adolescent cannabis users in several regions of the brain bilaterally, both of which have reciprocal relationships with myelination. Cross-sectional studies have reported that adolescent cannabis users also show impairments in working memory, problem-solving, and planning (Harvey et al., 2007; Medina et al., 2007a; Vo et al., 2014). Compensatory hyper-functioning has been reported by fMRI studies in the brains of adolescent cannabis users during task performance, complementing these behavioral findings. For instance, hyper-activation in the DLPFC and the right basal ganglia (Padula et al., 2007; Jager et al., 2010), as well as failure to reduce activation in the right hippocampus (Jacobsen et al., 2004), have been observed in adolescent cannabis users compared to non-users during working memory tasks. In the former studies, activation differences between groups were present despite adequate performance on the task, suggesting that adolescent cannabis users require more neural recruitment to perform the tasks at a comparable level to non-users.

The age of onset also plays a critical role in the effects of adolescent cannabis use on cognition. For instance, adolescent early-onset cannabis use has been associated with poorer sustained attention, impulse control, and verbal IQ compared to a later onset of use in current adult cannabis users (Pope et al., 2003; Fontes et al., 2011). Females may be more susceptible to the effects of earlier initiation of cannabis use on neurocognitive functions, as female adolescents exhibited more spatial working memory deficits compared to males across a five-year follow-up period from a baseline age of 12 (Noorbakhsh et al., 2020). Interestingly, in a longitudinal study that tracked cannabis use across adolescence and into adulthood, earlier onset of cannabis use was associated with longer reaction times during a working memory task, which was mediated by reduced activity in the posterior parietal cortex compared to late-onset use (Tervo-Clemmens et al., 2018). This suggests that early onset of cannabis use may predispose those who continue to use cannabis into adulthood to executive function impairments. Also, Wilson et al. (2000) reported a smaller percentage of cortical gray matter, and a larger percentage of white matter across the whole brain, in adults who initiated cannabis use before age 17 compared to those who initiated their use later. These differences were most prominent in the frontal lobes. However, in a study of adolescent boys followed prospectively into adulthood, no differences were observed in both cortical and subcortical region morphology between non-users and users across different trajectories of cannabis use ranging across infrequent to chronic use (Meier et al., 2019). The mixed findings could be attributed to the differences in study design, whereby some of the aforementioned studies were retrospective, and are therefore susceptible to recall bias. Therefore, the longevity of the effects of adolescent cannabis use on cognitive functions and their neurobiological correlates need to be further elucidated through currently on-going and future prospective longitudinal studies.

### Psychopathology

#### Schizophrenia and Psychosis

Cannabis use is common among first-episode psychosis patients (Katz et al., 2016; Abdel-Baki et al., 2017), and cannabis use has been hypothesized to be a causal factor in these disorders (Toftdahl et al., 2016). More recent data appears to confirm this positive association between adolescent cannabis use and schizophrenia spectrum disorders (Arseneault et al., 2002; Jones et al., 2018), particularly in that cannabis both hastens the onset and amplifies the severity of schizophrenia (Shahzade et al., 2018). However, Hanna et al. (2016) reported better cognitive function in adolescent cannabis users with schizophrenia/schizoaffective disorders, suggesting a potential protective role of cannabis in psychosis-related cognitive dysfunction. Structural MRI studies are not consistent with a neuroprotective effect and have suggested that processes underlying gray matter and cortical maturation may mediate the association between adolescent cannabis use and risk for schizophrenia. Among adolescents aged 10–21, those with CUD and early-onset schizophrenia exhibited decreased GMV in the left superior parietal cortex compared to controls (Kumra et al., 2012). Greater cannabis consumption across an 18-month follow-up period in adolescents with CUD predicted a greater decrease in the left inferior longitudinal fasciculus (Kumra et al., 2012), a white matter tract that was previously shown to be disrupted in adolescents with schizophrenia (Ashtari et al., 2007). Moreover, gender may interact with structural abnormalities mediating the association between cannabis use and schizophrenia. For instance, male adolescent cannabis users, with a high polygenic risk score for schizophrenia across 108 genetic loci exhibited decreased cortical thickness, which was not observed in low-risk male, or high- and low-risk female participants (French et al., 2015). However, gender differences need to be investigated further as current studies report mixed findings. For instance, in a study of Australian adolescents, girls who started using cannabis before the age of 16 displayed higher levels of introvertive anhedonia, a negative schizotypy, than girls who started using cannabis later in adolescence, whereas this association was not present in boys (Albertella et al., 2017). Also, the causal direction of the relationship between adolescent cannabis use and schizophrenia is called into question as Hiemstra et al. (2018) found stronger evidence for a reverse association, showing that schizophrenia genetic risk was predictive of increased cannabis use from age 16 to 20. This study, combined with those outlined above, suggests that the association between adolescent cannabis use with psychosis, while strong, may not be causal, and further study of the functional contributions of the risk of loci identified in these studies might help to unravel this “chicken-or-egg” problem.

#### Depression and Anxiety

Adolescent cannabis users, particularly females, maybe at a heightened risk for mood disorders. Among Norwegian adolescents aged 13–17, cannabis users reported more anxiety and depressive symptoms compared to non-users. Girls reported slightly more symptoms compared to boys despite the lower prevalence of cannabis use among girls (Kaasbøll et al., 2018). Similarly, more internalizing symptoms in female adolescent cannabis users were associated with larger amygdalar volumes (McQueeny et al., 2011); this association was not observed in male participants. Conversely, other studies have found no association between adolescent cannabis use and differences in amygdala volumes between adolescent cannabis users and non-using controls (Ashtari et al., 2011; Weiland et al., 2015). It is important to note that the number of female participants in the McQueeny et al. (2011) study was small and future studies with more female participants would be needed to confirm these results. Despite limited evidence for differences in amygdalar morphology between adolescent cannabis users and non-users, amygdalar hypersensitivity in response to angry faces has been reported in adolescent cannabis users, which could predispose individuals to future mood disorders (Spechler et al., 2015). However, these results are conflicted by a more recent study that showed no differences in amygdalar responsivity to emotional stimuli in adolescents with CUD (Aloi et al., 2018). Psychiatric comorbidity may have masked any association between CUD symptomology and amygdala responsiveness in the latter study. Furthermore, in adolescent cannabis users, depressive symptoms were positively associated with increased connectivity between the left OFC and left parietal regions, while anxiety symptoms were negatively associated with increased connectivity between bilateral OFC with right occipital and temporal regions (Subramaniam et al., 2018). Similarly, decreased FA and increased radial diffusivity and trace in the thalamic radiation were observed in older adolescents with a history of heavy cannabis use (Ashtari et al., 2009); decreased FA in the thalamic radiation has also been previously shown in young adult patients with depression (Lai and Wu, 2014). Also, smaller global white matter volumes were associated with more depressive symptoms in adolescent cannabis users (Medina et al., 2007b), suggesting that white matter abnormalities may extend beyond what is observed at a microstructure level.

A recent meta-analysis of longitudinal studies indicated that adolescent cannabis use is associated with a modest risk of developing depression in young adulthood (Gobbi et al., 2019). A recent population-based cohort of young adults who were retrospectively assessed for adolescent cannabis use and followed over 30 years has also captured this. Adolescent cannabis use, particularly an earlier onset of use, as well as more frequent use was associated with adult depression, independent of adult cannabis and other substance use (Hengartner et al., 2020). Adolescent cannabis use may further exacerbate depressive symptoms in males with mild depression at baseline, with limited evidence to support the self-medication hypothesis, whereby depressive symptoms predicted only slight increases in later cannabis use (Womack et al., 2016). Similarly, anxiety symptoms do not appear to predate adolescent cannabis use and may instead depend on the frequency of use. In a recent longitudinal study, adolescents with higher levels of cannabis use reported more persisting anxiety over the next year compared to less frequent users; anxiety levels at baseline did not predict differences in cannabis use between the groups (Duperrouzel et al., 2018). In longitudinal studies, gender differences in the relationship between adolescent cannabis use and anxiety/depressive symptoms have shown an opposite trend to those reported by cross-sectional studies discussed above. In a large adolescent sample balanced for gender, baseline cannabis use at age 16 predicted increases in depressive symptoms in over three years among male, but not, female African American adolescents (Assari et al., 2018). Another study found an association between escalating cannabis use and decreased connectivity between nucleus accumbens and medial PFC that predicted higher levels of depressive symptoms (Lichenstein et al., 2017). Decreased growth in FA in the right anterior thalamic radiation was also observed over three years in adolescent cannabis users (Becker et al., 2015), suggesting possible shared pathophysiology with young adult patients with depression (Lai and Wu, 2014). Overall, both imaging and behavioral findings support a strong relationship between adolescent cannabis use and mood disorders that appear to uniquely interact with gender; neural markers that may give rise to these differences between males and females should be investigated in future studies.

### Future Substance Use

In addition to the relationships between adolescent cannabis use and the risk for schizophrenia and mood disorders, longitudinal studies have revealed that occasional and early-onset cannabis use in adolescence predicts nicotine use and dependence, harmful alcohol consumption, and other illicit drug use in adulthood (Degenhardt et al., 2010; Swift et al., 2012; Scholes-Balog et al., 2016; Jin et al., 2017; Taylor et al., 2017; Pampati et al., 2018). When examining the risk of future drug dependence as a consequence of adolescent cannabis use, it may also be important to consider different cannabis use behaviors, such as using cannabis in social settings vs. solitary use. Solitary cannabis use may present as a risk factor for future cannabis dependence as a recent study showed that compared to social-only use, solitary use is associated with greater cannabis use, as well as CUD symptoms in young adulthood (Creswell et al., 2015). However, these results should be interpreted with caution as the association between solitary cannabis use and future CUD symptoms disappeared after controlling for adolescent CUD symptoms. Interestingly, early-onset cannabis use has previously been associated with anti-social behavior, which may, in turn, promote solitary use (Scholes-Balog et al., 2016). Similar findings have been observed for cannabis vaping, which has been relatively under-studied compared to combustible cannabis use and nicotine vaping despite its prevalence. Cassidy et al. (2018) recently observed that youth entering college are more likely to initiate cannabis vaping if they have a prior history of any cannabis or e-cigarette use, and the risk for vaping cannabis scales with the number of peers also engaging in use. However, the frequency and intensity of use among those who initiate cannabis vaping in social settings and the risk for the development of CUD in these populations are not defined, nor is the use of cannabis vaping in younger adolescent populations.

Earlier onset and greater duration of cannabis use were also associated with risky and impulsive decision-making in adolescent users (Solowij et al., 2012), and impaired decision-making, in turn, may promote substance use. Neuroimaging studies suggest functional compensation as De Bellis et al. (2013) reported that adolescents with CUD exhibit higher activity in the left superior parietal lobule, left lateral occipital cortex, and bilateral precuneus during risky decision-making despite no group differences in performance. Despite cross-sectional associations between cannabis use and poor decision-making in youth aged 14–17 at baseline, cannabis use was not associated with changes in decision-making over a one-year follow-up period (Duperrouzel et al., 2019). This suggests that impaired decision-making may predate cannabis use initiation in adolescence. This is in line with a structural MRI study showing that smaller OFC volume, implicated in decision-making, predicted the initiation of cannabis use by the age of 16 (Cheetham et al., 2012). Adolescent cannabis users also exhibited diminished ability to disengage motivational circuitry during non-rewarding events in the monetary incentive delay task despite normal performance as evidenced by heightened striatal activity in cannabis users compared to non-users, which could drive risk-seeking behavior even in the face of negative outcomes (Jager et al., 2013). Additionally, adolescents with CUD exhibited greater accuracy across trials in the monetary delay task, and greater functional global connectivity across networks that included mesocorticolimbic nodes during monetary reward anticipation (Nestor et al., 2019). The group also showed enhanced integration, defined as higher information exchange between regions and a greater number of connections to the nearest nodes, alluding to neural refinement deficiencies. Superior performance may be mediated by higher motivation as there were no group differences in performance and global connectivity within different trials; however, an earlier study showed reduced motivation in adolescent heavy cannabis users, which may instead indicate lack of motivation at greater consumption levels, potentially failing to seek treatment (Lane et al., 2005). In another study, adolescent cannabis users exhibited an enhanced neural response to both wins and losses, the latter suggesting greater sensitivity during negative feedback (Acheson et al., 2015). In contrast to results from Nestor et al. (2019), Acheson et al. (2015) showed that despite seeing no differences in global connectivity, analyses of the individual paths revealed that adolescent cannabis users differed in connectivity from controls in one-third of the total paths analyzed in response to losses, but no individual path differences were observed during wins. Although these results confirm differences in sensitivity to negative feedback observed in previous studies, it differentially highlights the importance of assessing connectivity within individual networks when investigating alterations in reward circuitry. Both behavioral and neural findings indicate that adolescent cannabis use may increase the risk for future substance use and associated behaviors; however, further research is needed to assess the effects of vaping and different cannabis use behaviors (e.g., solitary vs. social) on this relationship.

## Opioids

In 2017, an estimated 3.1% of adolescents aged 12–17 had misused opioids in the past year (Figure 1A; Substance Use and Mental Health Services Administration, 2018). While these numbers are lower than the prevalence for other substances, the alarming trends of problematic opioid use in North America, the high mortality associated with opioid use, and their exclusion from previous reviews on this topic necessitate further attention. Opioids produce their effects by modulating the excitability of neurons *via* mu, kappa, and delta-opioid receptors as well as nociception receptors. Little is known about opioid receptor development and the consequences of opioid use during adolescence; however, the endogenous opioid system has been observed to change throughout adolescent development, highlighting the necessity for future research during this vulnerable window (Thorpe et al., 2020). Due to fewer findings, this section is considerably narrower in its scope compared to the previously reviewed substances (Supplementary Table S4).

### Cognition

Very little clinical work has been conducted on the long-term effects of opioids on memory and cognition. Given that adolescent opioid use is rarely unaccompanied by other substance use, it is difficult to attribute any effects to opioids on their own. One study found that opioid-dependent adolescents had significantly impaired working memory, but was unable to determine if these deficits were substance-induced or pre-existing before use (Vo et al., 2014). However, the opioid-using group had similar levels of cannabis use as a cannabis-only using group in the same study and the working memory deficits seen were comparable to those of cannabis-only users. Future studies looking into the effects of long-term prescription opioid use in adolescence on cognition are warranted. This would allow for the study of opioids in populations that do not use other substances and give insight into the neurocognitive effects of illicit opioids without the confound of other drugs.

### Psychopathology

Studies conducted on the effects of opioids relating to cognition and psychopathology have shown higher rates of comorbid psychiatric disorders such as MDD, substance use disorder, ADHD, antisocial personality disorder, borderline personality disorder, and post-traumatic stress disorder compared to non-users (Mills et al., 2004; Subramaniam and Stitzer, 2009; Subramaniam et al., 2009; Edlund et al., 2015). Though retrospective, adolescents with MDD and non-medical prescription opioid use often reported MDD to predate opioid use, suggesting MDD to be a risk factor for future opioid abuse (Edlund et al., 2015). In a cross-sectional study of 14- to 18-year-olds, Subramaniam and Stitzer (2009) found that 83% of adolescents with opioid use disorders had a co-occurring psychiatric disorder. Thus, opioid use and several psychopathologies appear to be related but, unfortunately, the directional relationship between opioids and their comorbidities is not known, highlighting the need for future longitudinal studies.

### Future Substance Use

As discussed in previous sections, many substances are associated with an increased risk of future substance use. Opioids are likely not an exception to this trend, and it is thus alarming that they are both regularly prescribed to adolescents and often available in lower doses in over-the-counter products such as acetaminophen and cough syrups (Van Hout and Norman, 2016). Indeed, one study showed that students in grade 12 who had ever used prescription opioids were 33% more likely to misuse opioids by the age of 23, independent of their cannabis, cigarette, and alcohol use (Miech et al., 2015). Additionally, adolescents that misuse prescription opioids were more likely to initiate heroin use, with a younger age of initiation of non-medical prescription opioid use being strongly associated with the subsequent development of opioid use disorder (Cerdá et al., 2015; Schepis and Hakes, 2017). Given the potential for prescription opioid use to increase susceptibility to opioid misuse, it is important that health professionals carefully weigh the benefits and potential detriments that opioids might have on adolescent neurodevelopment when deciding on treatment options.

## Co-Use

The co-use of substances is common among adolescents. The National Longitudinal Study of Adolescent to Adult Health found that nearly one in five adolescents report using cigarettes, alcohol, and cannabis, either individually or in combination before the age of 16 (Moss et al., 2014). For clarity, we define co-use as either concurrent, in which multiple substances are used on different occasions, or simultaneous, in which substances are used on the same occasion. During adolescence, it was more common to have used cigarettes, alcohol, and cannabis concurrently than it was to have only used one of the substances individually. The survey also reported the rates of using alcohol and nicotine as 22%; cannabis and nicotine as 21.6%; and alcohol and cannabis as 34.1%. Nearly all research on adolescent substance use (as well as most reviews on the topic) has focused on individual use, but using multiple substances is more common than individual use. This underscores the need for research into the combined effects of substances on adolescent neurodevelopment. Furthermore, the neural correlates of co-use are especially understudied, highlighting the need for future research in this area. The studies to date investigating the effects of co-use are summarized in Supplementary Table S5.

### Alcohol and Nicotine

Currently, human studies on the neurobiological changes associated with combined alcohol and nicotine use in adolescence do not exist and the same is true of the effects on cognition. However, significantly increased risk for psychopathology and future substance use has been observed.

#### Psychopathology

A study looking at substance use and psychiatric comorbidity in subjects aged 13–15 found that regular alcohol and nicotine use had an additive risk for psychiatric disorders, with especially high risk for depressive disorder (Boys et al., 2003). A 2016 study found that alcohol and cigarette consumption increased physical aggression in adolescents aged 14–16 (Matuszka et al., 2016). This increase was significantly greater than that observed in non-concurrent users, showing greater effects in combination than those of the individual substances.

#### Future Substance Use

Nicotine and alcohol also have additive effects on the risk for future substance use in that concurrent use predicts a greater risk of future substance abuse. A U.S. national survey on alcohol users aged 12–20 found that subjects with a past-year smoking status drank more alcohol on average and had a higher risk for AUD than those that drank equal amounts without smoking (Grucza and Bierut, 2006). These results were the strongest in younger participants. In line with these findings, a longitudinal study found similar results, showing that by age 15, alcohol users that smoked tobacco consumed more alcohol and cannabis (Schmid et al., 2007).

### Cannabis and Nicotine

As with combined alcohol and nicotine use, no studies addressing the effects of combined cannabis and nicotine during adolescence on cognition exist. However, some evidence points to increased risk of psychiatric disorders and increased substance use following combined cannabis and nicotine consumption.

#### Psychopathology

A cross-sectional study looking at combined substance use and psychiatric morbidity in adolescents aged 13–15 found that regular cannabis and nicotine use had an additive risk for psychiatric disorders (Boys et al., 2003). This risk was especially high for the development of depressive disorders and was increased further with the addition of regular alcohol consumption. Longitudinal studies on the effects of combined substance use on psychiatric morbidity are warranted to understand the directionality of this relationship.

#### Future Substance Use

The combined use of cannabis and nicotine has also been associated with increased substance use. In a cross-sectional study of cigarette smoking 13–17-year-olds, the frequency of cannabis use was associated with increased measures of nicotine addiction (Rubinstein et al., 2014). A cross-sectional fMRI study by Karoly et al. (2015) found that adolescents that used tobacco alone had decreased BOLD response in the nucleus accumbens during a monetary incentive delay task compared to non-using peers. However, these differences were not seen in those using both tobacco and cannabis. Cannabis may be counteracting the effects of tobacco on the nucleus accumbens, possibly explaining why the frequency of cannabis use is associated with increased measures of nicotine addiction in these populations; however, results from longitudinal studies investigating this relationship are required before any hypotheses can be made with confidence.

### Cannabis and Alcohol

#### Cognition

The effect of cannabis and alcohol co-use on cognition seems to largely depend on the cognitive behavior being measured. In a longitudinal population-based analysis of grade 7 students, Morin et al. (2019) found that among co-users, cannabis, but not alcohol, was associated with short-term neurotoxic effects on working memory and inhibitory control as well as long-term effects on perceptual reasoning and delayed memory recall. In another study, hangover symptoms among adolescent heavy drinkers were associated with worse verbal learning and memory but these deficits were not seen in adolescents with similar alcohol consumption and heavy cannabis use (Mahmood et al., 2010). The finding that cannabis may provide some neuroprotective effects against heavy alcohol use is also supported by some imaging studies. Alcohol and cannabis appear to have opposing effects on cortical thickness; among co-users, lifetime cannabis use is associated with decreased cortical thickness, while lifetime alcohol use is associated with increased cortical thickness (Jacobus et al., 2014, 2015). Co-users have also shown differential white matter changes associated with cognition, suggesting a possible neuroadaptation resulting in additive and subtractive responses to substance use (Bava et al., 2010). Other studies have also found these subtractive effects, with alcohol alone affecting white matter integrity, but not both alcohol and cannabis; this further suggests possible neuroprotective effects of cannabis when combined with alcohol (Jacobus et al., 2009; Bava et al., 2013; Infante et al., 2018). However, a longitudinal study that compared users at baseline to their three-year follow-up found similar decreases in white matter integrity for both alcohol and co-users (Jacobus et al., 2013a).

In some psychosocial and cognitive domains, co-use appears to have additive deficits. Co-users are more likely to drive intoxicated (Shillington and Clapp, 2001; Terry-McElrath et al., 2014) and have legal problems (Shillington and Clapp, 2001; Green et al., 2016) than those that use each substance individually, suggesting that co-use may play a role in processes, such as inhibitory control (Galambos et al., 2005). A study by Winward et al. (2014) also found that adolescent users of both substances performed worse on a working memory task. Some neuroimaging studies support these results. A study that compared white matter integrity pre- and post-substance use initiation found that initiation of combined alcohol and cannabis use was associated with decreased white matter integrity in most tracts, including the corpus callosum, corticospinal tract, occipital fasciculus, forceps major, internal capsule, and corona radiata, while the initiation of alcohol-only was not linked to changes in white matter integrity (Jacobus et al., 2013b). Interestingly, in most regions at the baseline time point, youth who would later initiate both alcohol and cannabis use demonstrated FA greater than or equal to youth that initiated alcohol use only. This pre-existing increased white matter integrity could explain the supposed neuroprotective effects of cannabis suggested in other studies (Jacobus et al., 2009; Bava et al., 2013). A later study by the same group also found that alcohol-only initiators and controls have greater cortical thickness before initiation compared to those that initiated both cannabis and alcohol, further suggesting that some neurophysiological differences in these groups precede substance use (Jacobus et al., 2016). Functional MRI studies in co-users have shown dysfunction in frontal and temporal regions, and a decoupled association between hippocampal symmetry and verbal learning (Schweinsburg et al., 2005, 2011; Medina et al., 2007b). An fMRI study found decreased BOLD response in the thalamus, insula, and striatum versus non-users when taking risks (Claus et al., 2018). A cross-sectional DTI study by Bava et al. (2009) showed altered frontoparietal networks and fiber projections within circuits responsible for the modulation of complex sensory, motor, and cognitive processing, namely in fibers of the postcentral gyrus, splenium of the corpus callosum, inferior frontal region, and left superior longitudinal fasciculus. With some studies suggesting cannabis to be neuroprotective, some findings appearing to be the result of an individual substance, and others suggesting co-use to have additive deficits, it is difficult to make any clear conclusions. These conflicting findings are likely the result of significant methodological differences and the potential for different use cases to result in distinct findings (e.g., binge drinking vs. heavy drinking vs. AUD or simultaneous use vs. concurrent use). Thus, further studies are required to make sense of the complicated relationship between alcohol and cannabis co-use during adolescence.

### Psychopathology

Compared to alcohol or cannabis use alone, adolescent use of both substances is associated with an increased likelihood of a depressive disorder (Boys et al., 2003). This relationship is also supported by DTI studies; adolescent co-use was associated with decreased FA in the inferior frontal region and left superior longitudinal fasciculus, regions that are similarly altered in adolescent depression (Bava et al., 2009; Cullen et al., 2010).

#### Future Substance Use

Co-use of cannabis and alcohol appears to potentiate future substance use. Co-users consumed more illicit drugs (Magill et al., 2009; Green et al., 2016; Hayaki et al., 2016; Patrick et al., 2018) than those that used alcohol only. There is also evidence that the simultaneous use of alcohol and cannabis together have greater effects on risk for future substance use-related problems than concurrent use (Brière et al., 2011). Similarly, simultaneous users show increased use of illicit drugs compared with those who concurrently use both substances (Patrick et al., 2018). Unfortunately, without longitudinal studies following adolescents before substance use initiation, it is difficult to infer the directionality of these relationships; therefore, interpretations of these results should be cautiously done.

## Limitations

Studies investigating drug-associated alterations to adolescent neurodevelopment have several limitations. Foremost, it is necessary to highlight the difficulty of recruiting younger participants due to issues surrounding parental consent, which may hinder researchers’ ability to match key variables between users and non-user controls, such as age, use of other substances, and underlying psychiatric comorbidities. Emerging evidence also underscores the importance of matching study participants based on genetic variation, as genetic variation in a variety of genes have been associated with increased risk for substance use and associated behaviors, and mediate the effects of adolescent substance use (Hines et al., 2015; Patriquin et al., 2015). Controlling for the use of other substances is also of importance as the contribution of multiple drugs to the observed neurobiological and behavioral differences are difficult to disentangle. Also, to control for the acute effects of substance use, a criterion of abstinence is put in place in many studies; however, withdrawal symptoms may confound the results of studies that employ such a design. Abstinence may also be self-reported by participants in place of objective measures, such as urine analysis, making it difficult to isolate acute drug effects from those that are long-lasting. Furthermore, this review is limited in its scope to the potential effects of nicotine, alcohol, and cannabis given their use prevalence during adolescence and, in the case of opioids, the emerging nature of the opioid epidemic. However, adolescents are known to consume a wide variety of other, albeit relatively under-investigated, drugs such as cocaine, ecstasy, and inhalants (Johnston et al., 2020).

Furthermore, the cross-sectional design of many studies reviewed here limits conclusions on causal directions as there is a possibility that observed neuroimaging and behavioral differences predate the onset of substance use. While we addressed studies that highlighted neurobiological or cognitive factors antedate to substance use here, studies that did not account for underlying between-subject differences may contribute to discrepant findings. In the absence of controlled trials, longitudinal studies are more useful in inferring directional relationships between drug use and neurobiological consequences, especially when baseline measurements are carried out before the onset of substance use. Therefore, more longitudinal analyses, especially studies that are concerned with structural and functional differences within the brain, are needed.

## Future Directions

Future prospective longitudinal studies (e.g., the on-going Adolescent Brain Cognitive Development Study of the National Institute on Drug Abuse) looking at the markers of neurobiological function (e.g., brain imaging) before the appearance of substance use could help uncover the mechanistic underpinnings of the long-term consequences of substance use that have been reviewed here. Importantly, as studies indicate compounding detrimental effects of adolescent and prenatal drug exposure on neurological and cognitive outcomes (Jacobsen et al., 2007b,c), not all studies outlined here control for prenatal drug exposure. Future studies would benefit from investigating the impacts of drug exposure at multiple developmental points and how this compares with adolescent-exclusive use.

The neurobiological and cognitive consequences of vaping should also be the target of future studies, as the persisting effects of adolescents using electronic drug delivery devices relative to traditional (i.e., combustible) delivery methods are largely unknown outside of future drug use susceptibility. To date, only one neuroimaging study investigating young adult e-cigarette experimenters and those at risk to try e-cigarettes exists in the literature (Garrison et al., 2018), though the mean age of participants in this study was above our 19-year-old cutoff age. Another study (Chen et al., 2018) using a group of participants with a mean age within our cutoff also investigated neural activity, however, it was in in response to e-cigarette advertisements, and the participants in this study were selected based on combustible smoking status rather than e-cigarette use. Even fewer studies have been conducted on the outcomes of cannabis vaping during adolescence despite increasing trends of vaping cannabis, as well as edible use, both of which have been associated with heavier cannabis use (Patrick et al., 2020). Neurobiological investigations of cannabis and nicotine vaping susceptibility and potential for harm, especially surrounding the transition to combustibles, in this at-risk population, must, therefore, become a priority for future studies.

Moreover, through the reverse translation of findings from clinical populations, the causal underpinnings of the consequences of adolescent substance use can be uncovered. Related to the emerging trends such as the increases in vaping, the availability of animal models of self-administration using electronic devices, combined with pre-clinical neuroimaging methods, will help establish the direct causal consequences of adolescent vaping (Hines et al., 2015; Freels et al., 2020). Lastly, while our review did not address specific therapeutic attempts to reverse the effects of adolescent drug use, future studies can begin to target these changes toward the development of strategies that help to reduce or prevent some of the deleterious effects of adolescent substance exposure, especially if these interventions can be targeted for use in adolescence.

## Conclusion

Despite the overall recent downward trends in adolescent substance use the prevalence of adolescent substance use remains a significant public health concern, largely due to the consequences of this use and the especially vulnerable window of neurodevelopment during this period. In this review, we highlighted the neurobiological and behavioral changes that arise from adolescent nicotine, alcohol, cannabis, and opioid use or their combination. Specifically, adolescent drug exposure may contribute to increased risk for the development of cognitive deficits, psychopathology, or subsequent substance use disorders that may be related to the structural and functional changes in the brain. Investigating mechanisms underlying these alterations may provide novel avenues for the development of therapeutics that target these mechanisms to prevent and reduce the harm associated with substance use in adolescence.

## Author Contributions

SH drafted the cannabis and alcohol literature review and the “Limitations” section. Besides, SH was responsible for consolidating the review, formatting Supplementary Tables S1–S5, and the formatting of the review. HT drafted the nicotine literature review, the “Future Directions” section, and Figure 1, as well as helped format Supplementary Tables S1–S5. JF drafted the opioid and the co-use literature review and the “Introduction” section. RM contributed to the literature review and Figure 1. JK formulated the idea for the review and guided the research and writing process. All authors contributed to the article and approved the submitted version.

## Conflict of Interest

The authors declare that the research was conducted in the absence of any commercial or financial relationships that could be construed as a potential conflict of interest.

## Abbreviations

ACC: Anterior Cingulate Cortex
ADHD: Attention Deficit Hyperactivity Disorder
AUD: Alcohol Use Dependence
BOLD: Blood Oxygenation Level-Dependent
CUD: Cannabis Use Dependence
DLPFC: Dorsolateral Prefrontal Cortex
DTI: Diffusion Tensor Imaging
EEG: Electroencephalography
FA: Fractional Anisotropy
fMRI: Functional Magnetic Resonance Imaging
GMV: Gray Matter Volume
IQ: Intelligence Quotient
MDD: Major Depressive Disorder
MRI: Magnetic Resonance Imaging
OFC: Orbitofrontal Cortex
PFC: Prefrontal Cortex
RSFC: Resting-State Functional Connectivity.
