# Supplementary material for: Adolescent Substance Use and the Brain: Behavioral, Cognitive and Neuroimaging Correlates
Source: Front Hum Neurosci. 2020 Aug 4;14:298. doi: 10.3389/fnhum.2020.00298 (PMC7418456; doi:10.3389/fnhum.2020.00298)
Supplement: Supplementary file 1 [file Table_1.docx]

Table 1A: Summaries of cross-sectional, retrospective, and longitudinal studies assessing the potential cognitive, behavioral, psychopathological, and future substance use vulnerability outcomes of use of tobacco products by adolescents.

^E^ = E-cigarette study; * = estimation; (↑) = increase; (↓) = decrease; (NC) = no change; (+) = positive association; (-) = negative association; (≠) = no association; male = ♂; ♀= female; ⚧= transgender; M_age_ = mean age.

| Authors | Study Type | N | Age | Gender % (♂/♀/⚧) | Findings |
| --- | --- | --- | --- | --- | --- |
| **Cognition** | | | | | |
| Treur et al. (2015) | Cross-sectional; Longitudinal | Adults: 20,824  Adolescents: 11,386 | Adult: 18-97 (M_age_: 42; monozygotic twin M_age_: 34)  Adolescent: 8-18 (M_age_: 15; monozygotic twin M_age_: 15 (baseline), 17 (follow-up)) | Adult monozygotic twins: 26/74  Adolescent monozygotic twins: 32/68 | Smoking ↑ attentional problems. Age of onset (-) attentional problems. |
| Tercyak et al. (2002) | Cross-sectional | 1,066 | 14-17* | 48/52 | Inattention or hyperactivity/impulsivity (+) ever-smoking. Inattention ↑ current smoking likelihood. |
| Jacobsen et al. (2005) | Cross-sectional | 53 | 14-18 (Smoker M_age_: 17.0; Non-smoker M_age_: 16.6) | Smokers: 34/66  Non-smokers: 38/62 | Acute abstinence ↑ withdrawal symptoms, depressed mood, anxiety, working memory, & verbal memory performance deficits. Smokers ↓ working memory & divided attention response performance. Age of smoking onset (+) cognitive performance. |
| McNeill et al. (1986) | Cross-sectional | 597 | 11-17 | 0/100 | Withdrawal effects (+) cotinine levels, depth of inhalation, & weekly cigarette consumption. Abstinence ↑ moodiness, restlessness, & hunger. |
| Rojas et al. (1998) | Cross-sectional | 249 | M_age_: 16* | 57/43 | Smoking level ♀<♂. Cravings (+) cotinine levels, depression (♂<♀), dependence (♀<♂) symptoms. Dependence, body weight change during adolescent cessation < adults. |
| DiFranza et al. (2007) | Longitudinal | Baseline: 1,246  Follow-up: 970 | 11-14 (M_age_: 12.2) | 48/52 | Relaxation after first inhale, depressed mood ↑ dependence, losing smoking autonomy. Extracurricular activities, ♀, & attention disorder symptoms ↓ dependence & losing autonomy. |
| Leventhal et al. (2015)^E^ | Longitudinal | 2,530 | 13-15  -Baseline: 13-14* (E-cigarette ever-user M_age_: 14.10; E-cigarette never-user M_age_: 14.05)  -Follow-up: 14-15* | 47/53 | Cigarette use (+) parental education, peer smoking, impulsivity, other product use, delinquency, & smoking expectancies. Baseline e-cigarette use ↑ future combustible tobacco product use. Combustible tobacco product use ↑ follow-up e-cigarettes use. |
| Yin et al. (2016) | Cross-sectional | 36 | 15-18 (Smoker M_age_: 16.9; Non-smoker M_age_: 17.3) | 100/0 | Smokers ↑ No-Go trial response errors. Smokers ↓ inhibition control processing in No-Go trials. |
| Weiser et al. (2010) | Longitudinal | 20,221 | 18-21  -Baseline: 18  -Follow-up: 18-21 | 100/0 | Cognitive test scores in current smokers < former smokers < non-smokers. Daily cigarette consumption (-) cognitive scores. |
| **Psychopathology** | | | | | |
| Zammit et al. (2003) | Longitudinal | 50,053 | 18-47*  -Baseline: 18-20  -Follow-up: 18-47* | 100/0 | Smoking by age 18 (≠) schizophrenia development within five years. Medium/heavy smokers ↓ schizophrenia risk five+ years post-conscription. Smoking (+) psychotic illness diagnoses. |
| Weiser et al. (2004) | Longitudinal | 14,248 | 18-34  -Baseline: 18  -Follow-up: 18-34 | 100/0 | Smoking & smoking heaviness (+) future hospitalization for schizophrenia. |
| McGrath et al. (2016) | Longitudinal; Retrospective | Baseline: 7,223  Follow-up: 3,801 | 0-21 | 48/52 | Age of smoking onset (-) delusions, hallucinations, & psychotic disorder diagnoses. Hallucinations (-) age of smoking onset. Non-affective psychosis (≠) age of smoking onset. |
| Mustonen et al. (2018) | Longitudinal; Retrospective | 6,081 | 15-30  -Baseline: 15-16  -Follow-up: 15-30 | 48/52 | Adolescent smoking & psychosis (+) drug use, parental drug use, parental psychosis. Heavy smoking ↑ psychosis risk. Age of onset (-) future psychosis risk. |
| Jones et al. (2018) | Longitudinal | 5,300 | 12-20 | 44/56 | Smoking (≠) psychotic experiences at age 18. Early psychotic experiences (+) risk for later cigarette use. |
| Wu & Anthony (1999) | Longitudinal | 1,731 | 8-14  -Baseline: 8-9  -Follow-up: 13-14 | 50/50* | Smoking (+) first occurrence of depressed mood risk. Depressed mood (≠) smoking onset. |
| Goodman & Capitman (2000) | Longitudinal | Non-smokers: 6,947  Non-depressed: 8,704 | 11.43-21.23 (Non-smoker M_age_: 15.32; Non-depressed M_age_: 15.48) | Non-smokers: 51/49  Non-depressed: 52/48 | Baseline non-depressed adolescent smokers ↑ follow-up high depressive symptoms. Moderate/heavy smoking black < white adolescents. |
| Albers & Biener (2002) | Longitudinal | 522 | 12-19  -Baseline: 12-15 (M_age_: 13.49)  -Follow-up: 16-19 | 50/50 | Baseline smokers ↑ follow-up depressive symptoms. |
| Needham (2007) | Longitudinal | 10,828 | 12-28  -Baseline: 12-18 (M_age_: 15.28)  -Follow-up: 13-28 | 47/53 | Smoking (+) depressive symptoms. Smoking progression (+) depressive symptom changes (♀ only). Baseline smoking frequency (+) depressive symptom decline rate. |
| Ilomäki et al. (2008) | Cross-sectional; Retrospective | 508 | 12-17 (♂ M_age_: 15.4; ♀ M_age_: 15.5) | 41/59 | Depressive disorder (+) daily smoking (♀<♂). Smoking (+) conduct & oppositional defiant disorders. Age of smoking onset in ♀ with substance use disorder < ♂ with substance use disorder < those without substance use disorder. |
| Audrain-McGovern et al. (2009) | Longitudinal | 1,093 | 14-18  -Baseline: 14*  -Follow-up: 15-18* | 47/53* | Baseline depression & peer smoking ↑ follow-up smoking & smoking progression.  Smoking ↑ deceleration of depression symptoms. |
| Morrell et al. (2010) | Cross-sectional | 1,214 | M_age_: 18 | 40/60 | Depression & anhedonia ↑ smoking negative reinforcement expectancies. Depression or anhedonia ↑ smoking likelihood in (♀ only). |
| Slomp et al. (2019) | Cross-sectional | 988 | 11-17 (M_age_: 13.5) | 56/44 | Smokers ↑ suicide ideation (♂<♀), anxiety & depression symptoms. |
| Brown et al. (1996) | Cross-sectional; Longitudinal; Retrospective | Baseline: 1,709  13-month follow-up: 1,507 | 14-18 (M_age_: 16.6 (baseline)) | 48/52 (baseline) | Age of onset (+) future drug abuse. Smoking ↑ MDD risk. |
| Stein et al. (1996) | Longitudinal | 461 | 12-27*  -Baseline: 12-15*  -Follow-up: 16-27* | 29/71 | Middle adolescent & older smoking (+) depressed mood. Early adolescent smoking (+) sociability, cheerfulness, & extroversion. |
| Choi et al. (1997) | Longitudinal | 792 | 12-22  -Baseline: 12-18  -Follow-up: 15-22 | 37/63 | Baseline current smoking ↑ depressive symptoms risk. Baseline experimental smoking ↑ depression symptom risk (♀ only). |
| Windle & Windle (2001) | Longitudinal | 1,218 | 15-18*  -Baseline: 15-17* (M_age_: 15.54)  -Follow-up: 16-18* | 52/48 | Heavy baseline smoking (+) follow-up depressive symptoms. High depression symptomology (+) follow-up smoking. |
| Brook et al. (2002) | Longitudinal | 736 | 14-27  -Baseline: 14 (M_age_: 14.05)  -Follow-up: 16-27 | 50/50 | Early/late adolescent smoking ↑ adulthood MDD risk. Early adulthood smoking (NC) MDD risk. |
| Brook et al. (2004) | Longitudinal | 688 | 14-27  -Baseline: 14  -Follow-up: 17-27 | 49/51 | Adolescence/young adulthood smoking ↓ follow-up depressive symptom number. |
| Galambos et al. (2004) | Longitudinal | 1,322 | 12-19  -Baseline: 12-19  -Follow-up: 16-23 | 49/51 | Earlier smoking ↑ future depression symptom risk. Earlier depression symptoms emergence ↑ future high frequency smoking. Smoking level (+) depressive symptom development. |
| Duncan & Rees (2005) | Longitudinal | 13,068 | 13-22  -Baseline: 11-21 (♂ M_age_: 15.62; ♀ M_age_: 15.46)  -Follow-up: 12-22 (♂ M_age_: 16.53; ♀ M_age_: 16.37) | 52/48 | Baseline smoking (+) baseline depression, follow-up depression symptoms. |
| Rodriguez et al. (2005) | Longitudinal | 925 | 14-18  -Baseline: 14  -Follow-up: 15-18 | 46/54 | Baseline high depression, smoking (+) decelerated depression symptom development. Baseline moderate depression, smoking (+) accelerated depression symptom development. |
| Boden et al. (2010) | Longitudinal | 1,265 | 17-25  -Baseline: 17-18  -Follow-up: 20-25 | 50/50 | Nicotine dependence (+) depressive symptoms. Depressive symptoms (≠) nicotine dependence. |
| Moon et al. (2010) | Longitudinal | 5,625 | 12-19  (M_age_: 16.04)  -Baseline: 12-18  -Follow-up: 13-19 | 49/51 | Baseline depressive symptoms (+) future smoking. Smoking (+) follow-up depressive symptoms. |
| Jamal et al. (2011) | Cross-sectional; Retrospective | 1,055 | 18-65 (Early onset smoker M_age_: 42.0; Late onset smoker M_age_: 47.1) | 33/67 | Late-onset smokers risk of early depression/anxiety diagnosis < early-onset smokers. Risk of early depression/anxiety diagnosis ♂ smokers < ♀ smokers. |
| Beal et al. (2014) | Longitudinal | 262 | 11-20  -Baseline: 11-17  -Follow-up: 15-20 | 0/100 | Baseline smoking ↑ future depression symptom risk. Depression symptoms (≠) future smoking. |
| Gage et al. (2015) | Longitudinal | 1,791 | 16-18  -Baseline: 16  -Follow-up: 18 | 42/58 | Smoking (≠) future depression, anxiety. |
| Wang et al. (1996) | Longitudinal | 3,811 | 12-21  -Baseline: 12-18  -Follow-up: 15-21 (M_age_: 16.5) | N/A | Smoking (+) depression. Depression (+) smoking. |
| Repetto et al. (2005) | Longitudinal | 623 | 14-23  -Baseline: 14-17 (M_age_: 14.55)  -Follow-up: 15-23 | 49/51 | Smoking trajectory (≠) future depressive symptoms. Depression symptoms change (+) smoking trajectory. |
| Clark et al. (2007) | Longitudinal | 1,513 | 11-16  -Baseline: 11-14  -Follow-up: 13-16 | 47/53 | Baseline smoking (≠) follow-up depressive symptom risk. |
| Munafò et al. (2008) | Longitudinal | 13,405 | 13-19  -Baseline: 13-18 (M_age_: 15)  -Follow-up: 14-19 | 49/51 | Baseline depression (+) follow-up smoking (not in baseline regular smokers). Baseline smoking (≠) follow-up depression. |
| Hu et al. (2011) | Longitudinal | 660 | 11-17  -Baseline: 11-17 (M_age_: 14.7)  -Follow-up: 13-19 | 46/54 | Baseline dependence, positive smoking experiences, perceptions of peer smoking, conduct problems (+) follow-up dependence. Baseline depression (≠) future nicotine dependence. Baseline depression (≠) future depression. |
| Strong et al. (2014) | Longitudinal | 703 | 15-43  -Baseline: 15-16  -Follow-up: 32-43 | 49/51 | Baseline smoking (≠) adulthood MDD. |
| **Future Substance Use** | | | | | |
| Stanton (1995) | Longitudinal | 1,139 | 15-18  -Baseline: 15  -Follow-up: 18 | 51/49* | Smoking at age 15 (+) smoking at age 18. Daily smoking at age 15, smoking to relieve withdrawal, & appetite & weight changes ♂<♀. |
| Everett et al. (1999) | Cross-sectional; Retrospective | 13,858 | 16-18* | N/A | Smoking onset (<15-years-old) ♀ < ♂. Ever-smoking & younger age of onset in black < white & Hispanic adolescents. Age of onset (-) smoking frequency. |
| Colder et al. (2001) | Longitudinal; Retrospective | 323 | 11-16  -Baseline: 11-12 (M_age_: 12)  -Follow-up: 12-16 | 48/52 | Five adolescent smoking trajectories: 1) stable light smoking, 2) stable smoking, 3) early rapid escalation, 4) late moderate escalation, & 5) late slow escalation. Early onset smoking ↑ future smoking. |
| Riggs et al. (2007) | Longitudinal | 1,017 | 12-28  -Baseline: 12  -Follow-up: 12-28 | 45/55 | Early onset heavy smoking ↑ future smoking dependence. Smoking frequencies diverged at age 15 (occasional vs. late heavy smokers) & 12 (early heavy smokers). |
| Dierker et al. (2012) | Cross-sectional; Retrospective | 10,123 | 13-17 (M_age_: 15.2) | N/A | Age of onset (+) weekly, daily use. Faster smoking escalation (+) dependence. Age of onset, dependence ♂ < ♀. |
| Buchmann et al. (2013) | Longitudinal | 213 | 15-22  -Baseline: 15  -Follow-up: 22 | 47/53 | Age of first use (-) positive smoking experiences. Age of first cigarette, age of pleasurable smoking experiences (-) smoking in young adulthood smoking, smoking frequency, & level of dependence. |
| Taioli & Wynder (1991) | Retrospective | 42,002 | N/A | 72/28 | Age of onset (-) number of cigarettes smoked/day in adulthood. |
| Klein et al., (2013) | Retrospective | 485 | 18-70 (M_age_: 36.4) | 57/43 | Age of onset, purchase of first cigarette, latency between first & second cigarette (-) number of cigarettes smoked per week in adulthood. Age of onset (-) future smoking during illness, smoking to function, perceived benefits of smoking. |
| Lanza & Vasilenko (2015) | Retrospective | 15,748 | 18+ (M_age_: 46.4) | 43/57 | Age of onset ♂ < ♀. Age of onset (-) adult dependence. Rates of adolescent onset-associated adult dependence ♂ < ♀. |
| DiFranza et al. (2000) | Longitudinal | Baseline: 681  Follow-up: 626 | 12-15 (M_age_: 12.6) | 52/48 | Dependence symptoms reported in 62% of adolescents before daily smoking. Dependence symptoms reported in 25% of monthly smokers within two weeks of onset; 22% before end of first smoking month. |
| DiFranza et al. (2002) | Longitudinal | Baseline: 679  Follow-up: 471 | 12-15 (M_age_: 13.1) | 51/49 | Smoking dependence symptoms were reported after first (18%), monthly (33%), weekly (49%), & pre-daily (70%) use. Symptom count, onset ♂ < ♀. Baseline dependence symptoms ↑ follow-up current smoking. |
| Caris et al. (2009) | Cross-sectional | 30,490 | 12-18 | 48/52 | Tobacco use ↑ cannabis use opportunities, cannabis experimentation |
| Lewinsohn et al. (1999) | Longitudinal | 684 | 17-24  -Baseline: 17-18*  -Follow-up: 18-24* | 40/60 | Smoking ↑ future alcohol, cannabis, & other drug disorder risk. Longer duration of smoking cessation following daily smoking ↓ AUD risk. Early smoking onset ↑ future substance use disorder risk. |
| Barrington-Trimis et al. (2016)^E^ | Longitudinal | 298 | 16-19  -Baseline: 16.8-17.9* (Median age: 17.4)  -Follow-up: 18.3-19.0 (Median age: 18.6) | 58/42 | Baseline e-cigarette use ↑ follow-up combustible tobacco product use. |
| Azagba et al. (2017)^E^ | Cross-sectional | 25,637 | 12-19 | 50/50 | E-cigarette experimentation ↓ cigarette smoking susceptibility |
| Miech et al. (2017)^E^ | Longitudinal | 347 | 17-19* (Modal age: 19 (follow-up)) | 44/56 | E-cigarette users ↓ perceived cigarette risk, ↑ follow-up cigarette use. |
| Spindle et al. (2017)^E^ | Longitudinal | 3,757 | 18+ (Baseline M_age_: 18.5) | 44/66 | Baseline e-cigarette use, stress, & impulsivity (+) future cigarette use onset. E-cigarette use (+) ♂, cannabis use. Dual use (+) ♂, other tobacco product use, impulsivity. |
| Wills et al. (2017)^E^ | Longitudinal | 2,338 | 14-17  -Baseline: 14-15 (M_age_: 14.7)  -Follow-up: 15-17 (M_age_: 15.8) | 47/53 | Baseline e-cigarette use (+) follow-up cigarette use risk. Level of e-cigarette use (+) follow-up regular cigarette use. Baseline e-cigarette use (≠) follow-up cigarette use reduction. |
| Barrington-Trimis et al. (2018)^E^ | Longitudinal | 6,258 | 14-18* | 46/54 | Baseline e-cigarette use ↑ combustible smoking onset, frequency of combustible use. Baseline e-cigarette use (NC) follow-up combustible smoking reduction. |
| Berry et al. (2019)^E^ | Longitudinal | 6,123 | 12-18  -Baseline: 12-15 (M_age_: 13.4)  -Follow-up: 15-18 | 51/49 | Baseline e-cigarette, non-cigarette tobacco product use ↑ follow-up combustible cigarette onset, current use risk. Baseline combustible cigarette use ↑ follow-up e-cigarette use risk. |
| Vogel et al. (2019)^E^ | Longitudinal | Baseline: 173 6-month follow-up: 120  12-month follow-up: 127 | 13-19  Baseline: 13-18 (M_age_: 16.6)  Follow-up: 14-19 | 75/25 | Baseline e-cigarette use (+) follow-up nicotine use, dependence, exposure, & the use of combustible cigarettes & high-yield nicotine devices. |
| Vogel et al. (2020)^E^ | Cross-sectional; Longitudinal | 444 | 17-18* (M_age_: 17.48) | 51/49 | E-cigarette dependence < combustible cigarette dependence in dual users. E-cigarette dependence in e-cigarette only users < dual users. Baseline e-cigarette dependence (+) follow-up combustible & e-cigarette use. |
| Cassidy et al. (2018)^E^ | Longitudinal | 1,313 | Baseline M_age_: 18.6 | 45/55 | E-cigarettes ever-use ↑ follow-up cannabis vaping onset. Baseline cannabis & cigarette ever-use ↑ follow-up e-cigarette use. |
| Dai et al. (2018)^E^ | Longitudinal | 10,364 | 12-18  -Baseline: 12-17  -Follow-up: 13-18 | 51/49 | E-cigarette use ↑ follow-up cannabis use likelihood. Young adolescent e-cigarette use (+) future cannabis use. |
| Jackson et al. (2020)^E^ | Cross-sectional | 49 | 16-20 (M_age_: 18.7) | 61/37/2 | Menthol e-cigarette flavor preference < green-apple flavor. Preference for high nicotine concentration e-cigarette < low nicotine concentration. |
| Boykan et al. (2019)^E^ | Cross-sectional | 265 | 12-21 (primarily 15-17) | 34/64/2 | Urinary cotinine levels in weekly < daily smokers. Daily e-cigarette users ↑ pod-based e-cigarette device use. Pod-based e-cigarette users ↑ higher urinary cotinine levels. |
| Goldenson et al. (2017)^E^ | Longitudinal | 181 | 15-17* (M_age_: 16.1) | 53/47 | Baseline nicotine concentration used (+) follow-up smoking & e-cigarette use, frequency of use, intensity of use. |
| Goniewicz et al. (2019)^E^ | Cross-sectional | 22 | 12-21 (M_age_: 16.8) | N/A | Nicotine delivered per puff of older generation e-cigarettes < newer generation e-cigarettes. Urine cotinine levels combustible cigarette users < e-cigarette users (see (Benowitz et al., 2018)). |

Table 1B: Summaries of cross-sectional, retrospective, and longitudinal studies assessing the potential structural, and functional vulnerability outcomes of use of tobacco products by adolescents. ^E^ = E-cigarette study; * = estimation; (↑) = increase; (↓) = decrease; (NC) = no change; (+) = positive association; (-) = negative association; (≠) = no association; male = ♂; female = ♀; M_age_ = mean age.

| Authors | Study Type | N | Age | Gender % (♂/♀) | Findings |
| --- | --- | --- | --- | --- | --- |
| **Structural** | | | | | |
| Akkermans et al. (2017) | Longitudinal | 89 | 9.5-27.6  -Baseline: 9.5-24.2 (Smoker M_age_: 17.9 for smokers; Non-smoker M_age_: 17.6)  -Follow-up: 12.9-27.6 (Smoker M_age_: 21.3; Non-smoker M_age_: 20.9) | -Smokers: 63/36  -Non-smokers: 55/45 | Early onset smoking ↓ frontal cortical thickness. Smoking ↑ inattention. |
| Jacobsen et al. (2007b) | Cross-sectional | 67 | 13-18 (M_age_: 16.5*) | 39/61 | Smoke exposure (+) cortex, corpus callosum, longitudinal fasciculus, internal capsule FA. Auditory attention reaction time (+) internal capsule & temporal lobe FA. |
| Li et al. (2017) | Cross-sectional | 61 | 16-21 (Smoker M_age_: 19.4; Non-smoker M_age_: 19.5) | 100/0 | Smoking ↑ incongruent Stroop task errors. Errors (+) ACC-insula tract FA. Insula-ACC RSFC (-) smoking duration. |
| Li et al. (2015) | Cross-sectional | 49 | 14-23 (Smoker M_age_: 19.7; Non-smoker M_age_: 19.3) | 100/0 | Smoking ↓ frontal cortex cortical thickness. Smoking dependence (-) DLPFC thickness. Lifetime exposure (-) OFC thickness. |
| Chaarani et al. (2019) | Cross-sectional; Longitudinal | GMV analysis: 838  FA analysis: 676  Genetic analysis: 1,417 | -Baseline M_age_: 14.5  -Follow-up M_age_: 16 | GMV and FA analysis:  -Smokers: 50/50 smokers  -Non-smokers: 52/48  Genetic analysis:  -Smokers: 48/52  -Non-smokers: 50/50 | Smoking (-) ventromedial PFC GMV. GMV (≠) future smoking. *CHRNA* polymorphisms ↓ GMV. Smoking (-) corpus callosum FA. |
| van Ewijk et al. (2015) | Cross-sectional | 186 | 14-24 (Regular smoker M_age_: 18.8; Irregular smoker M_age_: 19.1; Non-smoker M_age_: 16.7) | Regular smokers: 78/22  Irregular smokers: 56/44  Non-smokers: 62/38 | Regular smokers ↑ FA, ↓ mean diffusivity in the basal ganglia. Effects of smoking & ADHD on FA showed regional overlap but different directionality. |
| Yu et al. (2016) | Cross-sectional | 45 | 14-23 (Smoker M_age_: 19.6; Non-smoker M_age_: 19.3) | 100/0 | Smokers ↑ FA & axial diffusivity, ↓ radial diffusivity. Corona radiata FA (+) dependence & smoking duration. Corona radiata radial diffusivity (-) smoking duration. |
| **Functional** | | | | | |
| Jacobsen et al. (2007c) | Cross-sectional | 181 | 13-18 (M_age_: 16.6*) | 38/62 | Smokers ↑ depression symptoms, drug use rates, auditory attention brain region activation during task performance. Smokers ↓ intelligence. Smoke exposure (-) auditory lexical discrimination performance. |
| Bi et al. (2017) | Cross-sectional | Functional connect-ivity: 80  Cognitive perform-ance: 56 | 15-24 (Smoker M_age_: 19.62; Non-smoker M_age_: 19.58) | 100/0 | Smokers ↑ incongruent Stroop task errors, ↓ RSFC between anterior insula with PFC, amygdala, & striatum.  Insula connectivity (-) cravings, dependence, Stroop errors committed, & smoking duration. |
| Li et al. (2017) | Cross-sectional | 61 | 16-21 (Smoker M_age_: 19.4; Non-smoker M_age_: 19.5) | 100/0 | Smoking ↑ incongruent Stroop task errors. Errors (+) ACC-insula tract FA. Insula-ACC RSFC (-) smoking duration. |
| Galván et al. (2011) | Cross-sectional | 50 | 15-21 (Smoker M_age_: 19.32; Non-smoker M_age_: 19.00) | 58/42 | Smoking (NC) Stop-Signal task performance. Nicotine dependence (-) response inhibition neural activation. Stop-signal reaction times (-) cortical activity. |
| Lee et al. (2005) | Cross-sectional | 8 | 16-18 (M_age_: 17.13) | 100/0 | 2D smoking cues ↑ PFC, ACC, supplementary motor area, temporal cortex, & occipital lobe activity. 3D virtual reality smoking cues ↑ frontal, occipital, & temporal gyri activation. |
| Rubinstein et al. (2011b) | Cross-sectional | 24 | 13-17 (M_age_: 16.3) | 58/42 | Smokers ↑ smoking cue-induced activation in mesolimbic reward regions, the parahippocampal & occipital gyri, & medial occipital regions. |
| Rubinstein et al. (2011a) | Cross-sectional | 24 | 13-17 (M_age_: 16.3) | 58/42 | Smokers ↓ food cue-induced activation in the insula, putamen, inferior frontal cortex & Rolandic operculum. |
| Dinn et al. (2004) | Cross-sectional | 139 | 17-25 (M_age_: 18.6) | 29/71 | Current smokers (NC) major psychopathology & ADHD symptomology. Smokers ↑ orbitofrontal dysfunction, other drug abuse risk. |
| Jacobsen et al. (2007a) | Cross-sectional | 93 | Smoker M_age_: 16.9  Non-smoker M_age_: 16.6 | 38/62 | Smoking ↓ high-load verbal working memory accuracy. Smoking ↑ activation of memory regions. Abstinence ↓ memory network efficiency. |
| Chen et al. (2018)^E^ | Cross-sectional | 29 | 14-21 (M_age_: 16.96) | 57/43 | E-cigarette advertisements ↑ smoking desire, & activation of cognitive control, reward, visual processing & attention, & memory brain regions. |
| Mashhoon et al. (2018) | Cross-sectional; Retrospective | 30 | 22-40 | 50/50 | Late onset smoker cortical smoking cue reactivity during withdrawal < early onset smoker. Late onset smokers baseline cigarette craving < early onset smokers. |

Table 2A: Summaries of cross-sectional, retrospective, and longitudinal studies assessing the potential cognitive, behavioral, psychopathological, and future substance use vulnerability outcomes of use of alcohol use by adolescents. * = estimation; (↑) = increase; (↓) = decrease; (+) = positive association; (-) = negative association; (≠) = no association; male = ♂; ♀= female; M_age_ = mean age.

| Author | Study Type | N | Age | Gender % (♂/♀) | Findings |
| --- | --- | --- | --- | --- | --- |
| Cognition | | | | | |
| Field et al. (2007) | Cross-sectional | 90 | 16-18 (M_age:_ 16.8) | 92/8 | Heavy drinking (+) inhibitory control deficits & attentional bias for alcohol cues. |
| Melaugh McAteer et al. (2015) | Cross-sectional | 44 | 16-19 (M_age_: 17.1) | 66/34 | Social drinkers (+) attentional bias for alcohol cues. |
| Squeglia et al. (2009) | Longitudinal | 76 | 12-17  -Baseline: 12-14  -Follow-up: 15-17 | 62/38 | More drinking days in the past year ↓ visuospatial function (♀); more hangover symptoms ↓ sustained attention (♂) |
| Ruan et al. (2019) | Longitudinal | 212 | 14-19  -Baseline: 14  -Follow-up: 19 | 57 /43 | Binge-drinking ↑ impulsivity at age 19 & ↓ developmental trajectory of impulsivity. |
| Jones et al. (2017) | Longitudinal | 116 | 10-25  -Baseline: 10-17 (M_age_: 14.2)  -Follow-up: 18-25 | 53/47 | More life-time drinks in binge-drinkers ↑ impulsive choice. |
| Brown et al. (2000) | Cross-sectional | 57 | 15-16 (AUD M_age_: 16.2; Non-AUD M_age_: 15.9) | 58/42 | Lifetime alcohol withdrawal (+) verbal & non-verbal retrieval impairments; recent withdrawal (+) visuospatial functioning impairments. |
| Hanson et al. (2011) | Longitudinal | 213 | 13-27  -Baseline: 13-18 (M_age_: 5.7)  -Follow-up: 16-27 | 54/46 | Heavy alcohol use (+) verbal learning & memory deficits. |
| Parada et al. (2012) | Cross-sectional | 122 | 18-20 (Binge drinking ♂ M_age_: 19.0; Binge drinking ♀ M_age_: 18.7; Non-binge drinking ♂ M_age_: 18.6; Non-binge drinking ♀ M_age_: 18.8) | 52/48 | Binge drinkers ↓ verbal working memory performance & ↑ perseveration. |
| Carbia et al. (2017a) | Longitudinal | 155 | 18-25  -Baseline: 18-19 (M_age_: 18.7)  -Follow-up: 24-25 | 49/51 | Stable binge drinking (+) persistent impairments in immediate & delayed recall. Impairments may improve with long-term abstinence; long-term ex-binge drinker did not display difficulties compared to short-term ex-binge drinkers. |
| Carbia et al. (2017b) | Longitudinal | 155 | 18-25  -Baseline: 18-19 (M_age_: 18.7)  -Follow-up: 24-25 | 49/51 | Stable binge drinking (+) perseverative errors & ↓ working memory span. ↓ working memory improved with or without alcohol abstinence. |
| Khurana et al. (2013) | Longitudinal | 358 | Range N/A  -Baseline M_age_: 11.4  -Follow-up: 4 years later | 48/52 | Greater impulsivity underlying weaker working memory (+) alcohol use over four years. |
| Nguyen-Louie et al. (2016) | Longitudinal | 112 | 12-22  -Baseline: 12-16 (Extreme binge drinker M_age_: 13.8; Binge drinker M_age_: 19.6; Moderate drinker M_age_: 19.5)  -Follow-up: 18-22 (Extreme binge drinker M_age_: 19.7; Binge drinker M_age_: 19.3; Moderate drinker M_age_: 19.7) | 64/36 | Extreme binge drinking deficits (+) in verbal learning & short delayed recall compared to moderate drinkers. |
| Nguyen-Louie et al. (2017) | Longitudinal | 215 | 12-22  -Baseline: 12-15 (M_age_: 13.6)  -Follow-up: 19-22* (M_age_: 20.2) | 59/41 | Early onset of first drinking (+) psychomotor speed & visual attention impairments, while earlier onset of weekly drinking (+) impairments in cognitive inhibition & working memory. |
| Mahedy et al. (2018) | Longitudinal | 4,466 | 15-17  -Baseline M_age_: 15.5  -Follow-up M_age_: 17.8 | N/A | Adolescent frequent/binge drinking (+) working memory impairments at a three-year follow-up. |
| Psychopathology |  |  |  |  |  |
| Briere et al. (2011) | Longitudinal | 6,589 | 12-16  -Baseline: 12-13  (M_age_: 12.8)  -Follow-up: 15-16 | 48/52 | Simultaneous use of alcohol & cannabis in grade 10 (+) substance-related issues the following year compared to concurrent use. |
| Edwards et al. (2014) | Longitudinal | 7,100 | 13-18  -Baseline: 13-15  -Follow-up M_age_: 17.8 | 49/51 | Alcohol use (+) later depression, but not anxiety after adjustment for confounders. |
| Mason et al. (2008) | Longitudinal | 429 | 11-22  -Baseline: 11 (M_age_: 11.0)  -Follow-up: 22 (M_age_: 21.6) | 48/52 | Problem use, but not alcohol intake (+) young adult MDD. |
| Fasteau et al. (2017) | Longitudinal | 1,910 | 16-23  -Baseline: 16  -Follow-up: 23 | 33/67 | Self-reported alcohol use (+) future onset of hypomanic/manic symptoms. |
| Blumenthal et al. (2010) | Cross-sectional | 50 | 12-17 (M_age_: 16.4) | 48/52 | Heightened social anxiety (+) coping-related drinking motives. |
| Schleider et al. (2019) | Longitudinal | 2,100 | 13-17  -Baseline: 13  -Follow-up: 13-17 | 100/0 | Higher depression severity modestly ↑ alcohol use from ages 13 to 17. Alcohol use modestly ↓ odds of depression at ages 14 & 16. Anxiety severity (≠) alcohol use |
| Parrish et al. (2016) | Longitudinal | 674 | 14-16  -Baseline: 14  -Follow-up: 16 | 50/50 | Alcohol use at age 14 (+) overall internalizing symptoms. Overall internalizing symptoms at age 14 (+) alcohol use. |
| Kaplow et al. (2001) | Longitudinal | 936 | 9-17  -Baseline: 9-13  -Follow-up: 13-17 | 55/45 | Early generalized anxiety symptomology (+) risk for the initiation of alcohol use. Early depressive symptomology (+) risk for the initiation of alcohol use in adolescence. |
| Future Substance Use | | | | | |
| Viner and Taylor (2007) | Longitudinal | 4,911 | 16-30  -Baseline: 16  -Follow-up: 30 | 41/59 | Binge drinking (+) risk of alcohol dependence, illicit drug use, & social adversity at age 30. |
| Pampati et al. (2018) | Longitudinal | 2,359 | 13-32  -Baseline: 13-21  -Follow-up: 14-32 | 46/54 | Higher cannabis & alcohol use frequency (+) persistence of cannabis use. |
| Enstad et al. (2019) | Longitudinal | Study 1: 329  Study 2: 789 | Study 1:  14-19  -Baseline: 14-15 (M_age_: 14.6)  -Follow-up: 18-19 (M_age_: 18.9)  Study 2:  13-24  -Baseline: 13.8-16.2 (M_age_: 14.9)  -Follow-up: 21.6-24.6 (M_age_: 22.9) | Study 1:  40/60  Study 2:  N/A | Early onset drinking & early onset of excessive drinking (+) risk of alcohol-related problems in late adolescence/young adulthood. |

Table 2B: Summaries of cross-sectional, retrospective, and longitudinal imaging studies assessing the potential structural and functional vulnerability outcomes of use of alcohol by adolescents.* = estimation; (↑) = increase; (↓) = decrease; (NC) = no change; (+) = positive association; (≠) = no association; male = ♂; female = ♀; M_age_ = mean age.

| Author | Study Type | N | Age | Gender % (♂/♀) | Findings |
| --- | --- | --- | --- | --- | --- |
| Functional | | | | | |
| Ahmadi et al. (2013) | Cross-sectional | 92 | 18-20 (Heavy drinker M_age_: 19.0; Light drinker M_age_: 18.8) | 47/53 | Heavy drinkers ↓ activation during Go/No-Go Task & ↑ impulsivity. |
| Aloi et al. (2019) | Cross-sectional | 150 | 14-18 (M_age_: 16.1) | 61/39 | CUD & AUD ↓ activity in brain regions implicated in error detection & reward processing. |
| Norman et al. (2011) | Longitudinal | 38 | 12-20*  -Baseline: 12-14 (Transitioned to use M_age_: 13.9; Control M_age_: 13.4)  -Follow-up: 16-20* (Transitioned to use M_age_: 18.0; Control M_age_: 17.7) | 50/50 | Lower activation during Go/No-Go task at baseline (+) transition into heavy alcohol consumption. |
| Wetherill et al. (2013) | Longitudinal | 40 | 11-20  -Baseline: 11-16 (Heavy drinker M_age_: 14.7; Non-heavy drinker M_age_: 14.1)  -Follow-up: 14-20 (Heavy drinker M_age_: 18.5; Non-heavy drinker M_age_: 17.6) | 55/45 | Transition into heavy drinking (+) ↓ activation of inhibitory circuitry at baseline & ↑ activation after onset of heavy drinking. |
| Schweinsburg et al. (2010) | Cross-sectional | 24 | 16-18 (Binge drinker M_age_: 18.2; Non-binge drinker M_age_: 17.8) | 75/25 | Binge drinking (+) deficits in recall memory & ↑ activation of frontal & parietal regions, & lack of activation of left hippocampus during novel encoding. |
| Tapert et al. (2004) | Cross-sectional | 34 | 15-17 (AUD M_age_: 16.8; Non-AUD M_age_: 16.5) | 62/38 | AUD (+) differences in brain response to spatial working memory task despite adequate task performance. |
| Squeglia et al. (2012a) | Longitudinal | 40 | 12-21  -Baseline: 12-19 (Heavy drinking transitioner M_age_: 15.1; Continuous non-drinker M_age_: 17.8)  -Follow-up: 12-21 | 70/30 | Lower activation in the frontal & parietal response to working memory (+) transition into heavy drinking. Heavy drinking (+) frontal & parietal response over time. |
| Caldwell et al. (2005) | Cross-sectional | 37 | 14-17 (♂ AUD M_age_: 16.6; ♀ AUD M_age_: 16.9; ♂ non-AUD M_age_: 16.6; ♀ non-AUD M_age_: 16.2) | 62/38 | Differences in brain activation in temporal regions were seen in ♂ & ♀ participants compared to each other & controls during a spatial working memory task despite (NC) in task performance |
| Squeglia et al. (2011) | Cross-sectional | 95 | 16-19 (♂ binge drinker M_age_: 18.1; ♀ binge drinker M_age_: 17.8; ♂ non-binge drinker M_age_: 17.7; ♀ non-binge drinker M_age_: 18.1) | 61/39 | Binge drinking (-) activation in frontal, temporal & cerebellar regions during a spatial memory task, (+) deficits in sustained attention & working memory (♀ only). |
| Aloi et al. (2018) | Cross-sectional | 97 | 14-18 (M_age_: 16.1) | 62/38 | AUD, but not CUD, symptom severity (+) amygdala activity to emotional stimuli. |
| Jurk et al. (2018) | Longitudinal | 131 | 14-18  -Baseline: 14  -Follow-up: 16-18 | 50/50 | Neural & behavioral measures of cognitive control at baseline **(≠)** alcohol use at age 18. |
| Peters et al. (2017) | Longitudinal | Baseline: 292  Follow-up: 254 | 8-27  -Baseline: 8-25 (M_age_: 14.1)  -Follow-up: 10-27 | 48/52 | Greater connectivity between the amygdala & OFC at baseline (+) alcohol use after a two-year follow-up. |
| Jones et al. (2016) | Longitudinal | 26 | 13-19*  -Baseline: 13-16 (M_age_: 14.9)  -Follow-up: 16-19* (M_age_: 17.4) | 62/38 | Binge-drinking (-) activation in the dorsal striatum during decision-making. Lower fronto-parietal activation (+) subsequent emergence into alcohol drinking. |
| Morales et al. (2018) | Longitudinal | 47 | 14-18*  -Baseline: 14-15 (M_age_: 15.1)  -Follow-up: 14-18* | 40/60 | Greater activation in the nucleus accumbens, precuneus, & occipital cortex during risky decision-making (+) earlier initiation of binge drinking. |
| Cservenka et al. (2015) | Longitudinal | 34 | 12-18  -Baseline: 12-16 (M_age_: 14.9)  -Follow-up: 14-18* (M_age_: 16.8) | 58/42 | Binge drinking (-) activity in the left cerebellum. (NC) in ventral striatum activity was observed. |
| Swartz et al. (2020) | Longitudinal | 262 | 16-18  -Baseline: 16 (♂ M_age_: 16.9; ♀ M_age_: 18.9)  -Follow-up: 18 | 51/49 | Differential activation patterns in reward-related neural regions (+) alcohol use from age 16 to 18. |
| Tapert et al. (2003) | Cross-sectional | 30 | 14-17 (AUD M_age_: 17.0; Non-AUD M_age_: 16.4) | 60/40 | AUD (+) brain activation in the left anterior, limbic, & visual systems to alcohol cues. Activation (+) drinks per month & desire to drink. |
| Dager et al. (2014) | Longitudinal | 43 | 18-22  -Baseline: 18-21 (Heavy drinker M_age_: 18.7; Transitioner M_age_: 18.2; Moderate drinker M_age_: 18.5)  -Follow-up: 19-22 | 46/54 | Escalation of alcohol drinking from moderate to heavy (+) BOLD response to alcohol cues in brain regions implicated in cue-reactivity. |
| Brumback et al. (2015) | Longitudinal | 38 | 16-19  -Baseline: 16-19 (Heavy drinker M_age_: 17.9; Non-heavy drinker M_age_: 17.4)  -Follow-up: 16-19 | 46/54 | Heavy drinking (+) BOLD activation to alcohol cues in brain regions implicated in reward processing & decision-making, which disappeared after a month of abstinence |
| Structural |  |  |  |  |  |
| Squeglia et al. (2014) | Longitudinal | 40 | 12-21  -Baseline: 12-17 (Heavy drinker M_age_: 15.1; Non-heavy drinker M_age_: 15.0)  -Follow-up: 15-21 (Heavy drinker M_age_: 18.0; Non-heavy drinker M_age_: 17.2) | 63/37 | Transition to heavy-drinking ↓ frontal brain region volumes at baseline & ↑ volume reductions in subcortical & temporal regions. |
| Cheetham et al. (2014) | Longitudinal | 98 | 11-17  -Baseline: 11.8-13.6 (M_age_: 12.7)  -Follow-up: 15.4-17.6 (M_age_: 16.5) | 48/52 | Smaller left dorsal & rostral paralimbic ACC at age 12 (+) alcohol-related problems at age 16. |
| Jacobus et al. (2009) | Cross-sectional | 42 | Age: 16-19 (M_age_: 17.9) | 86/14 | Binge drinkers ↓ white matter integrity in eight regions. |
| McQueeny et al. (2009) | Cross-sectional | 28 | 16-19 (Binge drinker M_age_: 18.1; Non-binge drinker M_age_: 18.0) | 86/14 | Binge drinking (-) FA in the corpus callosum, cerebellar, temporal, & parietal white matter tracts. |
| Luciana et al. (2013) | Longitudinal | 55 | 14-22  -Baseline: 14-19 (Alcohol initiator M_age_: 16.7; Alcohol non-user M_age_: 17.1)  -Follow-up: 16-22 (Alcohol initiator M_age_: 19.2; Alcohol non-user M_age_: 18.6) | 55/45 | Alcohol initiation (+) greater **↓** in cortical thickness & blunted white matter development. |
| Cardenas et al. (2013) | Cross-sectional | 100 | -AUD M_age_: 15.0  -Non-AUD M_age_: 14.8 | 44/56 | Adolescents with AUD ↑ FA in white matter tracts of the limbic system. |
| De Bellis et al. (2000) | Cross-sectional | 36 | 13.5-21 (Adolescent AUD M_age_: 17.2; No adolescent AUD M_age_: 17.0) | 42/58 | Adolescent onset AUD (+) with smaller left & right hippocampal volume. |
| De Bellis et al. (2005) | Cross-sectional | 42 | 13-21 (AUD M_age_: 17.0; No AUD M_age_: 16.9) | 57/43 | AUD (+) smaller PFC volume & PFC white matter volume; AUD (+) smaller cerebellar volumes in ♂. |
| Nagel et al. (2005) | Cross-sectional | 31 | 15-17 (AUD M_age_: 16.8; Non-AUD M_age_: 16.5) | 61/39 | AUD (+) smaller left hippocampal volume. Hippocampal volume ≠ alcohol consumption rates, suggesting premorbid volumetric differences. |
| Squeglia et al. (2012b) | Cross-sectional | 59 | 16-19 (♂ Binge drinker M_age_: 18.6; ♀ Binge drinker M_age_: 17.8; ♂ Non-binge drinker M_age_: 17.9; ♀ Non-binge drinker M_age_: 18.0) | 51/49 | Binge drinking (+) thicker left frontal cortices, which (+) visuospatial, inhibition, & attention deficits. |
| Lisdahl et al. (2013) | Cross-sectional | 106 | 16-19 (Binge drinker M_age_: 18.0; Non-binge drinker M_age_: 17.7) | 62/38 | Higher peak drinks in binge-drinkers (+) smaller gray & white cerebellar volumes. |
| Pfefferbaum et al. (2018) | Longitudinal | 483 | 12-23  -Baseline: 12-21 (Heavy drinker M_age_: 17.1; Moderate drinker M_age_: 16.7; Maintained Low/No drinker M_age_: 15.1; No/Low drinker M_age_: 15.6)  -Follow-up: 13-23 | 50/50 | Heavy drinking (+) accelerated frontal cortical gray matter trajectory. |

Table 3A: Summaries of cross-sectional, retrospective, and longitudinal studies assessing the potential cognitive, behavioral, psychopathological, and future substance use vulnerability outcomes of use of cannabis use by adolescents* = estimation (↑) = increase; (↓) = decrease; (=) = same as control; (NC) = no change; (+) = positive association; (-) = negative association; (≠) = no association; male = ♂; female = ♀; M_age_ = mean age.

| Author. | Study Type | N | Age | Gender % (♂/♀) | Findings |
| --- | --- | --- | --- | --- | --- |
| Cognition | | | | | |
| Harvey et al. (2007) | Cross-sectional | 70 | 13-18 (M_age_: 16.2) | 40/60 | Regular cannabis use (+) attention, spatial working memory & learning impairments. |
| Lane et al. (2007) | Cross-sectional | 52 | 14-18 (Cannabis user M_age_: 16.8; Cannabis non-user M_age_: 16.2) | 65/35 | Heavy cannabis smoking (+) problem-solving & response adaptation impairments. |
| Medina et al. (2007a) | Cross-sectional | 65 | 16-18 (Cannabis user M_age_: 18.1; Cannabis non-user M_age_: 17.9) | 74/26 | Cannabis use (+) poorer complex attention, story memory, planning, sequencing & motor speed. |
| Infante et al. (2019) | Longitudinal | 175 | 12-29  -Baseline: 12-15  -Follow-up: 12-29 | 57/43 | Lifetime use during adolescence (+) impairments in inhibitory control & visuospatial functioning. (NC) verbal memory. |
| Scott et al. (2017) | Cross-sectional | 4,585 | 14-21 (Frequent cannabis user M_age_: 17.6; Occasional cannabis user M_age_: 17.4; Cannabis non-user M_age_: 16.4) | 44/56 | Frequent & earlier onset, but not occasional cannabis use (+) executive functioning deficits. |
| Hanson et al. (2010) | Longitudinal | 40 | 15-19  -Baseline: 15-19 (Cannabis user M_age_: 18.1; Cannabis non-user M_age_: 17.4)  -Follow-up: 15-19 | 83/17 | Cannabis use poorer (+) verbal learning, verbal working memory & attention accuracy. Verbal learning & verbal working memory deficits improved after three weeks of abstinence. |
| Castellanos-Ryan et al. (2017) | Longitudinal | 295 | 13-20  -Baseline: 13  -Follow-up: 20 | 100/0 | Bidirectional relationship between cannabis use & short-term & working memory. |
| Meier et al. (2012) | Longitudinal | 1,037 | 13-32  -Baseline (Neuropsychological testing): 13  -Follow-up (Neuropsychological testing): 38  -Cannabis use reporting: 18-32 | 52/48 | Cannabis use (-) in IQ. Cessation of use did not fully restore the impairment. |
| Mokrysz et al. (2016) | Longitudinal | 2,235 | 8-15  -IQ score administration: 8, 15  -Cannabis use data: 15 | 47/53 | (NC) in IQ or educational performance after adjusting for cigarette use. |
| Meier et al. (2018) | Longitudinal | 1,989 | 5-18  -Baseline: 5-12  -Follow-up: 18 | 47/53 | Cannabis dependents youth ↓ IQ at ages 12 and 18, but cannabis dependence (**≠)** IQ declines. Executive performance (=) in co-twin pairs at age 18. |
| Jackson et al. (2016) | Longitudinal | Study 1: 789  Study  2: 2,277 | 9-20  -Baseline: 9-10 (M_age_: 9.6)  -Follow-up: 19-20 (M_age_: 19.8)  11-18  -Baseline: 11 (M_age_: 11.8)  -Follow-up: 18 (M_age_: 18.1) | Study 1:  48/52  Study 2:  49/51 | Lower IQ not observed in cannabis-using twins relative to their non-user co-twin, suggesting involvement of familial factors. |
| Duperrouzel et al. (2019) | Longitudinal | 401 | 14-18  -Baseline: 14-17 (M_age_: 15.4)  -Follow-up: 14-19* | 55/45 | Cannabis users ↓ performance in decision-making & episodic memory performance at baseline. Cannabis use (+) worsening of immediate episodic memory. |
| Harvey et al. (2007) | Cross-sectional | 70 | 13-18 (M_age_: 16.2) | 40/60 | Regular cannabis use (+) impairments in attention, spatial working memory, & learning. |
| Medina et al. (2007a) | Cross-sectional | 65 | 16-18 (Cannabis user M_age_: 18.1; Cannabis non-user M_age_: 17.9) | 74/26 | Cannabis use (+) poorer complex attention, story memory planning, sequencing & motor speed. |
| Vo et al. (2014) | Cross-sectional | 42 | 16-20 years (M_age_: 17.9) | 70/30 | CUD (+) working memory impairments. |
| Padula et al. (2007) | Cross-sectional | 34 | 16-18 (Cannabis user M_age_: 18.1; Cannabis non-user M_age_: 17.9) | 77/23 | Cannabis use ≠ differences in working memory but (+) ↑ activation in the right basal ganglia & associated with skill-learning. |
| Pope et al. (2003) | Retrospective | 209 | -Early onset user M_age_: 36  -Late onset user M_age_: 44  -Non-user control M_age_: 40 | 49/51 | Early onset of use (+) poorer cognitive performance. |
| Fontes et al. (2011) | Retrospective | 148 | 18-55 (Early onset user M_age_: 30.4; Late onset user M_age_: 30.0; Non-user M_age_: 27.8) | 68/32 | Early onset of use (+) sustained attention, impulse control & executive function impairments. |
| Noorbakhsh et al. (2020) | Longitudinal | 3826 | 12-17  -Baseline: 12.7  -Follow-up: 17.7* | 53/47 | Age of initiation of use (-) spatial working memory deficits over five years (♀ only). |
| Psychopathology |  |  |  |  |  |
| Arseneault et al. (2002) | Longitudinal | 759 | 15-26  -Baseline: 15,18  -Follow-up: 26 | N/A | Use at age 18 (+) risk for schizophrenia at age 26. |
| Jones et al. (2018) | Longitudinal | 3,328 | -Baseline: 14-19  -Follow-up: 19-24* | N/A | Cannabis use (+) risk of psychotic experiences. |
| Shahzade et al. (2018) | Retrospective | 178 | 18-40 (M_age_: 23.9) | 56/48 | Motives for cannabis use (+) risk of schizophrenia spectrum disorder & schizotypal symptom severity. |
| Hanna et al. (2016) | Retrospective | 161 | 15-65 | 48/52 | Adolescent-onset cannabis use (+) better cognitive function in the schizophrenia/schizoaffective subgroup but not in the bipolar psychosis group. |
| French et al. (2015) | Longitudinal | 1,577 | 12-21  -Baseline: 16  -Follow-up: 18-21 | 57/43 | Early use (-) cortical thickness in ♂ with a high genetic risk for schizophrenia. |
| Albertella et al. (2017) | Longitudinal | 162 | 15-24 (Early onset user M_age_: 19.7; Late onset user M_age_: 20.7) | 41/59 | Early onset use (+) introvertive anhedonia (♀ only). |
| Hiemstra et al. (2018) | Longitudinal | 497 | 13-20  -Baseline: 13  -Follow-up: 13-20 | 57/43 | Genetic vulnerability for schizophrenia (+) cannabis use at age 16-20. |
| Kaasbøll et al. (2018) | Cross-sectional | 33,714 | 13-17 | 50/50 | Cannabis use (+) anxiety & depressive symptoms. Although cannabis use was less prevalent among girls, girls reported more anxiety and depressive symptoms compared to boys. |
| McQueeny et al. (2011) | Cross-sectional | 82 | 16-19 (♂ Cannabis user M_age_: 17.9; ♀ Cannabis user M_age_: 18.2; ♂ Cannabis non-user M_age_: 17.7; ♀ Cannabis non-user M_age_: 17.9) | 77/23 | Cannabis use (+) larger right amygdala volumes & ↑ anxiety/depressive symptoms (♀ only). |
| Hengartner et al. (2020) | Longitudinal | 4,547 | 19-50  -Baseline: 19-20  -Follow-up: 20-50 | 48/52 | Age of onset (-) risk of depression in adulthood. |
| Womack et al. (2016) | Longitudinal | 264 | 17-22  -Baseline: 17  -Follow-up: 20-22 | 100/0 | Cannabis use (+) in later depressive symptoms in those with mild depression. |
| Duperrouzel et al. (2018) | Longitudinal | 401 | 14-18  -Baseline: 14-17 (M_age_: 15.4)  -Follow-up: 15-18 | 56/44 | Levels of early cannabis more (+) persistent self-reported anxiety over time. |
| Assari et al. (2018) | Longitudinal | 681 | 14-19*  -Baseline M_age_: 14.9  -Follow-up: 15-19* | 49/51 | Cannabis use (+) depressive symptoms (♂ only). |
| Future Substance Use | | | | | |
| Degenhardt et al. (2010) | Longitudinal | 1,520 | 13-24  -Baseline: 13-15* (M_age_: 14.9)  -Follow-up: 24-25 (M_age_: 24.1) | 46/54 | Occasional use ↑ risk of developing future alcohol dependence & illicit drug use. |
| Swift et al. (2012) | Longitudinal | 1,756 | 14-29  -Baseline: 14.9-17.4  -Follow-up: 20-29 | 47/53 | Weekly & daily use ↑ rates of illicit drug use & cigarette smoking, respectively. |
| Scholes-Balog et al. (2016) | Longitudinal | 852 | 12-21  -Baseline: 12-19  -Follow-up: 21 | 43/57 | Early onset users ↑ cannabis use frequency & substance-use related harms in young adulthood. |
| Jin et al. (2017) | Longitudinal | 712 | 15-41  -Baseline: 15-19 (Cannabis user M_age_: 17.2; Cannabis non-user M_age_: 16.9)  -Follow-up: 35-41 | 41/59 | Cannabis use at baseline (+) cigarette smoking & alcohol intake the follow-up. |
| Taylor et al. (2017) | Longitudinal | 5,315 | 13-21  -Baseline: 13-18  -Follow-up: 21 | N/A | Users ↑ odd of nicotine dependence, harmful alcohol consumption & illicit drug use. |
| Pampati et al. (2018) | Longitudinal | 2,359 | 13-32  -Baseline: 13-21  -Follow-up: 14-32 | 46/54 | Higher cannabis & alcohol use frequency (+) persistence of cannabis use. |
| Creswell et al. (2015) | Longitudinal | 447 | 12-25  -Baseline: 12-18 (M_age_: 16.2) | 54/46 | Solitary use (+) more frequent cannabis use & more CUD symptoms in young adulthood. |
| Cassidy et al. (2018) | Longitudinal | 1,313 | Baseline M_age_: 18.6 | 45/55 | E-cigarette use (+) initiation of cannabis vaping. Lifetime cannabis & cigarette use (+) e-cigarette use. |
| Solowij et al. (2012) | Cross-sectional | 175 | 16-20 (M_age_: 18.3) | 45/55 | Early onset & greater duration of use (+) impairments in decision-making. |
| Lane et al. (2005) | Cross-sectional | 34 | 14-18 (Cannabis user M_age_: 16.8; Cannabis non-user M_age_: 16.2) | 68/32 | Cannabis users ↓ motivation. |
| Acheson et al. (2015) | Cross-sectional | 28 | M_age_: 17.5 | 79/21 | Users ↑ neural response to both wins & losses in the monetary delay task; Differences in connectivity of 1/3^rd^ of total paths analyzed. |

Table 3B: Summaries of cross-sectional, retrospective, and longitudinal imaging studies assessing the potential structural and functional vulnerability outcomes of use of cannabis by adolescents. * = estimation; (↑) = increase; (↓) = decrease; (=) = same as control; (NC) = no change; (+) = positive association; (-) = negative association; (≠) = no association; male = ♂; female = ♀; M_age_ = mean age.

| Author | Study Type | N | Age | Gender % (♂/♀) | Findings |
| --- | --- | --- | --- | --- | --- |
| Functional | | | | | |
| Behan et al. (2014) | Cross-sectional | 35 | 14-19 (Cannabis user M_age_: 16.5; Cannabis non-user M_age_: 16.1) | 94/6 | Cannabis use (-) inhibitory control, which (+) aberrant activity between bilateral inferior parietal lobule & the left cerebellum. |
| Tapert et al. (2007) | Cross-sectional | 33 | 16-18.9 (Cannabis user M_age_: 18.1; Cannabis non-user M_age_: 17.9) | 73/27 | Cannabis use (≠) differences in inhibitory control task performance but (+) brain processing. |
| Abdullaev et al. (2010) | Cross-sectional; Retrospective | 28 | M_age_: 19.6 | 71/29 | Adolescent onset chronic cannabis use (+) impairments in executive attention & ↑ activation within the right PFC in young adults. |
| Camchong et al. (2017) | Longitudinal | 65 | 10-22*  -Baseline: 10-21  -Follow-up: 11-22* | 57/43 | CUD (-) functional connectivity between caudal ACC with the DLPFC & OFC across time. Functional connectivity between caudal ACC & OFC at baseline (-) cannabis use, which (-) IQ & slower cognitive function |
| Jager et al. (2010) | Cross-sectional | 45 | 13-19 (Cannabis user M_age_: 17.2; Cannabis non-user M_age_: 16.8) | 100/0 | Cannabis users = associative learning & working memory tasks; ↑ activity levels in the prefrontal regions when the working memory task was novel. |
| Jacobsen et al. (2004) | Cross-sectional | 21 | -Cannabis user M_age_: 17.4  -Tobacco smoker M_age_: 17.1  -Non-smoker M_age_: 16.8 | 38/62 | Cannabis use (+) impairments in sustained attention & working memory; failure to reduce activity in right hippocampus during working memory task. |
| Tervo-Clemmens et al. (2018) | Longitudinal | 75 | 14-28  -Baseline: 14-22  -Follow-up: 28 | 46/64 | Age of onset of use (-) reaction times & ↓ activation in the posterior parietal cortex. |
| Spechler et al. (2015) | Cross-sectional | 140 | M_age_: 14.7 | 65/35 | Cannabis use (+) greater sensitivity to angry faces as indicated by ↑ reactivity in the bilateral amygdala. |
| Aloi et al. (2018) | Cross-sectional | 97 | 14-18 (M_age_: 16.1) | 62/38 | AUD, nut not CUD, symptom severity (+) amygdala activity to emotional stimuli. |
| Subramaniam et al. (2018) | Cross-sectional | 74 | 14-20 (Cannabis user M_age_: 18.0; Cannabis non-user M_age_: 17.1) | 80/20 | Users exhibited alterations in OFC connectivity, (+) depression & anxiety symptoms. |
| Lichenstein et al. (2017) | Longitudinal | 158 | Adolescent cannabis use data collection: 14-19  -fMRI and substance use data collection: 20  -Psychological outcomes data collection: 22 | 100/0 | Escalating cannabis use (+) negative functional connectivity between the nucleus accumbens & medical PFC, (+) higher depressive symptoms & anhedonia. |
| De Bellis et al. (2013) | Cross-sectional | 56 | 13-17 | 100/0 | CUD (+) hyperactivation in brain regions implicated in risky & uncertain decision-making. |
| Jager et al. (2013) | Cross-sectional | 45 | 13-19 (Cannabis user M_age_: 17.2; Cannabis non-user M_age_: 16.8) | 100/0 | Cannabis users = in monetary incentive delay task assessing reward-related brain function; striatal hyperactivity during the anticipatory stages of reward. |
| Nestor et al. (2019) | Cross-sectional | 36 | -Cannabis user M_age_: 16.50  -Cannabis non-user M_age_: 16.11 | 94/6 | Cannabis dependent adolescents ↑ performance & ↑ global connectivity during the anticipatory stages of monetary incentive delay task. |
| Structural |  |  |  |  |  |
| Medina et al. (2010) | Cross-sectional | 32 | 16-18 (M_age_: 18.0) | 68/31 | Users ↑ inferior posterior vermis & larger posterior cerebellar vermis volumes, which in turn (+) poorer executive functions. |
| Medina et al. (2009) | Cross-sectional | 32 | 16-18 (M_age_: 18.0) | 69/31 | Users ↑ (♂) & ↓ (♀) PFC volumes. PFC volume (-) better executive functioning among users. |
| Churchwell et al. (2010) | Cross-sectional | 36 | 16-19 (Cannabis abuser M_age_: 17.7; Cannabis non-abuser M_age_: 17.2) | 78/22 | Cannabis use (-) right medial orbital PFC volume. Medial orbital PFC volume (+) age of first use. |
| Ashtari et al. (2011) | Cross-sectional | 28 | 18-20 (M_age_: 18.9) | 100/0 | Heavy use (+) smaller left hippocampus volumes. (NC) in bilateral amygdala volume. |
| Weiland et al. (2015) | Cross-sectional | Adults: 68  Youth: 100 | Adolescent: 14-18 (Daily cannabis user M_age_: 16.7; Cannabis non-user M_age_: 16.8)  -Adult: 18-53 (Daily cannabis user M_age_: 27.4; Cannabis non-user M_age_: 27.6) | Adults: 77/23  Youth: 55/45 | (NC) in accumbens, amygdala, hippocampus, or cerebellum volumes. |
| Scott et al. (2019) | Cross-sectional | 781 | 14-22 (Frequent cannabis user M_age_: 18.5; Occasional cannabis user M_age_: 18.1; Cannabis non-user M_age_: 17.0) | 43/57 | (NC) in global or regional brain volumes, cortical thickness, and gray matter density. |
| Becker et al. (2015) | Longitudinal | 46 | 18-22  -Baseline: 18-20 (M_age_: 19.3*)  -Follow-up: 20-22 | 70/30 | Cannabis use (-) longitudinal growth of FA in the left superior longitudinal fasciculus, left corticospinal tract, right anterior thalamic radiation & poor verbal learning performance. |
| Ashtari et al. (2009) | Cross-sectional | 28 | 17-21 (M_age_: 18.9) | 100/0 | Heavy cannabis history ↓ FA, as well as ↑ radial diffusivity & increased trace in frontotemporal white matter circuits. |
| Epstein and Kumra (2015) | Longitudinal | 79 | -Baseline: 10-23 (CUD M_age_: 16.4; Non-CUD M_age_: 17.0)  -Follow-up: 12-25 | 54/46 | CUD (-) normative loss of cortical thickness in the heteromodal association cortex. |
| Orr et al. (2019) | Longitudinal | 92 | 14-16  -Baseline: 14  -Follow-up: 16 | 75/25 | Users ↑ GMV in the bilateral medial temporal lobes, bilateral posterior cingulate, lingual gyri, & cerebellum. |
| Wilson et al. (2000) | Retrospective | 57 | 19-48 (♂ Early onset cannabis user M_age_: 31.5; ♀ Early onset cannabis user M_age_: 27.9; ♂ Late onset cannabis user M_age_: 33.2; ♀ Late onset cannabis user M_age_: 33.1) | 56/44 | Age of onset of use (+) whole brain & percent cortical gray matter, (-) percent white matter volume. |
| Meier et al. (2019) | Longitudinal | 181 | 13-36  -Baseline: 13-19  -Follow-up: 30-36 | 100/0 | Cannabis use (≠) structural differences in subcortical or cortical brain regions. |
| Kumra et al. (2012) | Cross-sectional | 115 | 10-21 (Early onset schizophrenia with CUD M_age_: 17.5; CUD M_age_: 16.6; Early onset schizophrenia M_age_: 16.5; Control M_age_: 16.2) | 53/47 | Age of onset schizophrenia & CUD (-) GMV in the left superior parietal cortex. |
| Medina et al. (2007b) | Cross-sectional | 32 | 16-18 (M_age_: 18) | 72/28 | Cannabis use (+) depressive symptoms, (-) white matter volume; white matter volume (-) depressive symptoms. |
| Cheetham et al. (2012) | Longitudinal | 121 | 12-16  -Baseline: 12 (M_age_: 12.7)  -Follow-up: 16 (M_age_: 16.5) | 51/49 | Smaller OFC volumes at age 12 ↑ initiation of cannabis use by age 16. |

Table 4: Summaries of cross-sectional, retrospective, and longitudinal studies assessing the potential cognitive, behavioral, psychopathological, and future substance use vulnerability outcomes of use of opioids by adolescents (↓) = decrease; (+) = positive association; male = ♂; female = ♀; M_age_ = mean age.

| Author | Study Type | N | Age | Gender % (♂/♀) | Findings |
| --- | --- | --- | --- | --- | --- |
| **Cognition** | | | | | |
| Vo et al. (2014) | Cross-sectional | 42 | 16-20 (M_age_: 18.7) | 70/30 | Opioid use disorder ↓ working memory. |
| **Psychopathology** | | | | | |
| Edlund et al. (2015) | Cross-sectional | 112,600 | 12-17 (M_age_: 14.5) | 51/49 | Opioid use (+) major depression. |
| Subramaniam and Stitzer (2009) | Cross-sectional | 94 | 14-18 (M_age_: 17) | 100/0 | Heroin & prescription opioid use (+) comorbid psychiatric disorders, multiple SUD, ADHD, manic episodes, criminal behavior, major depressive episode. |
| Subramaniam et al. (2009) | Cross-sectional | 168 | 14-18 (M_age_: 16.75) | 61/39 | Opioid dependence (+) substance use, depressive symptoms, & decreased academic success. |
| **Future Substance Use** | | | | | |
| Cerda et al. (2015) | Cross-sectional | 223,534 | 12-21 (M_age_: 16.5) | 51/49 | Misuse of prescription opioids (+) future heroin use. |
| Miech et al. (2015) | Longitudinal | 6,220 | 17-23  Baseline: 17-19  Follow-up: 23 | N/A | Legitimate use of prescription opioids (+) risk of future misuse. |

Table 5A: Summaries of cross-sectional, retrospective, and longitudinal studies assessing the potential cognitive, behavioral, psychopathological, and future substance use vulnerability outcomes of co-use of substances by adolescents. ^AC^ = alcohol and cannabis co-use; ^AN^ = alcohol and nicotine products co-use; ^all^ = co-use of three or more substances; * = estimation; (↑) = increase; (↓) = decrease; (+) = positive association; male = ♂; female = ♀; M_age_ = mean age.

| Author | Study Type | N | Age | Gender % (♂/♀) | Findings |
| --- | --- | --- | --- | --- | --- |
| Cognition | | | | | |
| Jacobus et al. (2015)^AC^ | Longitudinal | 108 | 16-19 (M_age_: 17.8 (baseline), 20.8 (follow-up)) | 65/35 | Co-use ↓ complex attention, memory, processing speed, & visuospatial functioning. |
| Mahmood et al. (2010)^AC^ | Cross-sectional | 130 | 15-19 (M_age_: 17.8) | 75/25 | Heavy alcohol users ↓ verbal learning & memory during hangover. Deficits not seen in co-users. |
| Morin et al. (2019)^AC^ | Longitudinal | 3,826 | 12-17 (M_age_: 12.7 (baseline), 16.7 (follow-up)) | 53/47 | Cannabis but not alcohol ↓ working memory & inhibitory control (short-term); ↓ perceptual reasoning & delayed memory recall (long-term). |
| Terry-McElrath et al. (2014)^AC^ | Cross-sectional | 72,053 | 17-19 | 47/53 | Simultaneous use ↑ risk for unsafe driving vs. concurrent or alcohol only. |
| Winward et al. (2014)^AC^ | Cross-sectional | 128 | 16-18 (M_age_: 17.8) | 65/35 | Following abstinence, heavy co-users ↓ working memory vs. individual users. |
| **Psychopathology** |  |  |  |  |  |
| Boys et al. (2003)^all^ | Cross-sectional | 2,624 | 13-15 (M_age_: 14) | 50/50 | Tobacco, alcohol & cannabis (+) additive risk for depressive disorders. Except for cannabis & alcohol, all co-use (+) risk for any psychiatric disorder. |
| Matuszka et al. (2016)^AN^ | Cross-sectional | 944 | 14-16 (M_age_: 15.04) | N/A | Co-use additively ↑ physical aggression. |
| **Future Substance Use** | | | | | |
| Briere et al. (2011)^AC^ | Longitudinal | 6,589 | 12-17 (M_age_: 12.8 (baseline), 15.5 (follow-up)) | 48/52 | Simultaneous use (+) future substance-related issues vs. concurrent use. |
| Green et al. (2016) | Longitudinal | 608 | 6-25  Baseline: 6  Follow-up: 19-25 | 54/46 | Co-use (+) future negative outcomes in young adulthood in areas of substance use, graduation, criminal record, income & employment. |
| Grucza and Bierut (2006)^AN^ | Cross-sectional | 74,836 | 12-20 (M_age_: 15*) | 51/49 | Past-year smokers drank more alcohol than non-smokers & ↑ risk for AUD compared to non-smokers who drank equivalent amounts. |
| Patrick et al. (2018)^AC^ | Cross-sectional | 84,805 | 17-19 | 52/48 | Simultaneous use (+) level of illicit substance use other than cannabis, truancy, & nights out. |
| Rubinstein et al. (2014)^CN^ | Cross-sectional | 165 | 13-17 (M_age_: 16.1) | 48/52 | Cannabis use (+) reported nicotine addiction among adolescent smokers. |
| Schmid et al. (2007)^AN^ | Cross-sectional | 384 | 14-16 (M_age_: 14.9 (baseline)) | 48/52 | Alcohol & tobacco users ↑ alcohol use, initiated alcohol use at a younger age, ↑ cannabis use, had ↑ nicotine dependence scores & novelty seeking vs. alcohol users. |

Table 5B: Summaries of cross-sectional, retrospective, and longitudinal imaging studies assessing the potential structural and functional vulnerability outcomes of co-use of substances by adolescents. ^AC^ = alcohol & cannabis co-use; ^AN^ = alcohol & nicotine products co-use; ^all^ = co-use of three or more substances. * = estimation; (↑) = Increase; (↓) = decrease; (=) = same as control; (NC) = no change; (+) = positive association; (-) = negative association; male= ♂; ♀= female; M_age_ = mean age.

| Author | Study Type | N | Age | Gender % (♂/♀) | Findings |
| --- | --- | --- | --- | --- | --- |
| Structural | | | | | |
| Bava et al. (2009) ^AC^ | Cross-sectional | 72 | 16-19 (M_age_: 17.9) | 28/72 | Co-users ↓ FA in 10 regions, ↑ FA in three regions, ↓ mean diffusivity in one region, & ↑ mean diffusivity in one region. |
| Bava et al. (2010) ^AC^ | Cross-sectional | 72 | 16-19 (M_age_: 17.9) | 28/72 | Co-users ↓ FA in temporal brain areas, which was (-) attention, working memory & speeded processing. ↑ FA in occipital regions (+) working memory & complex sequencing. |
| Bava et al. (2013)^AC^ | Longitudinal | 92 | 16-20 (M_age_: 18.1 (baseline), 19.6 (follow-up)) | 68/32 | Co-users ↓ white matter integrity in seven tracts. More alcohol use ↓ integrity in right & left superior longitudinal fasciculi. |
| Infante et al. (2019)^AC^ | Longitudinal | 69 | 12-21 (M_age_: 13.7 (baseline), 19.2 (follow-up)) | 56.5/43.5 | Alcohol only ↓ OFC grey matter surface area. |
| Jacobus et al. (2009)^AC^ | Cross-sectional | 42 | 16-19 (M_age_: 17.9) | 86/14 | Binge drinking ↓ FA in eight regions. Co-users ↑ FA vs. binge drinkers in four regions. |
| Jacobus et al. (2013a)^AC^ | Longitudinal | 54 | 16-22 (M_age_: 17.9 (baseline), 20.9 (follow-up)) | 59/41 | Binge drinkers & co-users had similarly ↓ FA. |
| Jacobus et al. (2013b)^AC^ | Longitudinal | 16 | 16-22  (M_age_: 17.9 (Baseline), 20.9 (follow-up)) | N/A | Teens who would go on to initiate co-use showed FA > or = to alcohol only initiation; co-use initiation ↓ FA. |
| Jacobus et al. (2014)^AC^ | Cross-sectional;  Retrospective | 54 | 15-18 (M_age_: 17.5) | 72/28 | Co-use ↑ cortical thickness in left entorhinal cortex. Cannabis use ↓ cortical thickness in frontal & temporal lobes. Alcohol use ↑ cortical thickness in all regions. |
| Jacobus et al. (2015)^AC^ | Longitudinal | 68 | 16-22  Baseline: 16-19 (M_age_: 17.9 (Baseline), 21 (follow-up)) | 70/30 | Co-use ↑ cortical thickness in 23 regions. Cannabis ↑ thickness; alcohol ↓ thickness. |
| Jacobus et al. (2016)^AC^ | Longitudinal | 69 | 12-21 (M_age_: 13.7 (baseline), 19.2 (follow-up)) | 56.5/43.5 | Alcohol initiators & controls ↑ cortical thickness at baseline. Larger decreases in thickness observed in alcohol initiators & controls vs. co-use initiators. |
| Medina et al. (2007c)^AC^ | Cross-sectional | 63 | 15-18 (M_age_: 17.3) | 65/35 | Alcohol users ↑ right > left asymmetry & ↓ left hippocampal volume. Cannabis ↑ left > right asymmetry & ↑ left hippocampal volume. Co-users (NC) in symmetry or volume. |
| **Functional** |  |  |  |  |  |
| Claus et al. (2018)^AC^ | Cross-sectional | 189 | 14-18 (M_age_: 16.2) | 75/25 | Co-use ↓ BOLD response in thalamus, insula & striatum during balloon analog risk-taking task. |
| Karoly et al. (2015)^all^ | Cross-sectional | 239 | 14-18 (M_age_: 15.9) | 65/35 | Tobacco use ↓ BOLD response in nucleus accumbens during monetary incentive delay task. (NC) in tobacco+cannabis/ tobacco+cannabis+alcohol. |
| Schweinsburg et al. (2005)^AC^ | Cross-sectional | 49 | 15-17 (M_age_: 16.7) | 67/33 | Alcohol+cannabis use disorder ↓ BOLD activation in inferior frontal & temporal regions & ↑ activation in medial frontal regions vs. AUD |
| Schweinsburg et al. (2011)^AC^ | Cross-sectional | 74 | 16-18 (M_age_: 18) | 80/20 | Binge drinking or cannabis use ↑ BOLD response in bilateral frontal regions during verbal-paired associates encoding task. Co-users (=) non-users. |

**References for Tables:**

Abdullaev, Y., Posner, M.I., Nunnally, R., and Dishion, T.J. (2010). Functional MRI evidence for inefficient attentional control in adolescent chronic cannabis abuse. *Behav Brain Res* 215**,** 45-57.

Acheson, A., Ray, K.L., Hines, C.S., Li, K., Dawes, M.A., Mathias, C.W., Dougherty, D.M., and Laird, A.R. (2015). Functional activation and effective connectivity differences in adolescent marijuana users performing a simulated gambling task. *J Addict* 2015**,** 783106.

Ahmadi, A., Pearlson, G.D., Meda, S.A., Dager, A., Potenza, M.N., Rosen, R., Austad, C.S., Raskin, S.A., Fallahi, C.R., Tennen, H., Wood, R.M., and Stevens, M.C. (2013). Influence of alcohol use on neural response to Go/No-Go task in college drinkers. *Neuropsychopharmacology* 38**,** 2197-2208.

Albertella, L., Le Pelley, M.E., and Copeland, J. (2017). Cannabis use in early adolescence is associated with higher negative schizotypy in females. *Eur Psychiatry* 45**,** 235-241.

Aloi, J., Blair, K.S., Crum, K.I., Meffert, H., White, S.F., Tyler, P.M., Thornton, L.C., Mobley, A.M., Killanin, A.D., Adams, K.O., Filbey, F., Pope, K., and Blair, R.J.R. (2018). Adolescents show differential dysfunctions related to Alcohol and Cannabis Use Disorder severity in emotion and executive attention neuro-circuitries. *Neuroimage Clin* 19**,** 782-792.

Aloi, J., Meffert, H., White, S.F., Blair, K.S., Hwang, S., Tyler, P.M., Thornton, L.C., Crum, K.I., Adams, K.O., Killanin, A.D., Filbey, F., Pope, K., and Blair, R.J.R. (2019). Differential dysfunctions related to alcohol and cannabis use disorder symptoms in reward and error-processing neuro-circuitries in adolescents. *Dev Cogn Neurosci* 36**,** 100618.

Arseneault, L., Cannon, M., Poulton, R., Murray, R., Caspi, A., and Moffitt, T.E. (2002). Cannabis use in adolescence and risk for adult psychosis: longitudinal prospective study. *BMJ* 325**,** 1212-1213.

Ashtari, M., Avants, B., Cyckowski, L., Cervellione, K.L., Roofeh, D., Cook, P., Gee, J., Sevy, S., and Kumra, S. (2011). Medial temporal structures and memory functions in adolescents with heavy cannabis use. *J Psychiatr Res* 45**,** 1055-1066.

Ashtari, M., Cervellione, K., Cottone, J., Ardekani, B.A., Sevy, S., and Kumra, S. (2009). Diffusion abnormalities in adolescents and young adults with a history of heavy cannabis use. *J Psychiatr Res* 43**,** 189-204.

Assari, S., Mistry, R., Caldwell, C.H., and Zimmerman, M.A. (2018). Marijuana Use and Depressive Symptoms; Gender Differences in African American Adolescents. *Front Psychol* 9**,** 2135.

Bava, S., Frank, L.R., Mcqueeny, T., Schweinsburg, B.C., Schweinsburg, A.D., and Tapert, S.F. (2009). Altered white matter microstructure in adolescent substance users. *Psychiatry Res* 173**,** 228-237.

Bava, S., Jacobus, J., Mahmood, O., Yang, T.T., and Tapert, S.F. (2010). Neurocognitive correlates of white matter quality in adolescent substance users. *Brain Cogn* 72**,** 347-354.

Bava, S., Jacobus, J., Thayer, R.E., and Tapert, S.F. (2013). Longitudinal changes in white matter integrity among adolescent substance users. *Alcohol Clin Exp Res* 37 Suppl 1**,** E181-189.

Becker, M.P., Collins, P.F., Lim, K.O., Muetzel, R.L., and Luciana, M. (2015). Longitudinal changes in white matter microstructure after heavy cannabis use. *Dev Cogn Neurosci* 16**,** 23-35.

Behan, B., Connolly, C.G., Datwani, S., Doucet, M., Ivanovic, J., Morioka, R., Stone, A., Watts, R., Smyth, B., and Garavan, H. (2014). Response inhibition and elevated parietal-cerebellar correlations in chronic adolescent cannabis users. *Neuropharmacology* 84**,** 131-137.

Benowitz, N.L., Nardone, N., Jain, S., Dempsey, D.A., Addo, N., St Helen, G., and Jacob, P., 3rd (2018). Comparison of Urine 4-(Methylnitrosamino)-1-(3)Pyridyl-1-Butanol and Cotinine for Assessment of Active and Passive Smoke Exposure in Urban Adolescents. *Cancer Epidemiol Biomarkers Prev* 27**,** 254-261.

Blumenthal, H., Leen-Feldner, E.W., Frala, J.L., Badour, C.L., and Ham, L.S. (2010). Social anxiety and motives for alcohol use among adolescents. *Psychol Addict Behav* 24**,** 529-534.

Boykan, R., Messina, C.R., Chateau, G., Eliscu, A., Tolentino, J., and Goniewicz, M.L. (2019). Self-Reported Use of Tobacco, E-cigarettes, and Marijuana Versus Urinary Biomarkers. *Pediatrics* 143.

Boys, A., Farrell, M., Taylor, C., Marsden, J., Goodman, R., Brugha, T., Bebbington, P., Jenkins, R., and Meltzer, H. (2003). Psychiatric morbidity and substance use in young people aged 13-15 years: results from the Child and Adolescent Survey of Mental Health. *Br J Psychiatry* 182**,** 509-517.

Briere, F.N., Fallu, J.S., Descheneaux, A., and Janosz, M. (2011). Predictors and consequences of simultaneous alcohol and cannabis use in adolescents. *Addict Behav* 36**,** 785-788.

Brown, S.A., Tapert, S.F., Granholm, E., and Delis, D.C. (2000). Neurocognitive functioning of adolescents: effects of protracted alcohol use. *Alcohol Clin Exp Res* 24**,** 164-171.

Brumback, T., Squeglia, L.M., Jacobus, J., Pulido, C., Tapert, S.F., and Brown, S.A. (2015). Adolescent heavy drinkers' amplified brain responses to alcohol cues decrease over one month of abstinence. *Addict Behav* 46**,** 45-52.

Caldwell, L.C., Schweinsburg, A.D., Nagel, B.J., Barlett, V.C., Brown, S.A., and Tapert, S.F. (2005). Gender and adolescent alcohol use disorders on BOLD (blood oxygen level dependent) response to spatial working memory. *Alcohol Alcohol* 40**,** 194-200.

Camchong, J., Lim, K.O., and Kumra, S. (2017). Adverse Effects of Cannabis on Adolescent Brain Development: A Longitudinal Study. *Cereb Cortex* 27**,** 1922-1930.

Carbia, C., Cadaveira, F., Caamano-Isorna, F., Rodriguez-Holguin, S., and Corral, M. (2017a). Binge drinking during adolescence and young adulthood is associated with deficits in verbal episodic memory. *PLoS One* 12**,** e0171393.

Carbia, C., Cadaveira, F., Lopez-Caneda, E., Caamano-Isorna, F., Rodriguez Holguin, S., and Corral, M. (2017b). Working memory over a six-year period in young binge drinkers. *Alcohol* 61**,** 17-23.

Cardenas, V.A., Greenstein, D., Fouche, J.P., Ferrett, H., Cuzen, N., Stein, D.J., and Fein, G. (2013). Not lesser but Greater fractional anisotropy in adolescents with alcohol use disorders. *Neuroimage Clin* 2**,** 804-809.

Cassidy, R.N., Meisel, M.K., Diguiseppi, G., Balestrieri, S., and Barnett, N.P. (2018). Initiation of vaporizing cannabis: Individual and social network predictors in a longitudinal study of young adults. *Drug Alcohol Depend* 188**,** 334-340.

Castellanos-Ryan, N., Pingault, J.B., Parent, S., Vitaro, F., Tremblay, R.E., and Seguin, J.R. (2017). Adolescent cannabis use, change in neurocognitive function, and high-school graduation: A longitudinal study from early adolescence to young adulthood. *Dev Psychopathol* 29**,** 1253-1266.

Cerda, M., Santaella, J., Marshall, B.D., Kim, J.H., and Martins, S.S. (2015). Nonmedical Prescription Opioid Use in Childhood and Early Adolescence Predicts Transitions to Heroin Use in Young Adulthood: A National Study. *J Pediatr* 167**,** 605-612 e601-602.

Cheetham, A., Allen, N.B., Whittle, S., Simmons, J., Yucel, M., and Lubman, D.I. (2014). Volumetric differences in the anterior cingulate cortex prospectively predict alcohol-related problems in adolescence. *Psychopharmacology (Berl)* 231**,** 1731-1742.

Cheetham, A., Allen, N.B., Whittle, S., Simmons, J.G., Yucel, M., and Lubman, D.I. (2012). Orbitofrontal volumes in early adolescence predict initiation of cannabis use: a 4-year longitudinal and prospective study. *Biol Psychiatry* 71**,** 684-692.

Churchwell, J.C., Lopez-Larson, M., and Yurgelun-Todd, D.A. (2010). Altered frontal cortical volume and decision making in adolescent cannabis users. *Front Psychol* 1**,** 225.

Claus, E.D., Feldstein Ewing, S.W., Magnan, R.E., Montanaro, E., Hutchison, K.E., and Bryan, A.D. (2018). Neural mechanisms of risky decision making in adolescents reporting frequent alcohol and/or marijuana use. *Brain Imaging Behav* 12**,** 564-576.

Creswell, K.G., Chung, T., Clark, D.B., and Martin, C.S. (2015). Solitary cannabis use in adolescence as a correlate and predictor of cannabis problems. *Drug Alcohol Depend* 156**,** 120-125.

Cservenka, A., Jones, S.A., and Nagel, B.J. (2015). Reduced cerebellar brain activity during reward processing in adolescent binge drinkers. *Dev Cogn Neurosci* 16**,** 110-120.

Dager, A.D., Anderson, B.M., Rosen, R., Khadka, S., Sawyer, B., Jiantonio-Kelly, R.E., Austad, C.S., Raskin, S.A., Tennen, H., Wood, R.M., Fallahi, C.R., and Pearlson, G.D. (2014). Functional magnetic resonance imaging (fMRI) response to alcohol pictures predicts subsequent transition to heavy drinking in college students. *Addiction* 109**,** 585-595.

De Bellis, M.D., Clark, D.B., Beers, S.R., Soloff, P.H., Boring, A.M., Hall, J., Kersh, A., and Keshavan, M.S. (2000). Hippocampal volume in adolescent-onset alcohol use disorders. *Am J Psychiatry* 157**,** 737-744.

De Bellis, M.D., Narasimhan, A., Thatcher, D.L., Keshavan, M.S., Soloff, P., and Clark, D.B. (2005). Prefrontal cortex, thalamus, and cerebellar volumes in adolescents and young adults with adolescent-onset alcohol use disorders and comorbid mental disorders. *Alcohol Clin Exp Res* 29**,** 1590-1600.

De Bellis, M.D., Wang, L., Bergman, S.R., Yaxley, R.H., Hooper, S.R., and Huettel, S.A. (2013). Neural mechanisms of risky decision-making and reward response in adolescent onset cannabis use disorder. *Drug Alcohol Depend* 133**,** 134-145.

Degenhardt, L., Coffey, C., Carlin, J.B., Swift, W., Moore, E., and Patton, G.C. (2010). Outcomes of occasional cannabis use in adolescence: 10-year follow-up study in Victoria, Australia. *Br J Psychiatry* 196**,** 290-295.

Duperrouzel, J., Hawes, S.W., Lopez-Quintero, C., Pacheco-Colon, I., Comer, J., and Gonzalez, R. (2018). The association between adolescent cannabis use and anxiety: A parallel process analysis. *Addict Behav* 78**,** 107-113.

Duperrouzel, J.C., Hawes, S.W., Lopez-Quintero, C., Pacheco-Colon, I., Coxe, S., Hayes, T., and Gonzalez, R. (2019). Adolescent cannabis use and its associations with decision-making and episodic memory: Preliminary results from a longitudinal study. *Neuropsychology* 33**,** 701-710.

Edlund, M.J., Forman-Hoffman, V.L., Winder, C.R., Heller, D.C., Kroutil, L.A., Lipari, R.N., and Colpe, L.J. (2015). Opioid abuse and depression in adolescents: Results from the National Survey on Drug Use and Health. *Drug Alcohol Depend* 152**,** 131-138.

Edwards, A.C., Heron, J., Dick, D.M., Hickman, M., Lewis, G., Macleod, J., and Kendler, K.S. (2014). Adolescent alcohol use is positively associated with later depression in a population-based U.K. cohort. *J Stud Alcohol Drugs* 75**,** 758-765.

Enstad, F., Evans-Whipp, T., Kjeldsen, A., Toumbourou, J.W., and Von Soest, T. (2019). Predicting hazardous drinking in late adolescence/young adulthood from early and excessive adolescent drinking - a longitudinal cross-national study of Norwegian and Australian adolescents. *BMC Public Health* 19**,** 790.

Epstein, K.A., and Kumra, S. (2015). Altered cortical maturation in adolescent cannabis users with and without schizophrenia. *Schizophr Res* 162**,** 143-152.

Fasteau, M., Mackay, D., Smith, D.J., and Meyer, T.D. (2017). Is adolescent alcohol use associated with self-reported hypomanic symptoms in adulthood? - Findings from a prospective birth cohort. *Psychiatry Res* 255**,** 232-237.

Field, M., Christiansen, P., Cole, J., and Goudie, A. (2007). Delay discounting and the alcohol Stroop in heavy drinking adolescents. *Addiction* 102**,** 579-586.

Fontes, M.A., Bolla, K.I., Cunha, P.J., Almeida, P.P., Jungerman, F., Laranjeira, R.R., Bressan, R.A., and Lacerda, A.L. (2011). Cannabis use before age 15 and subsequent executive functioning. *Br J Psychiatry* 198**,** 442-447.

French, L., Gray, C., Leonard, G., Perron, M., Pike, G.B., Richer, L., Seguin, J.R., Veillette, S., Evans, C.J., Artiges, E., Banaschewski, T., Bokde, A.W., Bromberg, U., Bruehl, R., Buchel, C., Cattrell, A., Conrod, P.J., Flor, H., Frouin, V., Gallinat, J., Garavan, H., Gowland, P., Heinz, A., Lemaitre, H., Martinot, J.L., Nees, F., Orfanos, D.P., Pangelinan, M.M., Poustka, L., Rietschel, M., Smolka, M.N., Walter, H., Whelan, R., Timpson, N.J., Schumann, G., Smith, G.D., Pausova, Z., and Paus, T. (2015). Early Cannabis Use, Polygenic Risk Score for Schizophrenia and Brain Maturation in Adolescence. *JAMA Psychiatry* 72**,** 1002-1011.

Green, K.M., Musci, R.J., Johnson, R.M., Matson, P.A., Reboussin, B.A., and Ialongo, N.S. (2016). Outcomes associated with adolescent marijuana and alcohol use among urban young adults: A prospective study. *Addict Behav* 53**,** 155-160.

Grucza, R.A., and Bierut, L.J. (2006). Cigarette smoking and the risk for alcohol use disorders among adolescent drinkers. *Alcohol Clin Exp Res* 30**,** 2046-2054.

Hanna, R.C., Shalvoy, A., Cullum, C.M., Ivleva, E.I., Keshavan, M., Pearlson, G., Hill, S.K., Sweeney, J.A., Tamminga, C.A., and Ghose, S. (2016). Cognitive Function in Individuals With Psychosis: Moderation by Adolescent Cannabis Use. *Schizophr Bull* 42**,** 1496-1503.

Hanson, K.L., Cummins, K., Tapert, S.F., and Brown, S.A. (2011). Changes in neuropsychological functioning over 10 years following adolescent substance abuse treatment. *Psychol Addict Behav* 25**,** 127-142.

Hanson, K.L., Winward, J.L., Schweinsburg, A.D., Medina, K.L., Brown, S.A., and Tapert, S.F. (2010). Longitudinal study of cognition among adolescent marijuana users over three weeks of abstinence. *Addict Behav* 35**,** 970-976.

Harvey, M.A., Sellman, J.D., Porter, R.J., and Frampton, C.M. (2007). The relationship between non-acute adolescent cannabis use and cognition. *Drug Alcohol Rev* 26**,** 309-319.

Hengartner, M.P., Angst, J., Ajdacic-Gross, V., and Rössler, W. (2020). Cannabis use during adolescence and the occurrence of depression, suicidality and anxiety disorder across adulthood: Findings from a longitudinal cohort study over 30 years. *Journal of Affective Disorders*.

Hiemstra, M., Nelemans, S.A., Branje, S., Van Eijk, K.R., Hottenga, J.J., Vinkers, C.H., Van Lier, P., Meeus, W., and Boks, M.P. (2018). Genetic vulnerability to schizophrenia is associated with cannabis use patterns during adolescence. *Drug Alcohol Depend* 190**,** 143-150.

Infante, M.A., Nguyen-Louie, T.T., Worley, M., Courtney, K.E., Coronado, C., and Jacobus, J. (2019). Neuropsychological Trajectories Associated with Adolescent Alcohol and Cannabis Use: A Prospective 14-Year Study. *J Int Neuropsychol Soc***,** 1-12.

Jackson, N.J., Isen, J.D., Khoddam, R., Irons, D., Tuvblad, C., Iacono, W.G., Mcgue, M., Raine, A., and Baker, L.A. (2016). Impact of adolescent marijuana use on intelligence: Results from two longitudinal twin studies. *Proc Natl Acad Sci U S A* 113**,** E500-508.

Jacobsen, L.K., Mencl, W.E., Westerveld, M., and Pugh, K.R. (2004). Impact of cannabis use on brain function in adolescents. *Ann N Y Acad Sci* 1021**,** 384-390.

Jacobus, J., Castro, N., Squeglia, L.M., Meloy, M.J., Brumback, T., Huestis, M.A., and Tapert, S.F. (2016). Adolescent cortical thickness pre- and post marijuana and alcohol initiation. *Neurotoxicol Teratol* 57**,** 20-29.

Jacobus, J., Mcqueeny, T., Bava, S., Schweinsburg, B.C., Frank, L.R., Yang, T.T., and Tapert, S.F. (2009). White matter integrity in adolescents with histories of marijuana use and binge drinking. *Neurotoxicol Teratol* 31**,** 349-355.

Jacobus, J., Squeglia, L.M., Bava, S., and Tapert, S.F. (2013a). White matter characterization of adolescent binge drinking with and without co-occurring marijuana use: a 3-year investigation. *Psychiatry Res* 214**,** 374-381.

Jacobus, J., Squeglia, L.M., Infante, M.A., Bava, S., and Tapert, S.F. (2013b). White matter integrity pre- and post marijuana and alcohol initiation in adolescence. *Brain Sci* 3**,** 396-414.

Jacobus, J., Squeglia, L.M., Meruelo, A.D., Castro, N., Brumback, T., Giedd, J.N., and Tapert, S.F. (2015). Cortical thickness in adolescent marijuana and alcohol users: A three-year prospective study from adolescence to young adulthood. *Dev Cogn Neurosci* 16**,** 101-109.

Jacobus, J., Squeglia, L.M., Sorg, S.F., Nguyen-Louie, T.T., and Tapert, S.F. (2014). Cortical thickness and neurocognition in adolescent marijuana and alcohol users following 28 days of monitored abstinence. *J Stud Alcohol Drugs* 75**,** 729-743.

Jager, G., Block, R.I., Luijten, M., and Ramsey, N.F. (2010). Cannabis use and memory brain function in adolescent boys: a cross-sectional multicenter functional magnetic resonance imaging study. *J Am Acad Child Adolesc Psychiatry* 49**,** 561-572, 572 e561-563.

Jager, G., Block, R.I., Luijten, M., and Ramsey, N.F. (2013). Tentative evidence for striatal hyperactivity in adolescent cannabis-using boys: a cross-sectional multicenter fMRI study. *J Psychoactive Drugs* 45**,** 156-167.

Jin, L.Z., Rangan, A., Mehlsen, J., Andersen, L.B., Larsen, S.C., and Heitmann, B.L. (2017). Association Between Use of Cannabis in Adolescence and Weight Change into Midlife. *PLoS One* 12**,** e0168897.

Jones, H.J., Gage, S.H., Heron, J., Hickman, M., Lewis, G., Munafo, M.R., and Zammit, S. (2018). Association of Combined Patterns of Tobacco and Cannabis Use in Adolescence With Psychotic Experiences. *JAMA Psychiatry* 75**,** 240-246.

Jones, S.A., Cservenka, A., and Nagel, B.J. (2016). Binge drinking impacts dorsal striatal response during decision making in adolescents. *Neuroimage* 129**,** 378-388.

Jones, S.A., Steele, J.S., and Nagel, B.J. (2017). Binge drinking and family history of alcoholism are associated with an altered developmental trajectory of impulsive choice across adolescence. *Addiction* 112**,** 1184-1192.

Jurk, S., Mennigen, E., Goschke, T., and Smolka, M.N. (2018). Low-level alcohol consumption during adolescence and its impact on cognitive control development. *Addict Biol* 23**,** 313-326.

Kaasbøll, C., Hagen, R., and Gråwe, R.W. (2018). Population-Based Associations Among Cannabis Use, Anxiety, and Depression in Norwegian Adolescents. *Journal of Child & Adolescent Substance Abuse* 27**,** 238-243.

Kaplow, J.B., Curran, P.J., Angold, A., and Costello, E.J. (2001). The prospective relation between dimensions of anxiety and the initiation of adolescent alcohol use. *J Clin Child Psychol* 30**,** 316-326.

Karoly, H.C., Bryan, A.D., Weiland, B.J., Mayer, A., Dodd, A., and Feldstein Ewing, S.W. (2015). Does incentive-elicited nucleus accumbens activation differ by substance of abuse? An examination with adolescents. *Dev Cogn Neurosci* 16**,** 5-15.

Khurana, A., Romer, D., Betancourt, L.M., Brodsky, N.L., Giannetta, J.M., and Hurt, H. (2013). Working memory ability predicts trajectories of early alcohol use in adolescents: the mediational role of impulsivity. *Addiction* 108**,** 506-515.

Kumra, S., Robinson, P., Tambyraja, R., Jensen, D., Schimunek, C., Houri, A., Reis, T., and Lim, K. (2012). Parietal lobe volume deficits in adolescents with schizophrenia and adolescents with cannabis use disorders. *J Am Acad Child Adolesc Psychiatry* 51**,** 171-180.

Lane, S.D., Cherek, D.R., Pietras, C.J., and Steinberg, J.L. (2005). Performance of heavy marijuana-smoking adolescents on a laboratory measure of motivation. *Addict Behav* 30**,** 815-828.

Lane, S.D., Cherek, D.R., Tcheremissine, O.V., Steinberg, J.L., and Sharon, J.L. (2007). Response perseveration and adaptation in heavy marijuana-smoking adolescents. *Addict Behav* 32**,** 977-990.

Lichenstein, S.D., Musselman, S., Shaw, D.S., Sitnick, S., and Forbes, E.E. (2017). Nucleus accumbens functional connectivity at age 20 is associated with trajectory of adolescent cannabis use and predicts psychosocial functioning in young adulthood. *Addiction* 112**,** 1961-1970.

Lisdahl, K.M., Thayer, R., Squeglia, L.M., Mcqueeny, T.M., and Tapert, S.F. (2013). Recent binge drinking predicts smaller cerebellar volumes in adolescents. *Psychiatry Res* 211**,** 17-23.

Luciana, M., Collins, P.F., Muetzel, R.L., and Lim, K.O. (2013). Effects of alcohol use initiation on brain structure in typically developing adolescents. *Am J Drug Alcohol Abuse* 39**,** 345-355.

Mahedy, L., Field, M., Gage, S., Hammerton, G., Heron, J., Hickman, M., and Munafo, M.R. (2018). Alcohol Use in Adolescence and Later Working Memory: Findings From a Large Population-Based Birth Cohort. *Alcohol Alcohol* 53**,** 251-258.

Mahmood, O.M., Jacobus, J., Bava, S., Scarlett, A., and Tapert, S.F. (2010). Learning and memory performances in adolescent users of alcohol and marijuana: interactive effects. *J Stud Alcohol Drugs* 71**,** 885-894.

Mason, W.A., Kosterman, R., Haggerty, K.P., Hawkins, J.D., Redmond, C., Spoth, R.L., and Shin, C. (2008). Dimensions of adolescent alcohol involvement as predictors of young-adult major depression. *J Stud Alcohol Drugs* 69**,** 275-285.

Matuszka, B., Bácskai, E., Czobor, P., and Gerevich, J. (2016). Physical Aggression and Concurrent Alcohol and Tobacco Use Among Adolescents. *International Journal of Mental Health and Addiction* 15**,** 90-99.

Mcqueeny, T., Padula, C.B., Price, J., Medina, K.L., Logan, P., and Tapert, S.F. (2011). Gender effects on amygdala morphometry in adolescent marijuana users. *Behav Brain Res* 224**,** 128-134.

Mcqueeny, T., Schweinsburg, B.C., Schweinsburg, A.D., Jacobus, J., Bava, S., Frank, L.R., and Tapert, S.F. (2009). Altered white matter integrity in adolescent binge drinkers. *Alcohol Clin Exp Res* 33**,** 1278-1285.

Medina, K.L., Hanson, K.L., Schweinsburg, A.D., Cohen-Zion, M., Nagel, B.J., and Tapert, S.F. (2007a). Neuropsychological functioning in adolescent marijuana users: subtle deficits detectable after a month of abstinence. *J Int Neuropsychol Soc* 13**,** 807-820.

Medina, K.L., Mcqueeny, T., Nagel, B.J., Hanson, K.L., Yang, T.T., and Tapert, S.F. (2009). Prefrontal cortex morphometry in abstinent adolescent marijuana users: subtle gender effects. *Addict Biol* 14**,** 457-468.

Medina, K.L., Nagel, B.J., Park, A., Mcqueeny, T., and Tapert, S.F. (2007b). Depressive symptoms in adolescents: associations with white matter volume and marijuana use. *J Child Psychol Psychiatry* 48**,** 592-600.

Medina, K.L., Nagel, B.J., and Tapert, S.F. (2010). Abnormal cerebellar morphometry in abstinent adolescent marijuana users. *Psychiatry Res* 182**,** 152-159.

Medina, K.L., Schweinsburg, A.D., Cohen-Zion, M., Nagel, B.J., and Tapert, S.F. (2007c). Effects of alcohol and combined marijuana and alcohol use during adolescence on hippocampal volume and asymmetry. *Neurotoxicol Teratol* 29**,** 141-152.

Meier, M.H., Caspi, A., Ambler, A., Harrington, H., Houts, R., Keefe, R.S., Mcdonald, K., Ward, A., Poulton, R., and Moffitt, T.E. (2012). Persistent cannabis users show neuropsychological decline from childhood to midlife. *Proc Natl Acad Sci U S A* 109**,** E2657-2664.

Meier, M.H., Caspi, A., Danese, A., Fisher, H.L., Houts, R., Arseneault, L., and Moffitt, T.E. (2018). Associations between adolescent cannabis use and neuropsychological decline: a longitudinal co-twin control study. *Addiction* 113**,** 257-265.

Meier, M.H., Schriber, R.A., Beardslee, J., Hanson, J., and Pardini, D. (2019). Associations between adolescent cannabis use frequency and adult brain structure: A prospective study of boys followed to adulthood. *Drug Alcohol Depend* 202**,** 191-199.

Melaugh Mcateer, A., Curran, D., and Hanna, D. (2015). Alcohol attention bias in adolescent social drinkers: an eye tracking study. *Psychopharmacology (Berl)* 232**,** 3183-3191.

Miech, R., Johnston, L., O'malley, P.M., Keyes, K.M., and Heard, K. (2015). Prescription Opioids in Adolescence and Future Opioid Misuse. *Pediatrics* 136**,** e1169-1177.

Mokrysz, C., Landy, R., Gage, S.H., Munafo, M.R., Roiser, J.P., and Curran, H.V. (2016). Are IQ and educational outcomes in teenagers related to their cannabis use? A prospective cohort study. *J Psychopharmacol* 30**,** 159-168.

Morales, A.M., Jones, S.A., Ehlers, A., Lavine, J.B., and Nagel, B.J. (2018). Ventral striatal response during decision making involving risk and reward is associated with future binge drinking in adolescents. *Neuropsychopharmacology* 43**,** 1884-1890.

Morin, J.G., Afzali, M.H., Bourque, J., Stewart, S.H., Seguin, J.R., O'leary-Barrett, M., and Conrod, P.J. (2019). A Population-Based Analysis of the Relationship Between Substance Use and Adolescent Cognitive Development. *Am J Psychiatry* 176**,** 98-106.

Nagel, B.J., Schweinsburg, A.D., Phan, V., and Tapert, S.F. (2005). Reduced hippocampal volume among adolescents with alcohol use disorders without psychiatric comorbidity. *Psychiatry Res* 139**,** 181-190.

Nestor, L.J., Behan, B., Suckling, J., and Garavan, H. (2019). Cannabis-dependent adolescents show differences in global reward-associated network topology: A functional connectomics approach. *Addict Biol***,** e12752.

Nguyen-Louie, T.T., Matt, G.E., Jacobus, J., Li, I., Cota, C., Castro, N., and Tapert, S.F. (2017). Earlier Alcohol Use Onset Predicts Poorer Neuropsychological Functioning in Young Adults. *Alcohol Clin Exp Res* 41**,** 2082-2092.

Nguyen-Louie, T.T., Tracas, A., Squeglia, L.M., Matt, G.E., Eberson-Shumate, S., and Tapert, S.F. (2016). Learning and Memory in Adolescent Moderate, Binge, and Extreme-Binge Drinkers. *Alcohol Clin Exp Res* 40**,** 1895-1904.

Noorbakhsh, S., Afzali, M.H., Boers, E., and Conrod, P.J. (2020). Cognitive Function Impairments Linked to Alcohol and Cannabis Use During Adolescence: A Study of Gender Differences. *Front Hum Neurosci* 14**,** 95.

Norman, A.L., Pulido, C., Squeglia, L.M., Spadoni, A.D., Paulus, M.P., and Tapert, S.F. (2011). Neural activation during inhibition predicts initiation of substance use in adolescence. *Drug Alcohol Depend* 119**,** 216-223.

Orr, C., Spechler, P., Cao, Z., Albaugh, M., Chaarani, B., Mackey, S., D'souza, D., Allgaier, N., Banaschewski, T., Bokde, A.L.W., Bromberg, U., Buchel, C., Burke Quinlan, E., Conrod, P., Desrivieres, S., Flor, H., Frouin, V., Gowland, P., Heinz, A., Ittermann, B., Martinot, J.L., Martinot, M.P., Nees, F., Papadopoulos Orfanos, D., Paus, T., Poustka, L., Millenet, S., Frohner, J.H., Radhakrishnan, R., Smolka, M.N., Walter, H., Whelan, R., Schumann, G., Potter, A., and Garavan, H. (2019). Grey Matter Volume Differences Associated with Extremely Low Levels of Cannabis Use in Adolescence. *J Neurosci* 39**,** 1817-1827.

Padula, C.B., Schweinsburg, A.D., and Tapert, S.F. (2007). Spatial working memory performance and fMRI activation interaction in abstinent adolescent marijuana users. *Psychol Addict Behav* 21**,** 478-487.

Pampati, S., Buu, A., Hu, Y.H., Mendes De Leon, C.F., and Lin, H.C. (2018). Effects of alcohol and cigarette use on the initiation, reinitiation, and persistence of cannabis use from adolescence to emerging adulthood. *Addict Behav* 79**,** 144-150.

Parada, M., Corral, M., Mota, N., Crego, A., Rodriguez Holguin, S., and Cadaveira, F. (2012). Executive functioning and alcohol binge drinking in university students. *Addict Behav* 37**,** 167-172.

Parrish, K.H., Atherton, O.E., Quintana, A., Conger, R.D., and Robins, R.W. (2016). Reciprocal relations between internalizing symptoms and frequency of alcohol use: Findings from a longitudinal study of Mexican-origin youth. *Psychol Addict Behav* 30**,** 203-208.

Patrick, M.E., Kloska, D.D., Terry-Mcelrath, Y.M., Lee, C.M., O'malley, P.M., and Johnston, L.D. (2018). Patterns of simultaneous and concurrent alcohol and marijuana use among adolescents. *Am J Drug Alcohol Abuse* 44**,** 441-451.

Peters, S., Peper, J.S., Van Duijvenvoorde, A.C.K., Braams, B.R., and Crone, E.A. (2017). Amygdala-orbitofrontal connectivity predicts alcohol use two years later: a longitudinal neuroimaging study on alcohol use in adolescence. *Dev Sci* 20.

Pfefferbaum, A., Kwon, D., Brumback, T., Thompson, W.K., Cummins, K., Tapert, S.F., Brown, S.A., Colrain, I.M., Baker, F.C., Prouty, D., De Bellis, M.D., Clark, D.B., Nagel, B.J., Chu, W., Park, S.H., Pohl, K.M., and Sullivan, E.V. (2018). Altered Brain Developmental Trajectories in Adolescents After Initiating Drinking. *Am J Psychiatry* 175**,** 370-380.

Pope, H.G., Jr., Gruber, A.J., Hudson, J.I., Cohane, G., Huestis, M.A., and Yurgelun-Todd, D. (2003). Early-onset cannabis use and cognitive deficits: what is the nature of the association? *Drug Alcohol Depend* 69**,** 303-310.

Ruan, H., Zhou, Y., Luo, Q., Robert, G.H., Desrivieres, S., Quinlan, E.B., Liu, Z., Banaschewski, T., Bokde, A.L.W., Bromberg, U., Buchel, C., Flor, H., Frouin, V., Garavan, H., Gowland, P., Heinz, A., Ittermann, B., Martinot, J.L., Martinot, M.P., Nees, F., Orfanos, D.P., Poustka, L., Hohmann, S., Frohner, J.H., Smolka, M.N., Walter, H., Whelan, R., Li, F., Schumann, G., Feng, J., and Consortium, I. (2019). Adolescent binge drinking disrupts normal trajectories of brain functional organization and personality maturation. *Neuroimage Clin* 22**,** 101804.

Rubinstein, M.L., Rait, M.A., and Prochaska, J.J. (2014). Frequent marijuana use is associated with greater nicotine addiction in adolescent smokers. *Drug Alcohol Depend* 141**,** 159-162.

Schleider, J.L., Ye, F., Wang, F., Hipwell, A.E., Chung, T., and Sartor, C.E. (2019). Longitudinal Reciprocal Associations Between Anxiety, Depression, and Alcohol Use in Adolescent Girls. *Alcohol Clin Exp Res* 43**,** 98-107.

Schmid, B., Hohm, E., Blomeyer, D., Zimmermann, U.S., Schmidt, M.H., Esser, G., and Laucht, M. (2007). Concurrent alcohol and tobacco use during early adolescence characterizes a group at risk. *Alcohol Alcohol* 42**,** 219-225.

Scholes-Balog, K.E., Hemphill, S.A., Evans-Whipp, T.J., Toumbourou, J.W., and Patton, G.C. (2016). Developmental trajectories of adolescent cannabis use and their relationship to young adult social and behavioural adjustment: A longitudinal study of Australian youth. *Addict Behav* 53**,** 11-18.

Schweinsburg, A.D., Mcqueeny, T., Nagel, B.J., Eyler, L.T., and Tapert, S.F. (2010). A preliminary study of functional magnetic resonance imaging response during verbal encoding among adolescent binge drinkers. *Alcohol* 44**,** 111-117.

Schweinsburg, A.D., Schweinsburg, B.C., Cheung, E.H., Brown, G.G., Brown, S.A., and Tapert, S.F. (2005). fMRI response to spatial working memory in adolescents with comorbid marijuana and alcohol use disorders. *Drug Alcohol Depend* 79**,** 201-210.

Schweinsburg, A.D., Schweinsburg, B.C., Nagel, B.J., Eyler, L.T., and Tapert, S.F. (2011). Neural correlates of verbal learning in adolescent alcohol and marijuana users. *Addiction* 106**,** 564-573.

Scott, J.C., Rosen, A.F.G., Moore, T.M., Roalf, D.R., Satterthwaite, T.D., Calkins, M.E., Ruparel, K., Gur, R.E., and Gur, R.C. (2019). Cannabis use in youth is associated with limited alterations in brain structure. *Neuropsychopharmacology* 44**,** 1362-1369.

Scott, J.C., Wolf, D.H., Calkins, M.E., Bach, E.C., Weidner, J., Ruparel, K., Moore, T.M., Jones, J.D., Jackson, C.T., Gur, R.E., and Gur, R.C. (2017). Cognitive functioning of adolescent and young adult cannabis users in the Philadelphia Neurodevelopmental Cohort. *Psychol Addict Behav* 31**,** 423-434.

Shahzade, C., Chun, J., Delisi, L.E., and Manschreck, T.C. (2018). Patterns in adolescent cannabis use predict the onset and symptom structure of schizophrenia-spectrum disorder. *Schizophr Res* 197**,** 539-543.

Solowij, N., Jones, K.A., Rozman, M.E., Davis, S.M., Ciarrochi, J., Heaven, P.C., Pesa, N., Lubman, D.I., and Yucel, M. (2012). Reflection impulsivity in adolescent cannabis users: a comparison with alcohol-using and non-substance-using adolescents. *Psychopharmacology (Berl)* 219**,** 575-586.

Spechler, P.A., Orr, C.A., Chaarani, B., Kan, K.J., Mackey, S., Morton, A., Snowe, M.P., Hudson, K.E., Althoff, R.R., Higgins, S.T., Cattrell, A., Flor, H., Nees, F., Banaschewski, T., Bokde, A.L.W., Whelan, R., Buchel, C., Bromberg, U., Conrod, P., Frouin, V., Papadopoulos, D., Gallinat, J., Heinz, A., Walter, H., Ittermann, B., Gowland, P., Paus, T., Poustka, L., Martinot, J.L., Artiges, E., Smolka, M.N., Schumann, G., Garavan, H., and Consortium, I. (2015). Cannabis use in early adolescence: Evidence of amygdala hypersensitivity to signals of threat. *Dev Cogn Neurosci* 16**,** 63-70.

Squeglia, L.M., Pulido, C., Wetherill, R.R., Jacobus, J., Brown, G.G., and Tapert, S.F. (2012a). Brain response to working memory over three years of adolescence: influence of initiating heavy drinking. *J Stud Alcohol Drugs* 73**,** 749-760.

Squeglia, L.M., Rinker, D.A., Bartsch, H., Castro, N., Chung, Y., Dale, A.M., Jernigan, T.L., and Tapert, S.F. (2014). Brain volume reductions in adolescent heavy drinkers. *Dev Cogn Neurosci* 9**,** 117-125.

Squeglia, L.M., Schweinsburg, A.D., Pulido, C., and Tapert, S.F. (2011). Adolescent binge drinking linked to abnormal spatial working memory brain activation: differential gender effects. *Alcohol Clin Exp Res* 35**,** 1831-1841.

Squeglia, L.M., Sorg, S.F., Schweinsburg, A.D., Wetherill, R.R., Pulido, C., and Tapert, S.F. (2012b). Binge drinking differentially affects adolescent male and female brain morphometry. *Psychopharmacology (Berl)* 220**,** 529-539.

Squeglia, L.M., Spadoni, A.D., Infante, M.A., Myers, M.G., and Tapert, S.F. (2009). Initiating moderate to heavy alcohol use predicts changes in neuropsychological functioning for adolescent girls and boys. *Psychol Addict Behav* 23**,** 715-722.

Subramaniam, G.A., and Stitzer, M.A. (2009). Clinical characteristics of treatment-seeking prescription opioid vs. heroin-using adolescents with opioid use disorder. *Drug Alcohol Depend* 101**,** 13-19.

Subramaniam, G.A., Stitzer, M.L., Woody, G., Fishman, M.J., and Kolodner, K. (2009). Clinical characteristics of treatment-seeking adolescents with opioid versus cannabis/alcohol use disorders. *Drug Alcohol Depend* 99**,** 141-149.

Subramaniam, P., Rogowska, J., Dimuzio, J., Lopez-Larson, M., Mcglade, E., and Yurgelun-Todd, D. (2018). Orbitofrontal connectivity is associated with depression and anxiety in marijuana-using adolescents. *J Affect Disord* 239**,** 234-241.

Swartz, J.R., Weissman, D.G., Ferrer, E., Beard, S.J., Fassbender, C., Robins, R.W., Hastings, P.D., and Guyer, A.E. (2020). Reward-Related Brain Activity Prospectively Predicts Increases in Alcohol Use in Adolescents. *J Am Acad Child Adolesc Psychiatry* 59**,** 391-400.

Swift, W., Coffey, C., Degenhardt, L., Carlin, J.B., Romaniuk, H., and Patton, G.C. (2012). Cannabis and progression to other substance use in young adults: findings from a 13-year prospective population-based study. *J Epidemiol Community Health* 66**,** e26.

Tapert, S.F., Cheung, E.H., Brown, G.G., Frank, L.R., Paulus, M.P., Schweinsburg, A.D., Meloy, M.J., and Brown, S.A. (2003). Neural response to alcohol stimuli in adolescents with alcohol use disorder. *Arch Gen Psychiatry* 60**,** 727-735.

Tapert, S.F., Schweinsburg, A.D., Barlett, V.C., Brown, S.A., Frank, L.R., Brown, G.G., and Meloy, M.J. (2004). Blood oxygen level dependent response and spatial working memory in adolescents with alcohol use disorders. *Alcohol Clin Exp Res* 28**,** 1577-1586.

Tapert, S.F., Schweinsburg, A.D., Drummond, S.P., Paulus, M.P., Brown, S.A., Yang, T.T., and Frank, L.R. (2007). Functional MRI of inhibitory processing in abstinent adolescent marijuana users. *Psychopharmacology (Berl)* 194**,** 173-183.

Taylor, M., Collin, S.M., Munafo, M.R., Macleod, J., Hickman, M., and Heron, J. (2017). Patterns of cannabis use during adolescence and their association with harmful substance use behaviour: findings from a UK birth cohort. *J Epidemiol Community Health* 71**,** 764-770.

Terry-Mcelrath, Y.M., O'malley, P.M., and Johnston, L.D. (2014). Alcohol and marijuana use patterns associated with unsafe driving among U.S. high school seniors: high use frequency, concurrent use, and simultaneous use. *J Stud Alcohol Drugs* 75**,** 378-389.

Tervo-Clemmens, B., Simmonds, D., Calabro, F.J., Day, N.L., Richardson, G.A., and Luna, B. (2018). Adolescent cannabis use and brain systems supporting adult working memory encoding, maintenance, and retrieval. *Neuroimage* 169**,** 496-509.

Viner, R.M., and Taylor, B. (2007). Adult outcomes of binge drinking in adolescence: findings from a UK national birth cohort. *J Epidemiol Community Health* 61**,** 902-907.

Vo, H.T., Schacht, R., Mintzer, M., and Fishman, M. (2014). Working memory impairment in cannabis- and opioid-dependent adolescents. *Subst Abus* 35**,** 387-390.

Weiland, B.J., Thayer, R.E., Depue, B.E., Sabbineni, A., Bryan, A.D., and Hutchison, K.E. (2015). Daily marijuana use is not associated with brain morphometric measures in adolescents or adults. *J Neurosci* 35**,** 1505-1512.

Wetherill, R.R., Squeglia, L.M., Yang, T.T., and Tapert, S.F. (2013). A longitudinal examination of adolescent response inhibition: neural differences before and after the initiation of heavy drinking. *Psychopharmacology (Berl)* 230**,** 663-671.

Wilson, W., Mathew, R., Turkington, T., Hawk, T., Coleman, R.E., and Provenzale, J. (2000). Brain morphological changes and early marijuana use: a magnetic resonance and positron emission tomography study. *J Addict Dis* 19**,** 1-22.

Winward, J.L., Hanson, K.L., Tapert, S.F., and Brown, S.A. (2014). Heavy alcohol use, marijuana use, and concomitant use by adolescents are associated with unique and shared cognitive decrements. *J Int Neuropsychol Soc* 20**,** 784-795.

Womack, S.R., Shaw, D.S., Weaver, C.M., and Forbes, E.E. (2016). Bidirectional Associations Between Cannabis Use and Depressive Symptoms From Adolescence Through Early Adulthood Among At-Risk Young Men. *J Stud Alcohol Drugs* 77**,** 287-297.
